# Sparse Distributed Representation of Odors in a Large-scale Olfactory Bulb Circuit

**DOI:** 10.1371/journal.pcbi.1003014

**Published:** 2013-03-28

**Authors:** Yuguo Yu, Thomas S. McTavish, Michael L. Hines, Gordon M. Shepherd, Cesare Valenti, Michele Migliore

**Affiliations:** 1 Centre for Computational Systems Biology, School of Life Sciences, Fudan University, Shanghai, People's Republic of China; 2 Department of Neurobiology, Yale University School of Medicine, New Haven, Connecticut, United States of America; 3 Department of Mathematics and Informatics, University of Palermo, Palermo, Italy; 4 Institute of Biophysics, National Research Council, Palermo, Italy; Université Paris Descartes, Centre National de la Recherche Scientifique, France

## Abstract

In the olfactory bulb, lateral inhibition mediated by granule cells has been suggested to modulate the timing of mitral cell firing, thereby shaping the representation of input odorants. Current experimental techniques, however, do not enable a clear study of how the mitral-granule cell network sculpts odor inputs to represent odor information spatially and temporally. To address this critical step in the neural basis of odor recognition, we built a biophysical network model of mitral and granule cells, corresponding to 1/100th of the real system in the rat, and used direct experimental imaging data of glomeruli activated by various odors. The model allows the systematic investigation and generation of testable hypotheses of the functional mechanisms underlying odor representation in the olfactory bulb circuit. Specifically, we demonstrate that lateral inhibition emerges within the olfactory bulb network through recurrent dendrodendritic synapses when constrained by a range of balanced excitatory and inhibitory conductances. We find that the spatio-temporal dynamics of lateral inhibition plays a critical role in building the glomerular-related cell clusters observed in experiments, through the modulation of synaptic weights during odor training. Lateral inhibition also mediates the development of sparse and synchronized spiking patterns of mitral cells related to odor inputs within the network, with the frequency of these synchronized spiking patterns also modulated by the sniff cycle.

## Author Summary

In the paper we address the role of lateral inhibition in a neuronal network. It is an essential and widespread mechanism of neural processing that has been demonstrated in many brain systems. A key finding that would reveal how and to what extent it can modulate input signals and give rise to some form of perception would involve network-wide recording of individual cells during *in vivo* behavioral experiments. While this problem has been intensely investigated, it is beyond current methods to record from a reasonable set of cells experimentally to decipher the emergent properties and behavior of the network, leaving the underlying computational and functional roles of lateral inhibition still poorly understood. We addressed this problem using a large-scale model of the olfactory bulb. The model demonstrates how lateral inhibition modulates the evolving dynamics of the olfactory bulb network, generating mitral and granule cell responses that account for critical experimental findings. It also suggests how odor identity can be represented by a combination of temporal and spatial patterns of mitral cell activity, with both feedforward excitation and lateral inhibition via dendrodendritic synapses as the underlying mechanisms facilitating network self-organization and the emergence of synchronized oscillations.

## Introduction

Lateral inhibition is one of the critical mechanisms underlying responses to sensory neurons [1], but the detailed mechanisms at the network level in the olfactory system are not clear [e.g. 2]. In the Limulus eye [1] and the cat retina [3] it mediates contrast enhancement between areas of differing illumination. It has also been found in the auditory pathway (reviewed in [4]) and the somatosensory system [5]. In the olfactory system, the clearest evidence for lateral inhibition is the interaction between mitral cells in the olfactory bulb, mediated through inhibitory granule cells [6]–[7] and periglomerular cells [8]. The possible underlying circuits and their computational properties have been widely investigated experimentally [9]–[11] especially in terms of odor selectivity and dynamics of mitral cell responses [12]–[14]. A major problem in interpreting the experimental findings *in vivo* is that they are usually obtained in single cells or in small randomly selected sets of cells, whereas a clear understanding of fundamental processes, such as the spatio-temporal organization of the mitral-granule cell network, requires simultaneous recording from a relevant subset of cells activated by any given odor. The functional effects of network-wide processes, in relation to the patterns of glomeruli activated by different odors, therefore remain relatively unknown and extremely difficult to explore experimentally.

To gain insight into this problem we have focused on mitral-granule cell interactions, the best understood circuit in the olfactory bulb. For this purpose we have constructed a biophysical network model of multicompartment mitral and granule cells with connections similar to those in the real olfactory bulb. As input we have used the activation of individual glomeruli by a large set of odors identified by intrinsic imaging [15]. This model has allowed us to investigate several fundamental questions: 1) How does the network self-organize and modulate mitral cell responses to different odor molecules? Lateral inhibition has been suggested to be the primary mechanism. However, experimentally the focus is almost exclusively on individual mitral and granule cell interactions, and whether lateral inhibition is able to shape network-wide connectivity is not known. 2) How does the precise timing of mitral cell action potentials subject to lateral inhibition relate to the sniff cycle (as shown in [16])? This appears to be one of the critical processes affecting responses in awake mice [2], but the mechanisms responsible for the firing behavior at the network level are not understood. 3) Why is the olfactory bulb network connectivity sparse and distributed (as shown in [10] and [17])? There are no experimental data for describing the underlying mechanism. We have previously proposed a physiologically plausible process using a small network with simulated odors and all-to-all possible connectivity [18]. Its validity for more realistic odor inputs, network size, and intrinsically sparse connectivity required testing, which was carried out in the present study.

The results show that: 1) Lateral inhibition mediated through broadly tuned granule cells results in mitral cell output that reflects the spatial distribution of the glomerular input and the temporal structure of the inhibitory processing. Together, the mitral-granule cell circuit operates to generate a unique spatiotemporal representation for each odor. 2) Odor identity can emerge from a single sniff as a specific distribution of spikes in which each mitral cell makes its own contribution according to the specific type of input it receives and the network of granule cells it activates. 3) The mitral<->granule cell interactions through dendrodendritic synapses can account for the distributed network connectivity observed experimentally. We discuss how these specific predictions from the model provide new hypotheses for experimental testing.

## Results

**Figure 1:**
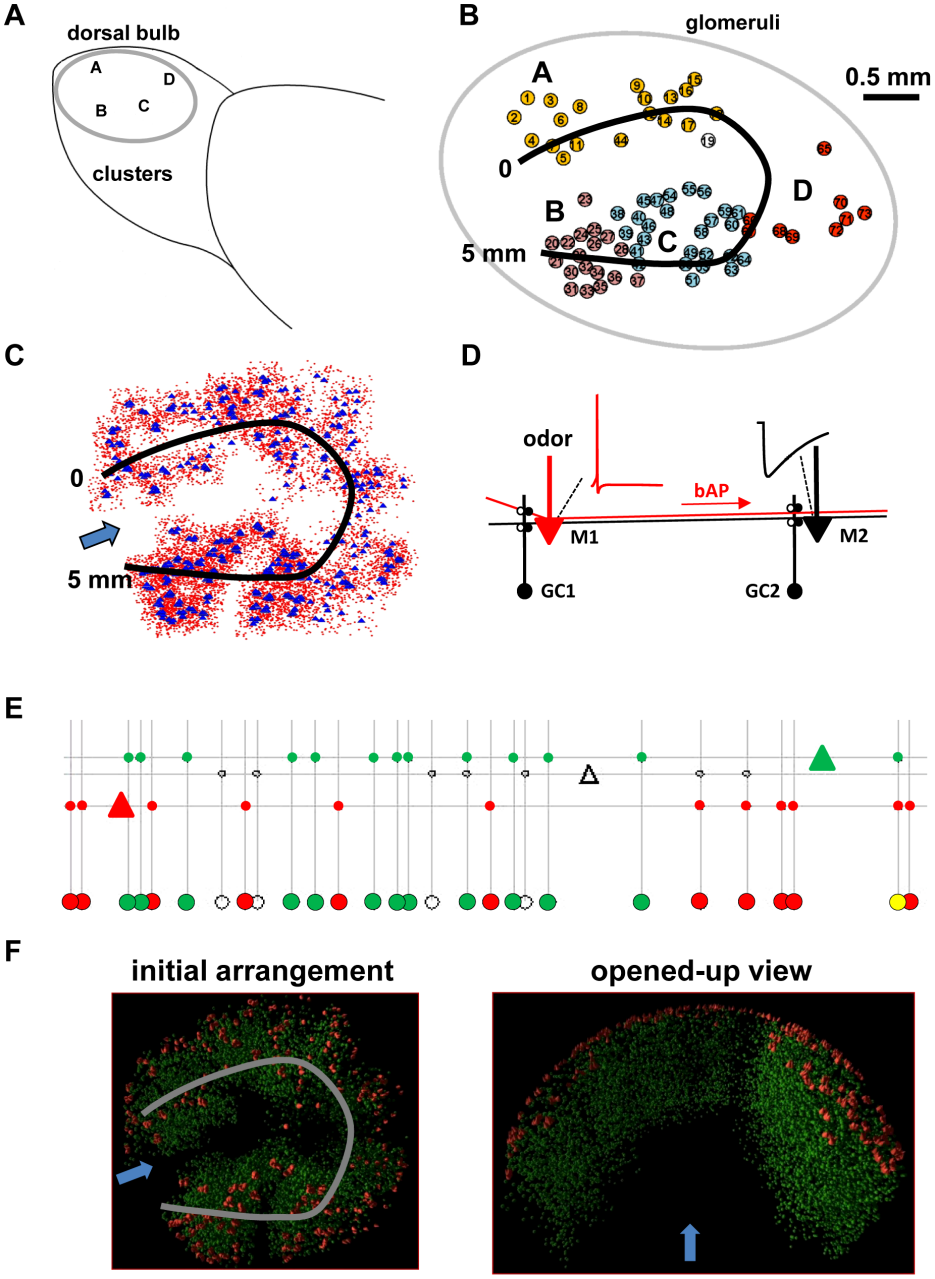
Schematic description of the model and its relation to *in vivo* experimental data. **A**) Dorsal surface of the olfactory bulb from which experimental data were obtained; different letters identify different clusters of glomeruli responding to different molecular features of the odor molecules; in this paper we used glomeruli activated in clusters A–D. **B**) Spatial distribution of the 73 glomeruli activated by the 72 odors used in this work; the black line represents the projection track used to reduce the system to 1 dimension (see main text for details). **C**) Top view of the soma spatial locations for the 500 mitral cells (blue triangles) and 10000 granule cells (red circles) used in all simulations; the black line is used to reduce the system to 1 dimension, and the arrow indicates the viewpoint used for all simulation movies. **D**) The connectivity for an efficient lateral inhibition from non-topographical connectivity of the granule-mitral cell network (see text). The basic microcircuit is schematically represented with two mitral (M1 and M2), two granule cells (GC1 and GC2), and their dendrodendritic reciprocal synapses (open and closed circles represent excitatory and inhibitory synapses, respectively). The somatic action potential elicited in M1 by an odor (red trace close to M1 soma) backpropagates (*bAP*) along the M1 lateral dendrite (in red). This activates GC2 and a consequent local inhibitory potential (black trace) close to the soma of M2. In this way inhibition can be independent of distance, imposed locally by granule cells activated by backpropagating action potentials. **E**) In the current model, the ratio of granule to mitral cells is 20∶1, with a 10% probability of connection, as schematically represented in the figure (triangles, mitral cells; large circles, granule cells; small circles, reciprocal synapses) for two different mitral cells (green and red); the yellow circle represents a granule cell which receives input from both the red and green mitral cells, whereas unfilled symbols represent mitral, granule, and reciprocal synapses not connected with either the green or red mitral cells. **F**) To appreciate more clearly the network activity, a movie was generated from each simulation, with the mitral cell somas laid out using {X,Y} coordinates reflecting the spatial location of the real glomeruli in the dorsal surface investigated in the experiments, and granule cell somas distributed inside a 3D space schematically representing their spatial organization in the real olfactory bulb (*initial arrangement* panel). An opened-out view of the system was used as the viewpoint to display somatic spikes during a simulation (*opened-up view* panel); the gray line represents the projection track used to reduce the system to 1 dimension, and the arrow indicates the viewpoint used for all simulation movies. See movie S1 and its full HD version on the ModelDB database (acc.n. 144570).

The input to the network model was based on experimental data obtained by Mori et al. (2006) from 17 male rats using intrinsic imaging of activity induced in the olfactory glomeruli by different homologous series of chemically related odor molecules. That report showed that a given odor molecule tended to elicit glomerular responses in a given part of the olfactory bulb, and that responses to related odor molecules tended to be near each other. The authors defined these related glomeruli as a “cluster”; equivalent to “domain” as used by other authors [19]–[20]. Different clusters, labeled from A to I located on the dorsal and lateral surfaces of the rat olfactory bulb, are summarized in Fig. 1A.

The present model of the mitral-granule cell network that would process the inputs from these clusters, assumes preprocessing by local glomerular circuits (reviewed in [21]). We began by rendering the 74 contiguous clusters of A through D in a two-dimensional map as shown in Fig. 1B. While many mitral cells have been visually labeled, a 3D reconstruction of the mitral cells belonging to a glomerulus, and moreover, the dendritic relationships between mitral cells of different glomeruli, is not known. Therefore, a complete 3- and 2-dimensional reconstruction of mitral cell interactions via the lateral dendrites is unavailable. To obtain insight into the rules of connectivity, we simplified the relations to 1 dimension. To do this, we projected the 74 glomeruli onto a single track that passed close to most of them as shown by the bold line in Fig. 1B. Having the track complete a circle also avoided edge effects in the model network. We then represented the population of mitral and granule cells within these clusters. It has been estimated [22]–[23] that in the rat the glomeruli, mitral cells and granule cells are in a ratio of approximately 1∶15∶500. To remain computationally tractable, we used a ratio of 1 glomerulus, 5 mitral cells and 100 granule cells. This enabled us to analyze the mitral-granule interactions without having it overwhelmed by the sheer numbers of mitral and especially granule cells. The 74 glomeruli thus corresponded to 370 mitral cells and 7400 granule cells. We increased the numbers to 500 mitral and 10,000 granule cells by randomly adding the cells between the clusters. Their somas were laid out using {X,Y} coordinates reflecting the spatial location of the real glomeruli in the dorsal surface investigated in the experiments (Fig. 1B), and topologically aligned along the 5 mm linear space (black line in Figs. 1B,C). The final populations and their relations to the track are shown in Fig. 1C. An obvious limitation of the one-dimensional approach is that many glomeruli are at variable distances from the single projection tract, so that interactions between mitral cells belonging to specific neighboring glomeruli are not precisely represented. However, the interactions of a given mitral cell with many nearby mitral cells still holds in a generic sense, so that the model gives an accurate reflection of these population interactions within the mitral-granule network.

The connectivity between mitral and granule cells is illustrated in Fig. 1D [24], where we show a basic microcircuit schematically represented with two mitral (M1 and M2 in Fig. 1D) and granule cells (GC1 and GC2 in Fig. 1D) and their dendrodendritic reciprocal synapses. When odor concentration is sufficient to activate a mitral cell tuft, an action potential (AP) is initiated locally or in the initial axonal segment and backpropagates into the lateral dendrites, activating excitatory synapses onto granule cell dendrites along the way. This is consistent with the experimental evidence that an AP can propagate throughout the extent of the lateral dendrite [25]. The activation of one or more granule cells close to the soma of a mitral cell (e.g. GC2 and M2 in Fig. 1D) by a backpropagating AP is a crucial mechanism to obtain a distance-independent lateral inhibitory action [24], as far as the AP is able to backpropagate along a mitral cell lateral dendrite.

The interconnectivity between mitral and granule cells is the critical factor in shaping the network of interactions. Experiments show that a pseudovirus tracer injected into a group of mitral cells labels a sparse and discontinuous mosaic of columns of granule cells [10]. Experimental evidence (reviewed in [26]) suggests that the overall effective connectivity is modulated by the past activity of the olfactory bulb. This process presumably builds up on an initial configuration of mitral-granule connections formed during development that is unknown. Consistent with experimental estimation of connectivity between pyramidal cells and interneurons in the cortex [27], we have chosen to use an initial configuration in which each granule cell was randomly connected to 10±5% of the mitral cell lateral dendrites directly above it, as schematically represented in Fig. 1E. There are thus approximately 0.5 million synapses in the network. Note that this does not represent the actual viral tracing data, which showed the connectivity in terms of widely distributed clusters of granule cells activated by widely distributed clusters of mitral cells, formed during the lifetime of the animal. Instead, it should be considered as the maximum average connectivity that can be obtained between any mitral and any granule cells in our network. During odor presentation, each synaptic weight will independently follow the synaptic plasticity rule to increase/decrease its value, according to the local spiking activity shaping the actual network connectivity. In a smaller network with all-to-all connectivity [18] we have previously shown how this process, through the interaction among synthetic odor inputs, action potential backpropagation, and dendrodendritic synapses can generate the kind of distributed interconnectivity observed experimentally [10]. This self-organization process can be summarized in the following way: *a*) a strong odor input generates mitral cells firing at high frequency; *b*) somatic APs backpropagate along the lateral dendrites and potentiate excitatory mitral-granule synapses along their way, activating granule cells; *c*) granule cells begin to fire at high frequency, potentiating inhibitory synapses on the lateral dendrites of mitral cells, *d*) inhibition from granule cells hinders AP backpropagation as it travels far from the soma, thus reducing the firing frequency of mitral and granule cells, *e*) this finally results in the selective depression of synapses distant from the active mitral cell soma but not those close to the soma. This mechanism is robust and independent of the plasticity rule used to update the synaptic weights during a simulation [18], [28].

With the network constructed and represented in this way, it was possible to generate a movie for each simulation to visualize the evolution of the whole network activity during an odor presentation. For this purpose, the cell somas were laid out using (X,Y) coordinates reflecting the spatial location of the real glomeruli in the dorsal surface shown by the experiments, and distributed inside a 3D space schematically representing their spatial organization in the real olfactory bulb (*initial arrangement* panel); an opened-out view of the system was used as the view point to display somatic spikes during a simulation (*opened-up view* panel). Initial snapshots from a simulation are illustrated in Fig. 1F. A low resolution movie for one of the odors (k3-3, an aliphatic ketone) is reported as movie S1, whereas a full HD version is available for public download on the ModelDB database (acc.n. 144570).

### Emergence of odor processing properties: mitral-granule cell network self-organization induced by the experimentally observed activity from 72 odors in 74 glomeruli

**Figure 2:**
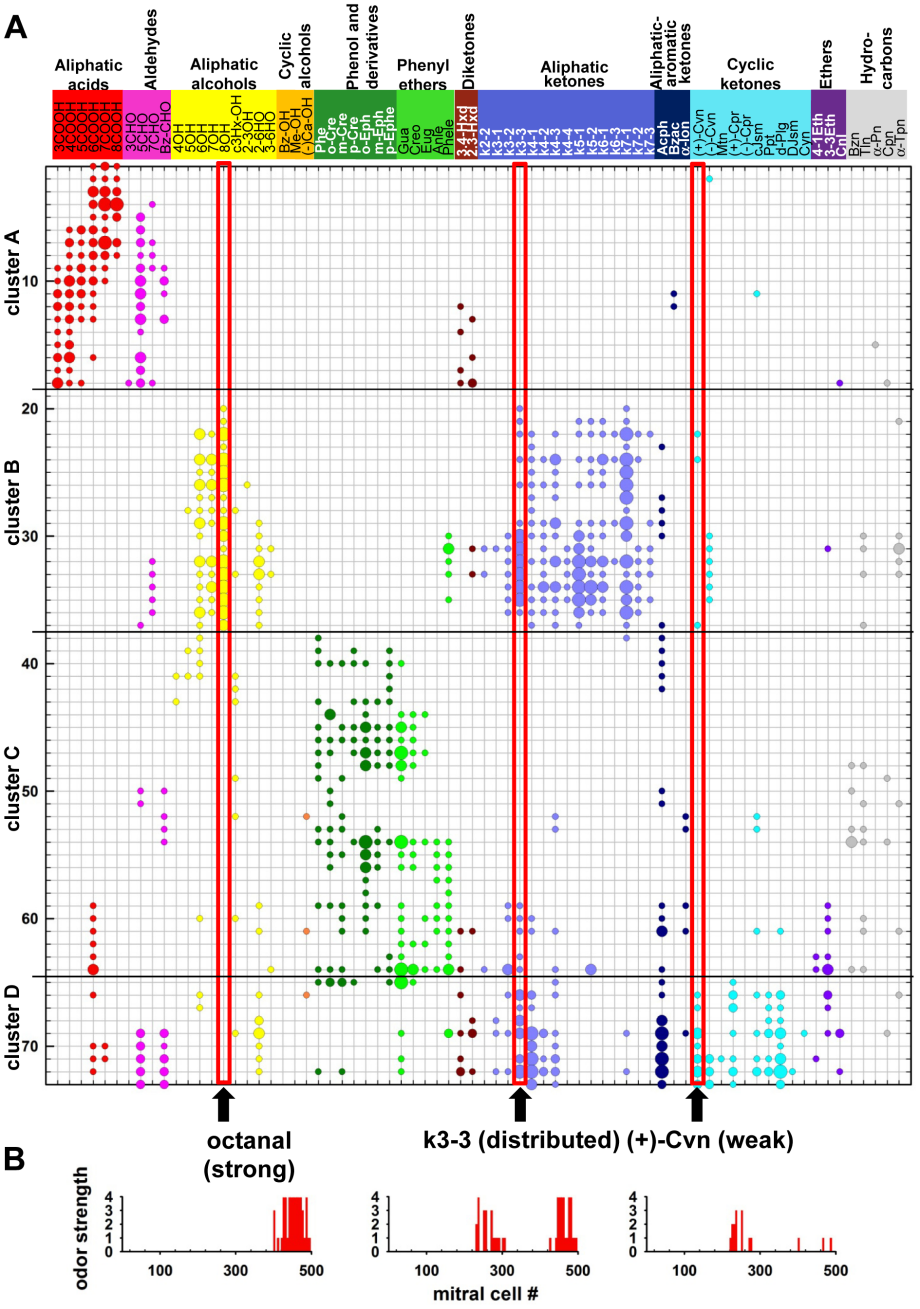
Odor inputs used for all simulations. **A**) Odor input to each mitral cell was implemented following the experimental findings [15] for 73 individual glomeruli within 4 clusters (Y-axis) during presentation of 72 different odors (X-axis). The intrinsic image responses of glomeruli in clusters A, B, C, and D in the dorsal surface of the olfactory bulb were classified into 4 response levels (weak, moderate, strong, very strong), represented in the table with circles of different sizes. In our model, the 4 response levels defined the peak synaptic excitatory conductances in the distal tuft of the mitral cells activated by each odor. The arrows at the bottom of the table point to the three odors (#14, octanal; #29, guaiacol; and #54, (+)-Cvn) that are discussed in detail in the main text. **B**) Histograms represent the input signal to each mitral cell for the three odors indicated by the arrows in part A.

In the experiments, 72 different odor molecules were used for stimulation. Individual glomeruli in each of the clusters illustrated in Fig. 1B were differentially activated by these odors, as shown in the table of Fig. 2. Each odor induced activity in a group of glomeruli, usually belonging to the same cluster but often with outliers. Mori et al. (2006) [15] classified these responses into 4 different intensity levels: very strong, strong, moderate and weak, represented by the different size circles in their table. Since it can be assumed that the intensities reflected the postsynaptic dendritic depolarization of the mitral cell glomerular tufts [29], in our model we used the response levels to set 4 different levels for the peak synaptic excitatory conductance activated in the glomerular tufts. The aggregate activation of many OSN inputs onto a given tuft with a single EPSP was implemented using a double exponential conductance change with 20 and 200 ms for rise and decay time, respectively [18], [30]. To represent the range of intensities with adequate sensitivity down to the weakest concentration without saturating the network at the highest concentration, we set the peak conductance sensitivity to give suprathreshold responses to levels 3 and 4.

We simulated each of the 72 odor responses [15] in Fig. 2 in order to analyze and compare the network responses. Key aspects of the network properties are illustrated by three examples, highlighted in the table in Fig. 2: a relatively strong glomerular response (to octanal, in cluster B), a relatively widely distributed odor response (to k3-3, in clusters B and C-D), and a relatively weak response (to (+)-Cvn, in clusters B and D). Histograms in Fig. 2B show the relative strengths and distribution of the glomerular responses, which are the input magnitudes used to activate the mitral cells.

**Figure 3:**
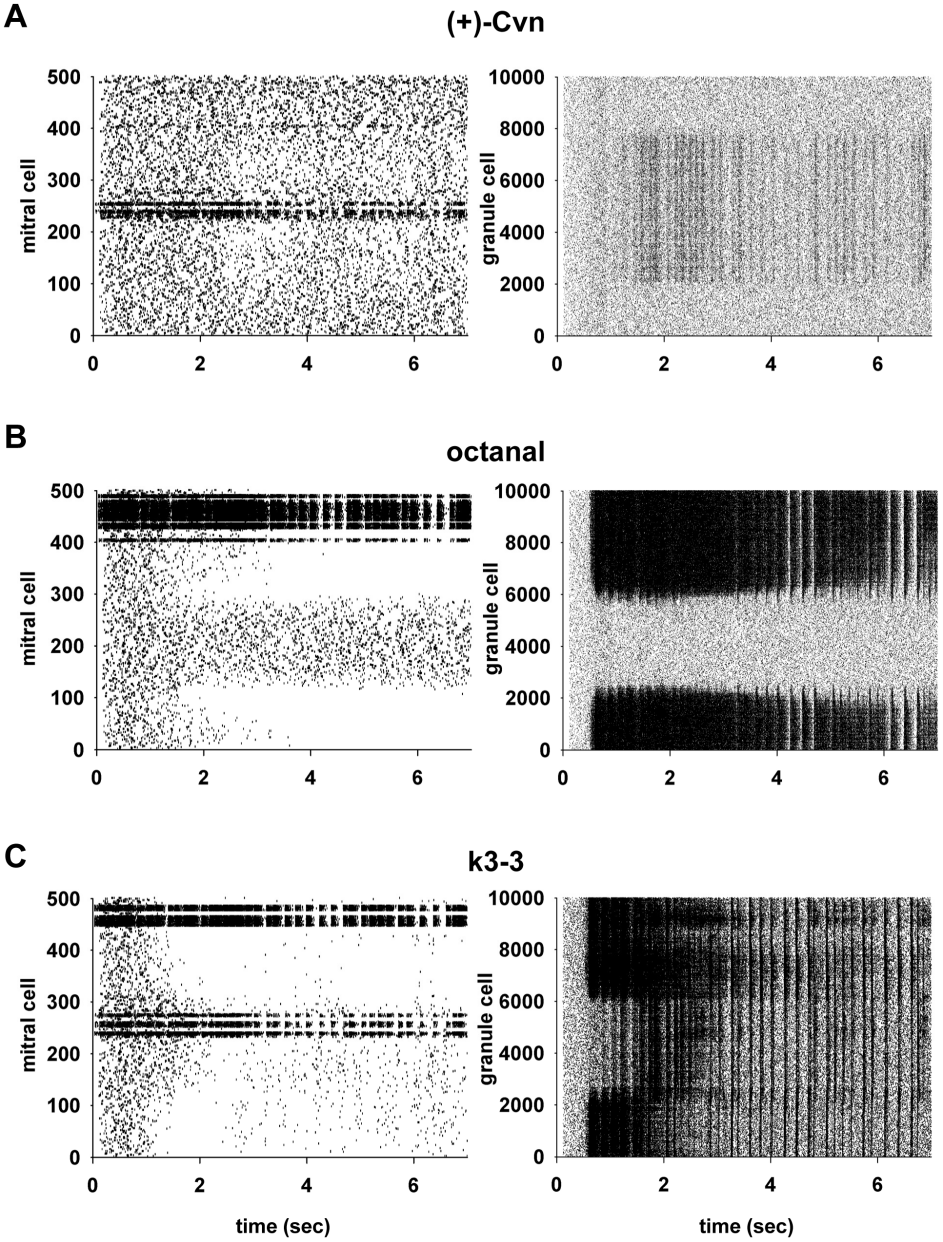
Spatial-temporal firing patterns of the large scale simulated network for three types of odor input. **A**) Raster plot of mitral and granule cell spikes during the first 7 sec of a weak odor presentation (odor 54, (+)-Cvn); note the weak suppression of mitral cell firing surrounding the most active cells after the first few seconds of simulation (*left panel*), corresponding to the increase in firing of granule cells (*right panel*) around the most active mitral cells. **B**) Raster plot for mitral and granule cell spikes during the first 7 sec of an odor with strong glomerular activation (odor 14, octanal); note a strong suppression of mitral cell firing surrounding the most active cells (*left panel*) after the first two seconds of simulation, corresponding to the increase in firing of granule cells (*right panel*) around the most active mitral cells. **C**) Simulation findings for an odor with strong and spatially distributed glomerular activation, odor 39, k3-3; note a strong and widespread suppression of mitral cell firing surrounding the most active cells (*left panel*) after the first two seconds of simulation, corresponding to the increase in firing of granule cells (*right panel*) around the most active mitral cells.

The network self-organization in these representative cases is illustrated in Fig. 3, where we show the raster plots for mitral and granule cell spike discharges during the first seven seconds of odor presentation for three odors. During a moderate glomerular input (e.g. Fig. 3A, odor (+)-Cvn), the spiking response dynamics of the most active mitral cells (around site 240) showed the progressive appearance of a bursting pattern, accompanied by weak inhibition of surrounding mitral cells to an extent of approximately 1.5 mm on either side of site 240 (note the lighter area in the mitral cells raster plot after the first 2 sec of simulation), reflecting the extent of the lateral dendrites of the activated mitral cells. Bursts were aligned with odor input activation (sniffs), with most spikes at the onset of the sniff and fewer spikes later in the sniff cycle.

The mitral cell inhibition was correlated with activation of the granule to mitral cell inhibitory synapses, as shown by the firing of granule cells in Fig. 3A (right panel), again reflecting the 1.5 mm extent of the mitral cell lateral dendrites. This granule cell spiking produced the mitral cell inhibition, as evidenced by the similarity between the granule cell spiking population and the extent of the mitral cell inhibition in Fig. 3A.

These results demonstrate several basic properties of the network response to a glomerular activity pattern. As expected, the odor drives the mitral cells receiving direct input from the activated glomeruli. This defines the mitral cell cluster related to the activated glomerular cluster. A new property shown by this realistic simulation is that the action potentials in the lateral dendrites of the activated mitral cells bring about synaptic excitation of the connected granule cells, which elicits spiking in these cells. The sharp cutoff of the granule cell spiking at 1.5 mm on either side of site 240 provides a novel indication of precisely the extents of the lateral dendrites of the driven mitral cells. This spatial pattern may be considered a 1-dimensional representation of 2-dimensional lateral, or surround, inhibition. The surround inhibition in this case is relatively weak because of the relatively weak input and the consequent weak granule cells activity. These results thus indicate an unexpectedly extensive engagement of the granule cells in the dorsal olfactory bulb area by activation of just a few glomeruli. As we will discuss later, this is not in contrast with the discontinuous mosaic of clusters of mitral-granule cell “glomerular units” shown by tracing experiments [10] but, rather, it suggests possible constraints on the transsynaptic virus transport mechanisms.

Very strong localized odor activation produced much stronger network responses. As shown for octanal, in Fig. 3B, the activated mitral cells (sites 430–490) developed an intense intermittent bursting pattern. In comparison with (+)-Cvn, the lateral inhibition was much stronger, as shown by the complete lack of spikes in the surrounding mitral cells. This strong bursting and surround inhibitory activity is explained, respectively, by the intense activation of the granule cell population at the site of the activated mitral cells (through feedback inhibition) and on either side (through lateral inhibition). In this example the same properties evidenced in the moderately activated network are seen intensified. The glomerular cluster is larger and more strongly activated, leading to a larger and more intense mitral cell response. The granule cell response is correspondingly more intense and widespread, and is associated with virtually complete inhibition of surrounding mitral cells. The discharge patterns in both cases include bursting, occasionally overwhelmed by the strength of the activation.

To contrast with these examples of localized glomerular input, in Fig. 3C we illustrate a third example, odor k3-3 (an aliphatic ketone), where a less localized, more distributed pattern of glomerular activation involved the activation of two main groups of mitral cells, stronger at the 460–490 site than at the 240–270 site. This provided the opportunity to analyze the interactions of the lateral inhibition elicited by the two sites. The mitral cell discharges (Fig. 3C, left) at both of these sites showed patterns of oscillatory bursts. Because of the separation of the two sites, the lateral dendrites of the two mitral cell populations spanned the entire network, thus activating the entire granule cell population, developing relatively uniform bursting spike discharges (Fig. 3C, right). This led to strong lateral inhibition of nearly all the mitral cells not driven by the input. Note however that this inhibition was slightly weaker over the network from sites 0 to 240, reflecting the slightly weaker input to the mitral cells at the 240 site. This indicates that the inhibition of the mitral cells reflects a balance between the amount of excitation of the mitral cell lateral dendrites and the corresponding excitation of the granule cells.

Comparison of these three responses to odor stimulation thus indicates several basic properties underlying odor coding in the olfactory bulb network. The three different odors are initially represented by the three different, spatially restricted, distributions of activated glomeruli. These spatial patterns are processed by the more extensive spatial distributions of lateral inhibition brought about by the interactions with broadly tuned granule cells. The result is a mitral cell output reflecting the spatial distribution of the glomerular input and the temporal structure of the inhibitory processing, which together represent a unique spatiotemporal representation for each odor.

### Lateral and feedback inhibition after odor presentation

**Figure 4:**
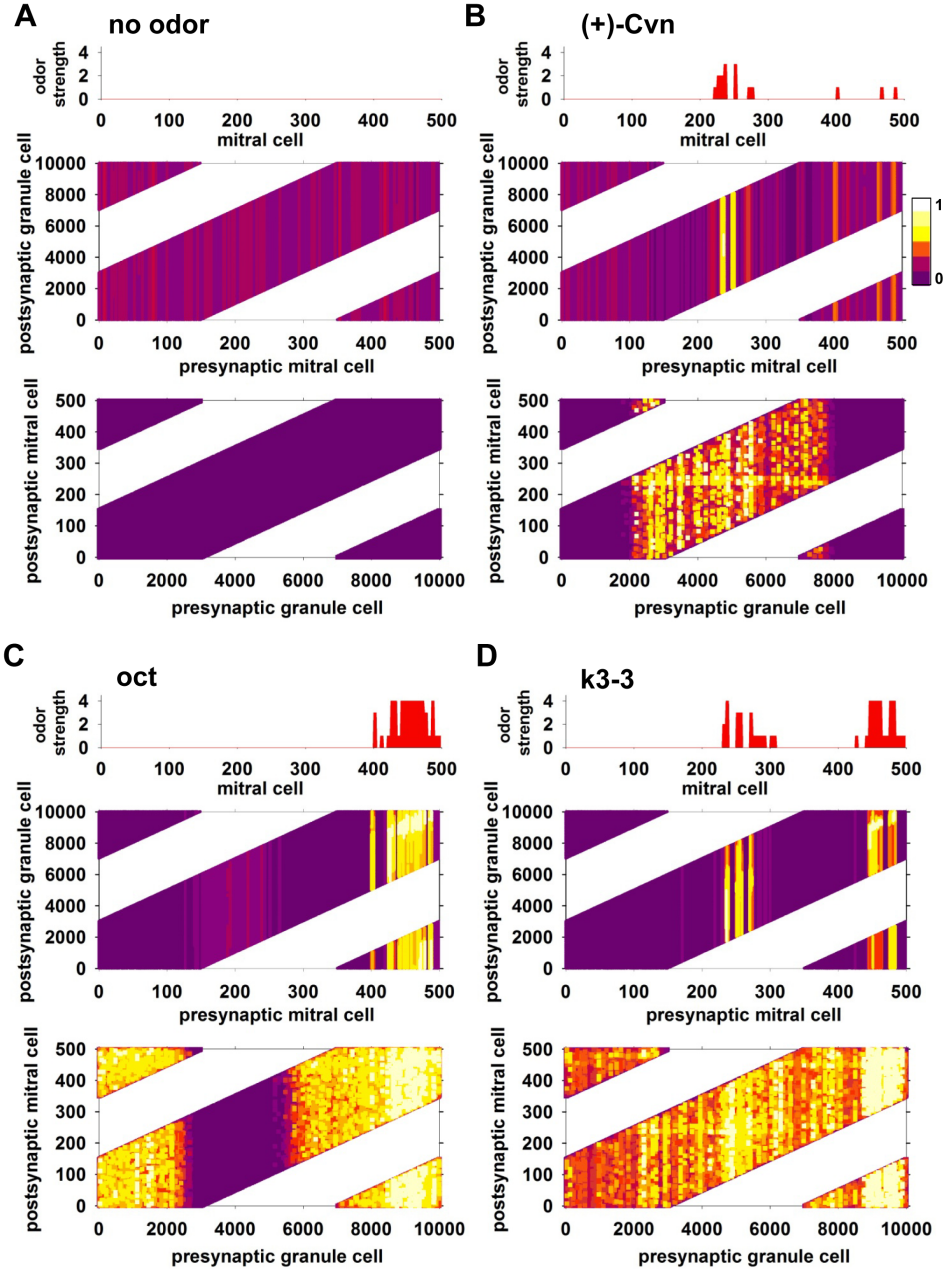
Development of synaptic weights among mitral and granule cells underlying the spike responses. In all panels, the *top* histogram represents the input strength of each mitral cell, the *middle* and *bottom* plots represent the normalized excitatory and inhibitory peak synaptic conductance after 10 sec of odor presentation, respectively; (dark purple: 0, white: 1). **A**) Without odor input. **B**) Results for odor 54, (+)-Cvn; note the extent of mitral-to-granule cell potentiated synapses in response to the glomerular input, due to action potential propagation in the lateral dendrites, and the consequent formation of distributed inhibitory columns of granule-to-mitral synapses. **C**) Results for odor 14 (octanal); note the greater extent of mitral-to-granule synapses in response to the glomerular input, caused by the larger group of activated glomeruli; **D**) Results for odor 39 (k3-3); note the spatial distribution of excitatory mitral-to-granule synapses in response to the distributed glomerular input, and the consequent wide distribution of inhibitory granule-to-mitral synapses. See also Fig. S1.

The spiking activity shown in Fig. 3 provides the driving force for the formation of the synaptic conductances that represent the odor training of the network in laying down the neural substrate for an odor perception [18]. The spiking activity in turn is driven by the weights of the tuft and dendrodendritic synapses. In order to visualize and analyze the distribution of the synaptic weights, we arranged plots of the mitral to granule cell excitatory weights and granule to mitral inhibitory weights as shown in Fig. 4. Results for all 72 odors are reported in Fig. S1.

In each panel, the top histogram shows the relative strengths and distribution of the glomerular responses. The middle graph shows the final weight configuration, after a 10 sec simulation of odor input, of the excitatory synapses from mitral to granule cells, with the 500 mitral cells on the abscissa and the 10,000 granule cells on the ordinate. The background control is illustrated in Fig. 4A, in which there is no odor input; weights of mitral to granule cell (excitatory) synapses show only random low values of peak conductance, generated by the background input (Fig. 4A, middle); this activity is not enough to generate any potentiation of the inhibitory synapses (Fig. 4A, bottom), which stay at their initial value of zero.

The moderately intense focal glomerular activation by (+)-Cvn (Fig. 4B, top) at mitral cell sites 240–250 elicited action potentials that propagated through their secondary dendrites. This activated the excitatory conductances of the mitral to granule cell synapses between granule cell sites 2,000–8,000 (Fig. 4B, middle, yellow areas). Note the slight increase in excitatory weights at mitral cell sites 400–590; this reflects the summation of the very weak glomerular input with the background activity). This excitatory activity in turn activated granule cells and, thus, potentiation of granule-to-mitral cell inhibitory synaptic conductances (Fig. 4B, bottom). Yellow to white pseudocolors represent synaptic weights fully potentiated, and in this case they are distributed throughout the extent of the most active mitral cell lateral dendrites.

Comparison with the spiking data of Fig. 3A shows a close correlation between the location and strength of the synaptic weights and the patterns of mitral and granule cell spiking. For example, the thin horizontal bands of potentiated inhibitory weights (at mitral cell sites 240–250 in the bottom graph of Fig. 4B) correspond to granule cells connected to the most active mitral cells; the strong feedback inhibition generated by their activation is responsible for the emergence of the bursting behavior observed in Fig. 3A. The analysis of synaptic weight distribution in the network for the case of a much stronger odor is shown in Fig. 4C (octanal). In this case, the wider range of potentiated excitatory synapses (Fig. 4C, middle) generated a strong and widespread potentiation of inhibitory weights (Fig. 4C, bottom), with an evident inhibition of the mitral cells surrounding the most active ones, as indicated by the darker purple area in the excitatory weights map in the range of mitral cells 280–400 and 490–120. Note that this correlates with the strong lateral inhibition shown in the left panels of Fig. 3B. For a more distributed input, such as odor k3-3 (Fig. 4D, top), the excitatory weights reflected the glomerular activation at the two sites (Fig. 4D, middle), and the consequent strong inhibitory weights on the most active mitral cells (Fig. 4D, bottom). In this case, the distributed input involved more or less synaptic plasticity of the entire network of inhibitory weights, with the flanking weights reflecting the overlap of mitral cell lateral dendrites, for example granule cells in the range around sites 2300, 6200, and 8000. The overall effect on network activity was a widespread bursting behavior that involved the entire granule cell network (see Fig. 3C, right). The formation of different excitatory and inhibitory clusters in response to all odors is shown in Fig. S1.

In summary, these results demonstrated how the learning of different odors can generate, through the differential activation of distributed glomeruli, widely different network behavior with: *i*) distinct firing properties involving a variable population of granule cells, *ii*) an emergent oscillatory bursting behavior that can span a large portion of the olfactory bulb, and *iii*) a powerful lateral inhibition surrounding the most active glomeruli.

### Inhibitory conductance as a function of input strength

**Figure 5:**
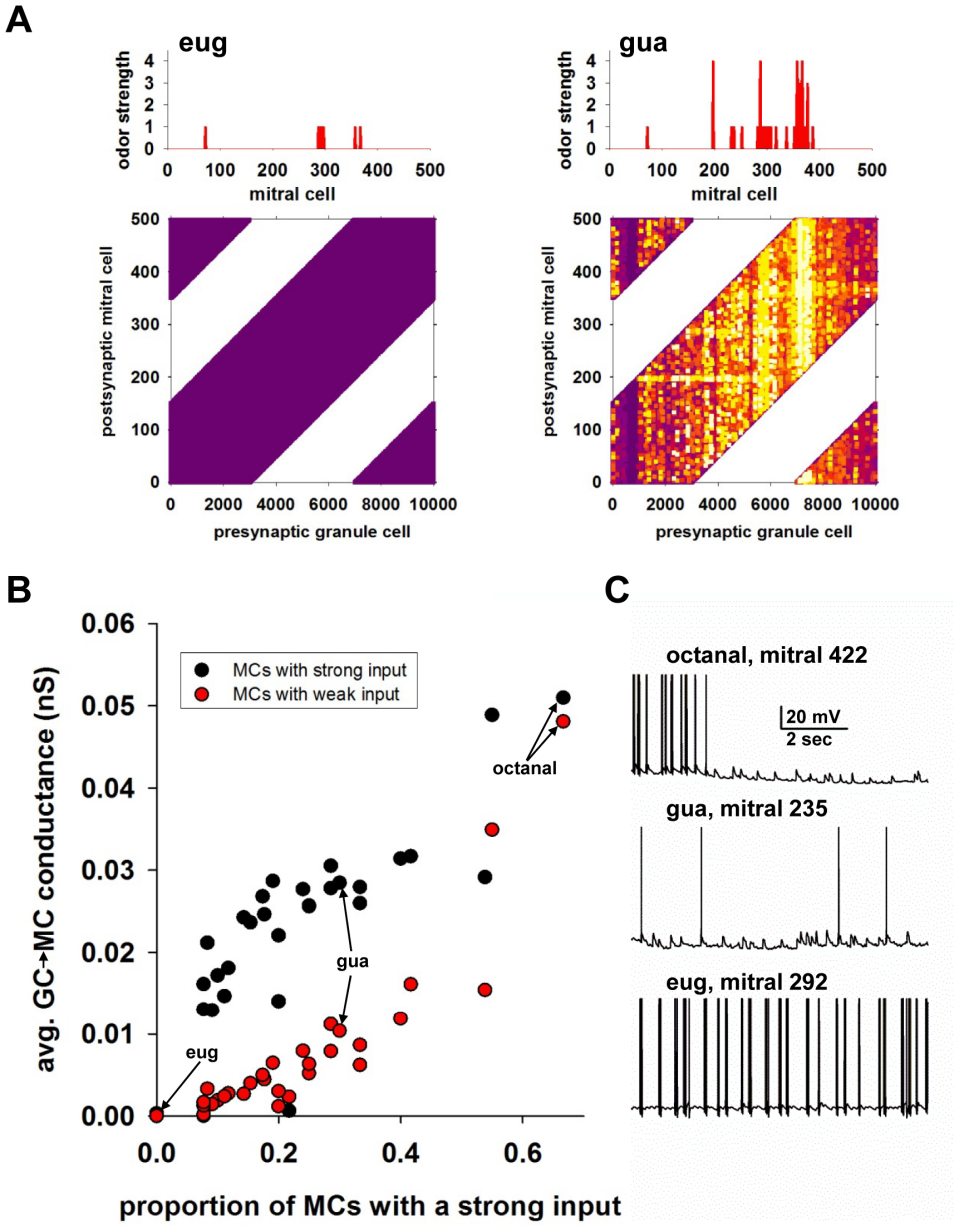
Odors with a strong input generate lateral inhibition on weaker components. **A**) Two typical odors with different input structure; top panels represent the input to each mitral cell; bottom panels show the distribution of the normalized peak inhibitory (granule-to-mitral) conductance after odor learning (dark purple: 0, white: 1); (*left*) example of a weak odor, all activated mitral cells receive weak input; (*right*) example of a distributed odor, active mitral cells are spatially distributed and receive both weak and strong inputs. **B**) Average granule-to-mitral peak inhibitory conductance for each odor; in all cases the average peak conductance was calculated over the set of mitral cells receiving a weak input (input levels 1–2, red circles), or a strong input (input levels 3–4, black circles); note that a value of 0 for the peak inhibitory conductance would be expected from a weak input. **C**) Typical traces from different mitral cells, all receiving a weak input from three odors (octanal, gua, and eug), during 10 sec simulation of odor learning; note the powerful inhibition, from mitral cells activated by odors with a very strong component (e.g. octanal, see Fig. 4C) after the first few seconds of simulation, corresponding to the initial organization of the network.

The relation between lateral inhibition and glomerular activation is critical to odor representation and processing. To understand this property in a more quantitative way, for each odor we calculated the average inhibitory conductance on any given mitral cell as a function of the input strength. We were particularly interested in mitral cells receiving a weak input (i.e. an odor strength of 1 or 2). For any given odor, these cells correspond to flanking components. Typical cases can be identified in the histograms representing odor input for each odor, for example mitral cell 292 for Eug (Fig. 5A, left), mitral cell 422 for octanal (Fig. 4C, top), and mitral cell 235 for Gua (Fig. 5A, right). They all receive a weak input, but the input of their respective neighbors is quite different, as can be seen in the different histograms for these odors: weak neighbors for Eug, very strong for octanal, and medium for Gua. Given the low connection probability (10%) and the spatially distributed glomerular activation, the emergence of a significant lateral inhibition cannot be taken for granted, and it would be difficult to explore experimentally.

In order to analyze the relation between input strength and the effect of the lateral inhibition it generates, mitral cells activated by any given odor were grouped in two input classes, low (strength 1–2) and strong (strength 3–4). For each odor and each group, the average overall inhibitory conductance was then calculated from the final weight configuration at the end of the 10 sec simulation, including both feedback and lateral actions. The results are shown in Fig. 5B, as a function of the proportion of cells receiving a strong input. Note that without any lateral inhibition the peak inhibitory conductance on mitral cells receiving a weak input (Fig. 5B, red circles) would be 0, since their firing rate would be too low to generate any feedback inhibition. However, the results show that lateral inhibition is developed as an odor activates a small proportion of mitral cells with a strong input.

The differential effect on the weaker flanking components can be clearly seen in Fig. 5C, where we show the somatic membrane potential during the first few seconds of a simulation for three mitral cells. As the weights develop, a strong odor, such as octanal, will completely silence a flanking component, such as cell 422 (Fig. 5C, top), whereas a progressively lower effect can be seen for cell 235 during presentation of the less strong Gua (Fig. 5C, middle), and for cell 292 during presentation of the weak Eug (Fig. 5C, bottom). These results demonstrate that significant lateral inhibition can be developed by any given odor that is able to generate strong activity in a relatively small proportion of mitral cells, independently of their spatial location (but within the reach of their lateral dendrites).

### Effects of a sparse granule-mitral cell connectivity

**Figure 6:**
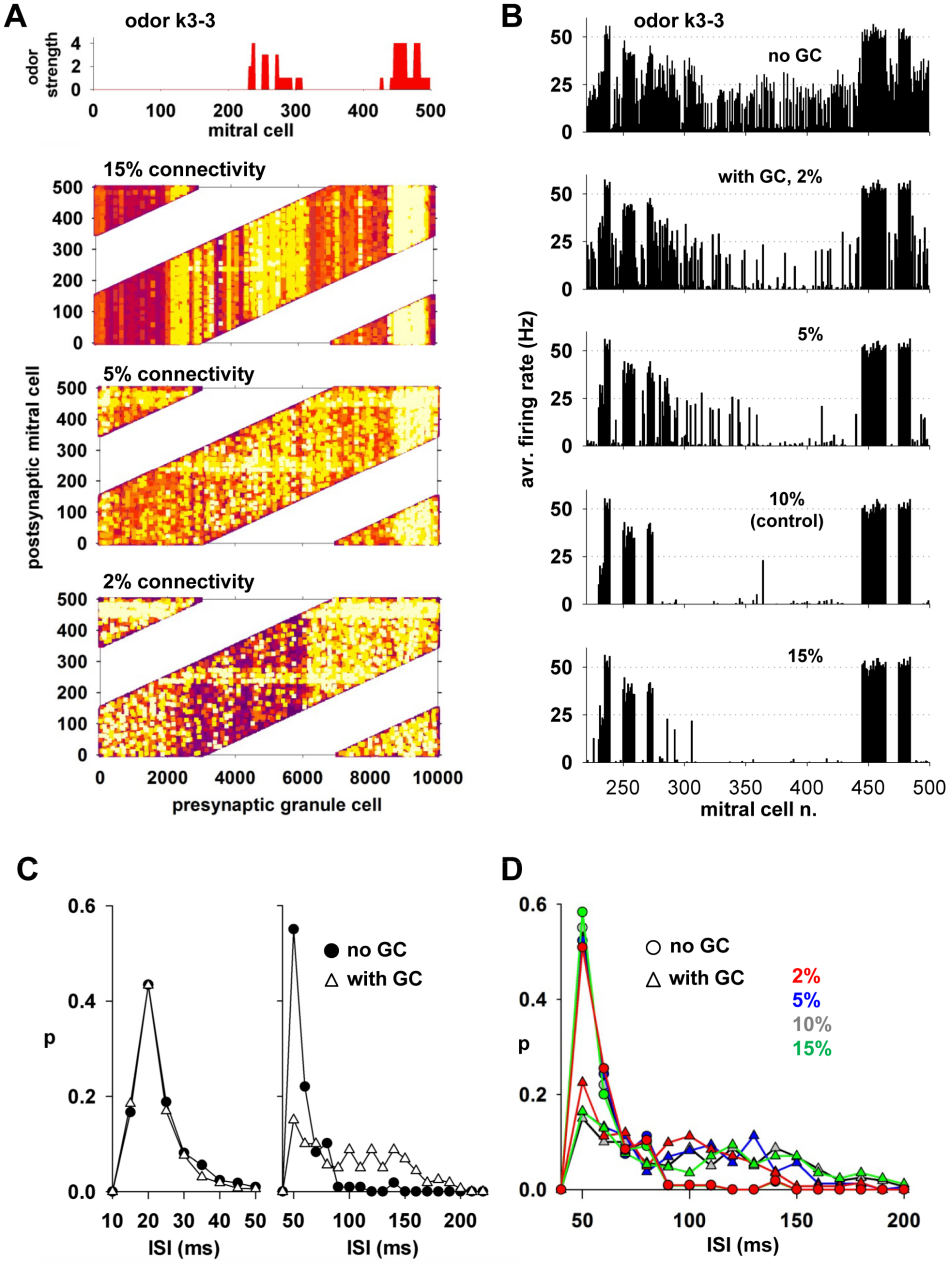
Sparse granule-mitral cell connectivity modulates in a robust way a mitral cell response to an odor. **A**) Inhibitory synaptic weights (granule-to-mitral) after 10 sec presentation of odor k3-3 using different levels of granule-mitral connection probability; the *top* histogram represents the input strength on each mitral cell. **B**) Average instantaneous firing rate of mitral cells 220–500 during odor k3-3 presentation, without granule cells (*no GC*) and with GCs connected to mitral cells with different probabilities (*2-5-10-15%*). **C**) Interspike interval (ISI) distribution of the most active mitral cells (odor strength>2) in the range 0–50 (*left*) and 50–200 ms (*right*) with (*triangles*) or without (*circles*) GCs with 10% connectivity; **D**) ISI in the range 50–200 ms with (*triangles*) or without (*circles*) GCs using different connection probabilities with mitral cells. In all cases, the first and the last second of 10 sec simulations were used to calculate firing rates and ISI probabilities without or with GCs, respectively.

The olfactory code relayed from mitral cells to the cortex for odor recognition is sculpted by the activity of granule cells, which are ideally positioned for this role in the olfactory bulb circuit. The granule-mitral cell connection probability can thus be expected to have a paramount role in modulating mitral cell firing. Experimentally, the average probability with which a granule cell forms synapses with mitral cells is unknown, although there are findings suggesting that, in general, it is sparse and spatially distributed (see Discussion). To test to what extent a sparse connectivity can significantly modulate mitral cell firing we carried out additional simulations for odor k3-3 using different connection probabilities between granule and mitral cells (Fig. 6). The configuration of inhibitory weights after a 10 sec simulation using an average maximum potential connectivity of 2, 5, and 15% is shown in Fig. 6A. In comparing the results obtained with the control value of 10% (Fig. 4D), with those obtained using a higher connectivity (Fig. 6A, 15%), we observed a sharp difference in the clustering of high synaptic weights around the two regions with active mitral cells with respect to those in other regions. This difference tended to be smaller with 5% connection probabilities and almost disappeared with 2% connectivity.

We hypothesized that these differences may result in a significant change of mitral cell firing properties. We tested this in the model by analyzing the average instantaneous firing rate, which is the most relevant parameter to characterize and understand mitral cell responses to an odor sniff. Without GC all mitral cells were more or less active, depending on background activity and/or odor input (Fig. 6B, top). The background activity of mitral cells not receiving any odor input (e.g. cells in the 325–425 range) was much reduced already with only 2% connectivity, and almost completely suppressed with connection probabilities above 5%. A higher connection probability (10 and 15%) also suppressed firing of flanking components, a typical contrast enhancement effect. Most interestingly, the firing rate of strongly activated mitral cells was little affected by connection probability. This was more clearly evident from the analysis of their ISI distribution in the range of 10–50 ms, as shown in Fig. 6C (left) for 10% connection probability. The two distributions (with or without granule cells in the network) were statistically indistinguishable (Mann-Whitney Rank Sum Test, *p* = 0.88), in contrast with the distributions of 50–200 ms ISIs (Fig. 6C, right, Mann-Whitney Rank Sum Test *p* = 0.021). This effect was very robust with changes in connection probability (Fig. 6D). The distributions of 50–200 ms ISIs with or without GC in the network were significantly different in all cases except 2% connectivity (Mann-Whitney Rank Sum Test *p* = 0.115).

These model results predict that a sparse granule-mitral connectivity will be able to significantly affect mitral cell firing in two different ways: 1) suppressing flanking or weaker components, and 2) changing the firing pattern structure of the stronger components. The overall computational effects for odor coding of these mechanism will be investigated in a future work.

### Complementary role for mitral cell feedback and lateral inhibition

**Figure 7:**
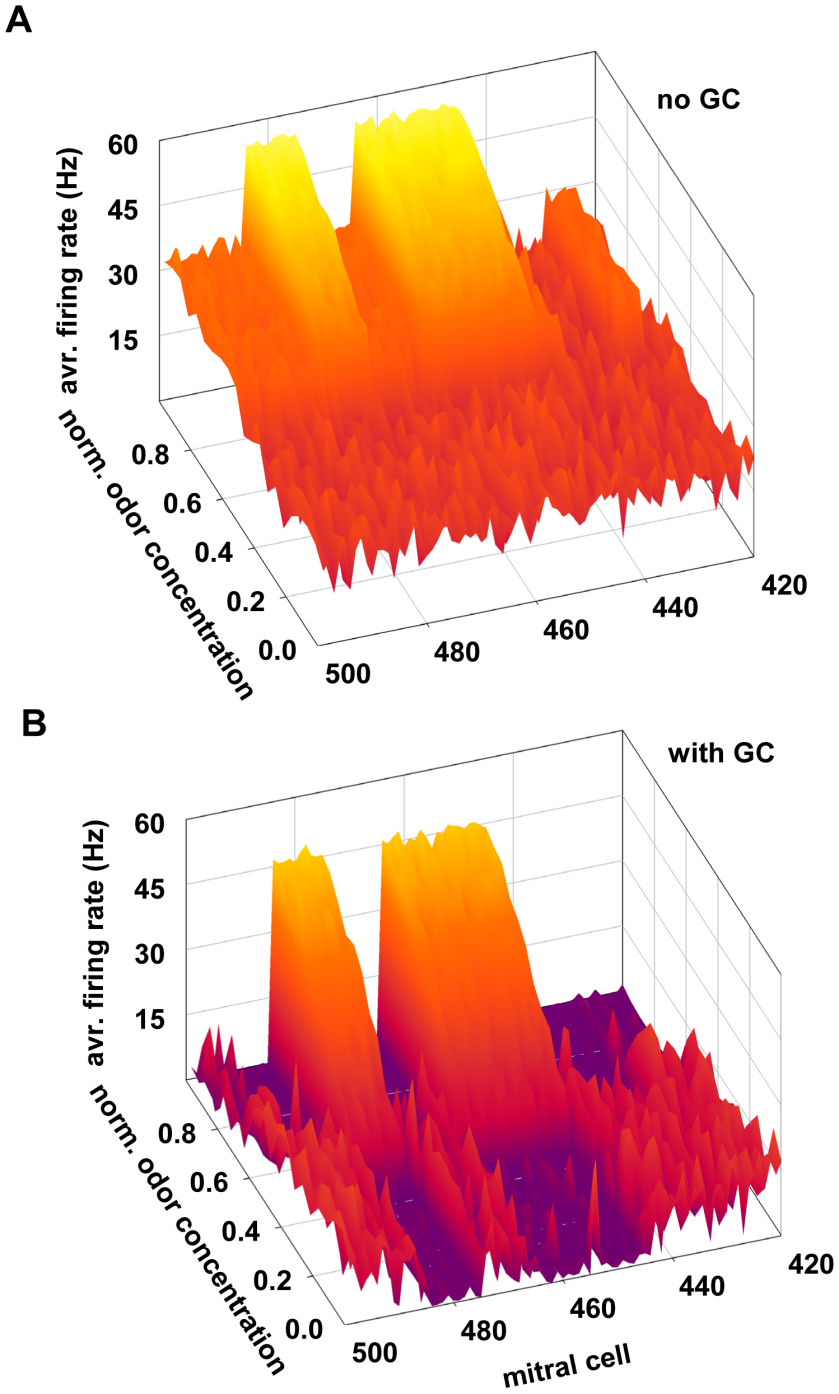
A complementary role for mitral cell feedback and lateral inhibition. **A**) Average instantaneous firing rate of mitral cells 420–500 during 5 sec presentation of odor k3-3 at different concentrations without granule cells in the network; note the rather uniform and unstructured mitral cell activity at low odor concentrations (below 0.5). In all simulations, synaptic plasticity was blocked and a synaptic weights configuration corresponding to the “no odor” condition (see Fig. 4A) was used in all cases. **B**) Average instantaneous firing rate as in A) but with granule cells in the network; in all simulations synaptic plasticity was blocked. The synaptic weights configuration obtained after presentation of odor k3-3 (see Fig. 4D) was used in all cases. Note the firing depression of mitral cells activated by low odor concentrations (odor concentration below 0.5, mitral cells 446–465 and 476–485), and the firing depression of flanking mitral cells for higher odor concentrations (odor strength above 0.5, mitral cells 420–445, 466–475, and 486–500).

The relative importance of the feedback and lateral inhibition generated by a sparsely connected network of granule cells is poorly understood. We have shown how they can modulate the overall mitral cell firing structure by different odors (Fig. 3) and under different connectivity (Fig. 6). To test their effects on different odor concentrations we carried out, without granule cell synapses in the network, a 50 sec long simulation of odor k3-3. Odor strength was progressively increased every 5 sec, from 0 (no odor) to 1 (the maximum strength used in all simulations) in steps of 0.1. The results, shown in Fig. 7 (top) for mitral cells 420–500, show a rather high and diffuse activity of all mitral cells, with those receiving relatively lower inputs (<0.4) practically indistinguishable from the background activity (average S/N = 0.25 dB). We then repeated the simulation including granule cells and starting from the weights configuration obtained after odor learning (Fig. 4D). The results, illustrated in Fig. 7, showed a surprising effect of feedback inhibition for low odor concentrations, which selectively suppressed the instantaneous firing activity of the most active mitral cells (e.g. cells 475–484), making them clearly distinguishable (average S/N = −13.1 dB) from neighbor cells activated by the background noise (e.g. cells 430–439) or by weaker components (486–500). These results suggest that lateral and feedback inhibition work together, in a complementary way, to enhance the contrast between an odor signal and the background noise over a wide range of odor concentrations, including subthreshold inputs.

### Comparison with experimental findings

**Figure 8:**
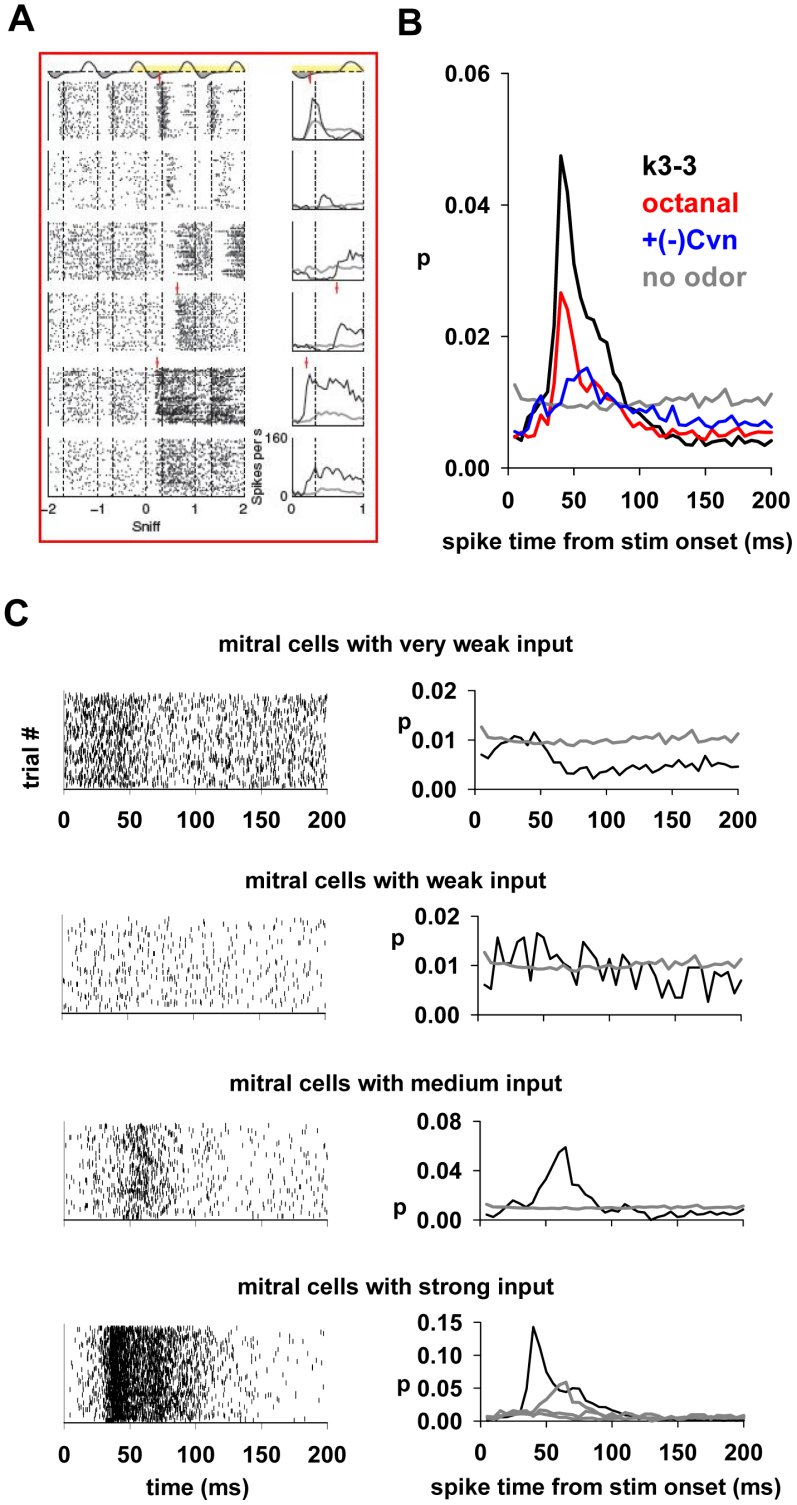
Spike timing of individual mitral cells following odor onset depends on input strength. **A**) Experimental findings (from [16], Reprinted by permission from Macmillan Publishers Ltd: Nat. Neurosci.) on the spike times of 6 mitral cells during presentation of the same odor, warped to the sniff cycle (*left*) and their post stimulus time histograms (PSTH) (*right*). **B**) Distribution of spike times from the stimulus onset in our model for octanal (strong odor, *red*), k3-3 (strong and distributed, *black*) and +(−)Cvn (weak, *blue*); light grey line represents the distribution of spike times from background activity. **C**) (*left*) raster plots of spike times from 70 simulated sniffs for mitral cells activated by odor k3-3, grouped according to the 4 input levels coding odor strength in the different glomeruli (see Fig. 2); (*right*) corresponding distribution of spike times from the stimulus onset. The gray line in the top three graphs represents the “no odor” condition; to aid in comparing the distribution of mitral cells with strong inputs (bottom graph) with those obtained under other conditions, the distributions for “no odor”, medium, and weak inputs are indicated by gray lines.

We wished to test the network model against physiological recordings of mitral cells responding to odor stimuli. We have previously shown that a reduced network model gives generic mitral cell responses consistent with those reported for different odors in a homologous series [7]. A more detailed, although qualitative, comparison with the present model can be carried out with the data reported in [16]. These authors found that the temporal firing structure of mitral cells in response to a sniff cycle is very precise, with different mitral cells responding with different onset times and firing rate to the same odor (Fig. 8A).

We were particularly interested in understanding the reasons for the temporal firing distribution of the response with respect to a sniff cycle. The experimental findings (Fig. 6A, see [16]) are quite clear from this point of view: Different cells respond to the same odor with different timing. This is increasingly recognized as an important computational property for odor coding and discrimination (e.g. [1], [31], [32]), but the underlying processes are unknown.

In order to investigate this issue, which is otherwise experimentally limited within a network framework, we calculated the average distribution of spike times of all the mitral cells activated during 70 sniffs by a strong, a medium, and a weak odor. As shown in Fig. 8B, the distributions were different, and all were clearly distinguishable from the “no odor” condition (Fig. 8B, gray line). A more specific analysis of mitral cells activated by k3-3, obtained by grouping the mitral cells according to the strength of their input (Fig. 8C), suggested that the different distributions are correlated with the interaction between the odor input strength and granule cell activity. Mitral cells receiving a weak or very weak input (Fig. 8C, top two panels) are mostly modulated by weak lateral inhibition generated later in the sniff cycle, whereas cells receiving medium and strong inputs (Fig. 8C, bottom two panels) generate earlier strong inhibition that impacts the response latency.

Taken together these results indicate how odor identity could emerge from a single sniff as a specific distribution of spikes composed of spatially and temporally positive or negative contributions (with respect to the “no odor” condition) from all the mitral cells activated by the odor, each mitral cell making its own contribution according to the specific type of input it receives and the underlying network of granule cells it activates.

**Figure 9:**
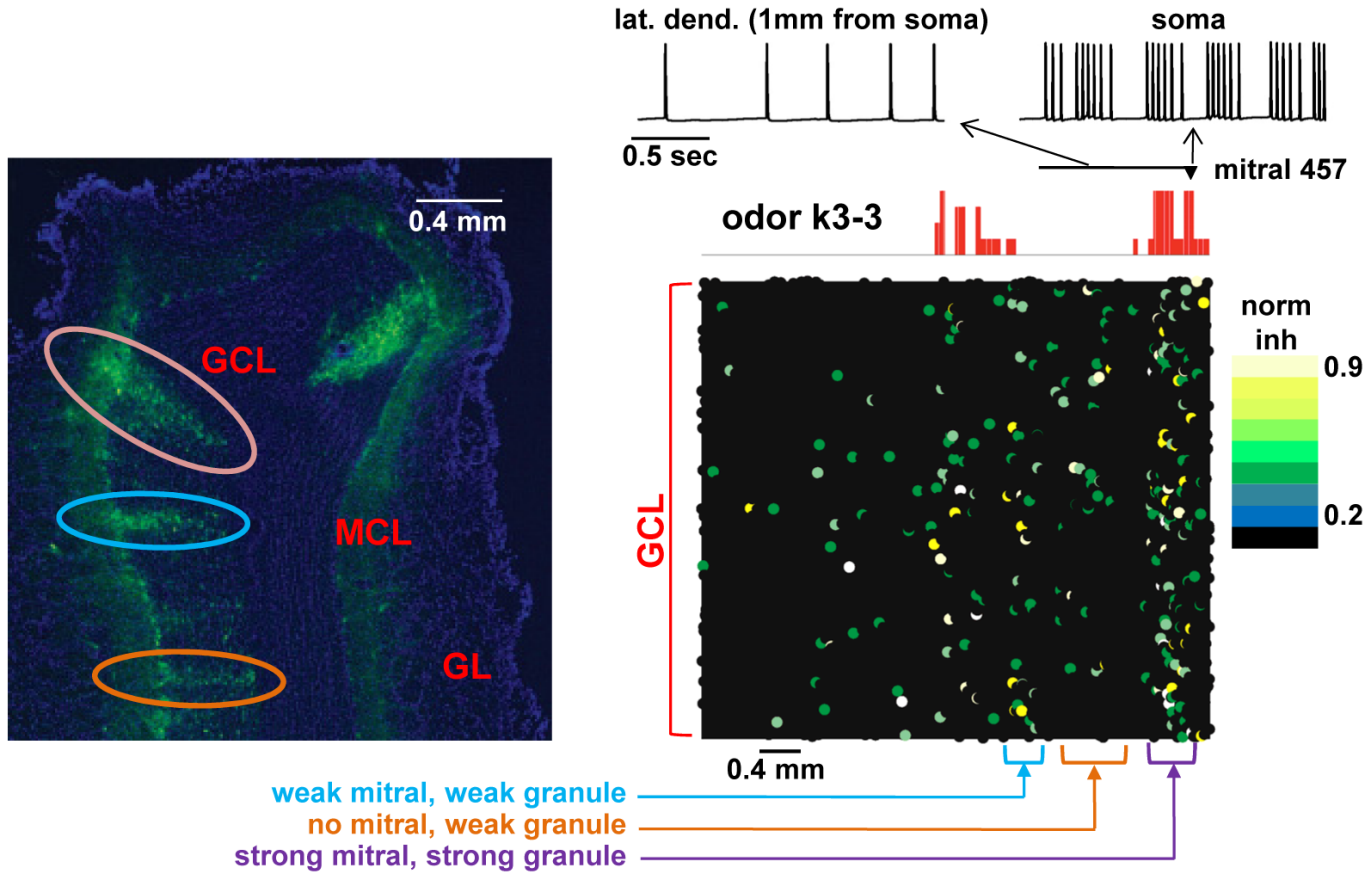
Emergence of distributed mitral-granule cell connectivity. (*Left*) Typical experimental findings for pseudorabies virus staining patterns after olfactory bulb injection (adapted from Fig. 2D of [10] with permission from the National Academy of Sciences, U.S.A); the photo shows a coronal section of the olfactory bulb, with labeling of columns of granule cells; GCL, granule cells layer; MCL, mitral cells layer; GL, glomerular layer; colored loops indicate regions of strong (purple), medium (blue), and weak (orange) cell activation. (*Right*) Model results after learning of odor k3-3. The top traces show bursts in the soma (*right*) that become single spikes as they propagate in a lateral dendrite (*left*) of mitral cell 457 at the end of the learning period (*t* = 8–9 sec). The red histogram represents the strength of glomerular input to the mitral cell tuft (see Fig. 2B), and the field shows the location and normalized peak inhibitory conductance from granule cells on the mitral cell lateral dendrites (see color bar: strong conductance in yellow; moderate in green; low or absent in black). Note that the locations of strong conductances line up primarily with sites of mitral cell responses but also occur in the intervening spaces (see text). Regions similar to the experimental patterns are indicated with different colors (purple, blue, orange).

It is also of interest to compare the model properties with recent experimental studies which revealed sparse and segregated lateral connectivity between mitral and granule cells [10], [17], [33], as illustrated in Fig. 9 (left panel). It showed the connectivity in terms of widely distributed columns of granule cells labeled by widely distributed clusters of mitral cells, formed during the lifetime of the animal. It therefore represents the “maximum” average connectivity that can be obtained between any mitral and any granule cell in the network. During odor presentation, each synaptic weight will independently follow the synaptic plasticity rule to increase/decrease its value, according to the local spiking activity, shaping the actual network connectivity. We have previously shown [18] that this process, through the interaction among odor inputs, action potential backpropagation, and dendrodendritic synapses can generate the kind of distributed interconnectivity observed experimentally [10].

We tested whether our dynamic circuit model could produce similar patterns. As a typical example, we considered all mitral cells activated by odor k3-3 and calculated the peak inhibitory synaptic conductance along their lateral dendrites after odor learning. This is equivalent to the experimental protocol used in the PRV injection experiments, where a few nearby glomeruli are injected with the virus to give labeling of the widely distributed granule cell clusters. Although it contained no inherent cluster connectivity, as shown in Fig. 9 (right), the circuit model generated narrow labeled granule cell patterns similar to those observed experimentally. This included the strong clustering of inhibitory synapses below the most active mitral cells in the model (Fig. 9, right), corresponding to a region of intense staining of mitral and granule cells in the experiments (Fig. 9, left), and the weaker clustering of granule cells in a region with no active mitral cells (Fig. 9, indicated in orange). In the top traces in Fig. 9, right, it can be seen that, associated with this pattern, the soma of strongly activated mitral cells showed strong action potential firing, whereas the invasion of distal lateral dendrites was weaker. This suggests that the granule cell inhibitory synapses, once established, can gate the backpropagation of a train of action potentials in a precise location along a mitral cell lateral dendrite as suggested experimentally (e.g. [9], [25], [34]).

These results confirm our previous suggestion [18], [26] that the sparse and distributed patterns observed experimentally can emerge from the interaction between mitral and granule cell activity during an odor presentation.

## Discussion

Many studies have documented the spatial patterns of olfactory glomeruli activated by odor stimuli, but the responses of the olfactory bulb network in processing that input are still poorly understood. A major technical problem in the experimental investigation of this issue is the practical impossibility of recording simultaneously from a large set of mitral and granule cells activated by an odor. The present scaled up model of interactions between populations of mitral and granule cells is a step toward the goal of understanding the emergent properties of the olfactory bulb network in generating its output to the olfactory cortex. Here we discuss several novel features arising from the present study that are directly related to experimental observations.

### Lateral inhibition as a mechanism for efficient odor representation

Lateral inhibition of mitral and tufted cells has been firmly established by many experiments at the single cell level over the past nearly 50 years [6]–[7], [35]–[41], providing evidence that excitation of mitral cells leads to dendrodendritic activation of the granule cells, which then brings about feedback and lateral inhibition of the mitral cells. The interpretation of these studies has been supported by a number of realistic (e.g. [24], [26]), simplified (e.g. [39], [42]), or artificial (e.g. [43]–[44]) computational models. The present study has enabled us to extend the characterization of lateral inhibition from the single cell to a realistic network level, and provided a clearer representation of the sharpening by the lateral inhibition of the neural response to different odor inputs.

We have shown that activity-dependent mechanisms are capable of sculpting the network, leading to the formation of dendrodendritic synaptic clusters in a large, sparsely-connected network. The independent evolution of each synaptic weight, according to the local dendritic spiking activity, will shape the actual network by forming the widespread mosaic of clustered connectivity observed experimentally [10]. This mechanism may be in effect, for example, during the important process of merging newborn granule cells, with their facilitated synaptic plasticity [45], into the existing bulb network to drive stimulus response decorrelation [44].

The spatial extent of the lateral inhibition was correlated with the strength of mitral cell activation; stronger glomerular activation results in more extensive lateral inhibition. Stronger antidromic activation of mitral cells has been known to be associated with more extensive lateral inhibition of surrounding mitral cells [46]. The model indicates how orthodromic mitral cell activation from the glomeruli produces the same result in the mitral cell ensemble.

In relation to recent experimental findings [16], the model explains in terms of different input strengths the results showing different timing among the mitral cells activated by an odor, and supports the suggestion that the time-to-first-spike can be a critical property for odor identification as the mitral cells project their ensemble response to olfactory cortex [31].

With the use of single odor stimulation, as in experiments discussed in [15] and the present model, the spatial pattern of inhibition reached the full extents of the lateral dendrites of the activated mitral cells, since the action potentials can propagate, in the absence of active inhibitory synapses, to the ends of the lateral dendrites. This has been experimentally demonstrated [25], although there is evidence that it may not occur under some conditions [47]. However, our model was also able to demonstrate intermittent clusters (Fig. 9). This was surprising because the initial specific connectivity rules between mitral and granule cell dendrites formed during neurogenesis are not known; we hypothesize that when those rules will be identified the clustering will be even clearer and more widespread.

Full propagation is most likely to occur under conditions of single odor stimuli, in which the odor activates specific isolated clusters, with associated mitral cells spanning regions of reduced connectivity with granule cells, as in the present study. In nature, however, odors are usually smelled as combinations of many components that may activate glomeruli in much more complicated and dense patterns [48]. This experience will thus drive mitral cells to set up correspondingly more or less complicated patterns of inhibition which will modulate and gate the backpropagating action potentials, as demonstrated experimentally [9], [25] and computationally [26]. This forms the foundation for the emergence of lateral inhibition in such a large-scale network (see Figs. 3 and 4) and provides potential functions for modulating precise spatio-temporal patterns of action potentials during exposure to different odorants.

### Spatiotemporal coding and sniffing

There are two major components of information contained in an odorous molecule: odor molecule identity and odor concentration. It is suggested that mitral cells might employ both firing rate of individual mitral cells and spatial patterns of spike timings of particular combination of active mitral cells in encoding odor molecule concentration and identity information separately [49]–[50]. Recent experiments showed that mitral cells sharing the same glomerulus have highly correlated firing rates in response to odors, but their spike timings are relatively different with respect to the phase of the sniff cycle [50]. These studies suggest that mitral cells may use different coding channels to represent information, but cannot extract more specific information on the underlying process. Our study is in full agreement with those studies, and suggests that the spatial patterns of precise timing locked to sniff cycle may represent odor identity, by generating a spatio-temporal representation of odor molecule information.

Lateral inhibition by recurrent granule cells not only plays a role in decreasing firing rates of olfactory bulb activities resulting in “spiking packets” [51], but can also enhance synchrony [30] and precise timing of both mitral cells and granule cells, suggesting a temporally sparse code. In awake animals, the act of sniffing increases the air velocity and changes the duration of airflow in the nose, which improves olfactory detection [52]. It has also been shown that the waking state [53] enhances the level of inhibition in the network, which increases the sparseness of the mitral cell responses. Our model is consistent with this result (Fig. 5 C; see also Fig. 6B), which will be explored in more depth in a future study.

Behavioral studies have suggested that there is an active process modulating neuronal responses during sniffing [52]. This may produce optimal temporal sequences in the olfactory bulb [54]. Recordings *in vivo* and *in vitro* have shown evidence that the frequencies of response oscillations are modulated by breathing and sniff rates [1], [16], [51]–[52], [54]. Our model provides one explanation by showing how lateral inhibition can modulate this process by strongly suppressing the background noise while synchronizing the mitral cell responses. A more comprehensive analysis of mitral cell responses in the presence of mixtures at different concentrations in relation to the sniff cycle will be presented in a future study.

### Sparse coding and downstream processing

Recent studies have suggested several possible mechanisms that could result in sparse odor coding in the olfactory bulb. Interestingly, there are also recent experimental findings *in vivo* [48] showing that natural odorants are represented by dense (as opposed to sparse) glomerular activation. In this paper we demonstrate that a sparse representation can emerge naturally from the mitral-granule cell interactions, realistically implemented in our model with self-organizing dendrodendritic synapses driven by mitral cell activity. Feedback and lateral inhibition cooperate to maintain a sparse representation complementarily acting over different odor concentrations. This differs from more theoretical and speculative artificial network models suggesting that sparse coding in the mammalian olfactory bulb can arise from an external cortical signal generating an incomplete feedforward inhibition [49] or from feedforward inhibition in the mushroom body of insects [55]–[57]. In regard to downstream processing, in modeling the analogous antennal lobe of the fly it has been recognized [55] that it is necessary to know how the output is processed in the downstream mushroom body and lateral horn. Those downstream mechanisms are not well known even in the fly, and that applies as well to the olfactory cortex; in particular, the precise cortical targets of the mitral cells in the mammalian olfactory bulb are not known. We have therefore focused on the processing inherent in the olfactory bulb network.

### Plasticity

It remains to note that synaptic plasticity is fundamental to any dynamic network. The clustered activity generated by odor stimuli is dependent on action potential backpropagation along the mitral cell lateral dendrites (according to the sequence of events discussed in Results), rather than the specific plasticity rule used to evolve the synaptic weights during odor presentation [18], [26]. Synaptic plasticity in the mitral-granule circuit has not been observed directly. We consider this lack of information as a shortcoming of the experimental techniques rather than a demonstration that there is no plasticity in the olfactory bulb. Indeed, recent studies have shown more or less direct evidence for plasticity of olfactory input in mitral cells [58]–[59], and in granule cells [60]–[62]. Although further experimental investigation is required to have a more detailed picture, one of the reasons that can explain the problems encountered in investigating plasticity in the olfactory bulb can be easily predicted by our model, and is related to network connectivity. The granule cell inhibitory synapses, once formed, can prevent any further activity-dependent plasticity of synapses far from the soma of the most active mitral cells (see Fig. 7). The model thus predicts that plasticity could be more easily characterized by recording from mitral and granule cells forming reciprocal perisomatic connections.

In conclusion, the present model suggests a physiologically based mechanistic explanation of how dendrodendritic excitation and inhibition, generated by experimentally measured odor inputs, can drive self-organization of the evolving dynamics in a large-scale olfactory bulb network. The process promotes the emergence of clustered organization in granule cell synaptic weight structure and mitral cell responses found in experimental studies [10], [16], [33]. The results demonstrate how odors can be represented in the olfactory bulb by a combination of temporal and spatial patterns, with both feedforward excitation and lateral inhibition via dendrodendritic synapses as the underlying mechanisms necessary and sufficient to maintain a sparse representation of odor identity.

## Materials and Methods

### Model cells

The network was composed of multi-compartment canonical models of 500 mitral and 10000 granule cells, implemented as described in our previous studies [18], [24], and connected through dendrodendritic synapses [63]. The canonical model for mitral cells was implemented with 312 compartments representing an axon, the soma, the apical dendrite, and 2 lateral dendrites each 1.5 mm in length, in the range indicated by anatomical measurements [40]. In the real case the 1.5 mm is the maximum extent of the mitral cell lateral dendrites. Cell stains show that some dendrites will be less, but a uniform extent enabled us to assess more clearly the extent of the lateral inhibition under different conditions in the model. Uniform passive properties were used, with R_a_ = 150 Ω·cm, τ_m_ = 20 ms, and R_m_ and C_m_ adjusted to obtain an input resistance of about 100 MΩ [40]. Resting potential was set at −65 mV and temperature at 35°C. Cells were modeled as regular firing cells (see Fig. 1d in Ref. [64]), with Na, K_A_, and K_DR_ conductances uniformly distributed over the entire dendritic tree [65]. Kinetics for the Na conductance were from hippocampal pyramidal neurons [66], whereas those for K_A_ and K_DR_ were from mitral cell data [67]. This resulted in the mitral cells firing within the range of experimentally observed firing rates and, in further agreement with experimental findings, somatic action potentials backpropagated at full amplitude up to the tuft [68], and an AP could initiate in the tuft or in the primary dendrite for moderate to strong odor inputs [69] (see also Fig. 1e in Ref. [64]. Granule cells (GC) were modeled with a soma and a 20 segment radial dendrite (250 µm of total length) representing the dendritic tree. Na^+^ and K_A_ channels were distributed throughout [70]–[72] whereas K_DR_ was present only in the soma [70]. In agreement with experimental findings [70], the dendritic K_A_ resulted in a significant effect on the spike latency of these cells (see Fig. 1Bb of Ref. [24]), and adaptation under strong inputs [73]–[74].

It should be noted that a number of additional ion channels and mechanisms were not included in our model cells. Virtually all of them, such as additional K^+^ conductances, I*_h_* current, persistent Na^+^ current, Ca^2+^ and Ca^2+^-dependent currents, but also activity-dependent changes in channels density or kinetic, non-uniform channels distribution, intracellular Ca^2+^ dynamics, intercellular variability, additional external inputs etc, may result in some modulation of the results. This is precisely why we did not include them in the model at this stage. Our focus has been on understanding the processes underlying lateral and feedback inhibition and their main consequences. Future works will eventually investigate how and to what extent additional cell types, mechanisms, and external inputs can affect the basic findings shown in this paper.

### Network elements

There are a number of different types of cells in the olfactory bulb network, each of them carrying out or involved in functions that in most cases are not understood. Rather than include a priori in the network all cell types for which there was some experimental suggestion on their electrophysiological properties or function, we decided to keep the model simple enough to allow a clear identification of the key mechanisms underlying the effects of lateral and feedback inhibition, and constrained enough by experimental findings to allow not only a direct comparison of the results with specific experimentally observable but also to make experimentally testable predictions. From this point of view, as shown and discussed in our previous papers [e.g. 18], [24], as long as there are action potentials propagating along the mitral cell lateral dendrites and granule cells making local plastic synaptic dendrodendritic connections with them, the network will self-organize following an odor presentation. The level of details included here are thus those necessary and sufficient to have these mechanisms in place. With respect to our previous reduced model network, in this paper we added a new level of realism in network size and connectivity and odor input, obtaining a more realistic network dynamics that has not been achieved by other modeling approaches.

### Synaptic properties

Network connectivity is presented and discussed in Results. Effective dendrodendritic coupling between granule cell synapses and mitral cell secondary dendrites was implemented by connecting a GC synapse, containing the same proportion of AMPA and NMDA channels, with the appropriate compartment of mitral cells secondary dendrites containing GABA channels. The AMPA conductance was modeled as an alpha-function with a time constant of 3 ms and a reversal potential of 0 mV. The NMDA conductance was based on a NEURON model [75] of experimental findings [76]–[77], assuming an external magnesium concentration of 1 mM and a reversal potential of 0 mV. The model parameters were adjusted to obtain a time-to-peak and decay time constant of 10 and 50 ms, respectively. A double exponential function was used to model the GABA conductance, with a reversal potential of −80 mV. Rise and decay time constants were 1 and 200 ms, respectively, implicitly taking into account the mechanisms underlying the overall time course observed experimentally for the inhibitory potential elicited by a single AP in a mitral cell [78]. Unless otherwise noted, the peak excitatory and inhibitory conductances of each synapse were 0.5 nS and 3 nS, respectively, equivalent to about 1 and 5 real individual synapses, respectively [78]. Synapses (excitatory or inhibitory) were activated whenever the corresponding presynaptic compartment reached the threshold of −40 mV, in agreement with experimental findings [79] suggesting that recurrent inhibition of the secondary dendrite of a mitral cell does not necessarily require the generation/propagation of an action potential to the soma of the GC.

Synaptic weights of dendrodendritic synapses started at zero and, in response to odor input, followed the same plasticity rule previously used [18], [26]. Briefly, each component (inhibitory or excitatory) of each dendrodendritic synapse was independently modified according to the local membrane potential, of the lateral dendrite of the mitral cell or the granule cell synapse, to calculate the instantaneous presynaptic ISI. After each spike, the peak conductance, *w*, of any given synapse was updated from its current value *w_{exc,inh},p_ = g_syn,{exc,inh}_•S(p)* to *w_{exc,inh},p+Δ_ = g_syn,{exc,inh}_•S(p+Δ)*, where the function *Δ* = {0,+1,−1} followed a classical scheme [80]–[81] in which *Δ* = 0 for ISI>250 ms, *Δ* = −1 for 30<ISI<250 ms, and *Δ* = 1 for ISI<30 ms. The typical sigmoidal activation function *S(p)* [82] was defined as *S(p) = 1/(1+*exp*((p-25)/3))*. In this way, the weight (i.e. the peak synaptic conductance) of any given synapse could go from a fully depressed (for *p≈0*) to a fully potentiated state (for *p≈50*), or vice-versa, in about 50 consecutive spikes of the appropriate frequency. Unless explicitly noted otherwise, *p* = 0 at the beginning of a simulation. This plasticity rule is non-hebbian, since it changes a synaptic weight ignoring any postsynaptic activity. However, we have previously noted, shown, and discussed [18], [26] that the formation of synaptic clusters consistent with those observed experimentally [10] is robust and does not depend on the specific choice for the functional form used to update the synaptic weights.

Odor stimulation of mitral cells was modeled using a synchronous activation, in all tuft compartments, of synaptic inputs with a double exponential conductance change (20 and 200 ms rise and decay time, respectively), and an individual peak conductance in the range 0–0.5 nS. This corresponds to a total aggregate input conductance of up to 10 nS. It elicits 6 somatic spikes during a single activation, within the range observed experimentally for the number of APs generated during a respiratory cycle [81]. To simulate the learning process of a given odor, unless explicitly noted otherwise, the synaptic inputs to the mitral cells belonging to the active glomeruli were activated at a random frequency in the range of 2–10 Hz, corresponding to the range of natural sniffing frequency during explorative behavior in rats [55]. An independent random background synaptic activity (eliciting spikes at around 2 Hz), was added to the soma of all cells to model *in vivo* activity. During preliminary tests, we found that a 10 sec odor presentation was sufficient to stabilize synaptic weights and cell activity. All simulations were thus carried out for 10 sec. Experimentally, the instantaneous firing rate of individual mitral cells during an odor presentation is highly variable (e.g. [39]), depending on many factors such as input strength, stimulation/sniffing frequency, and network interactions. In our model, the instantaneous firing rate of mitral cells activated by an odor was up to 75 Hz, within the physiological range observed in vivo.

### Simulations

All simulations were carried out with the NEURON simulation program (v7.3, [83]) on a BlueGene/Q IBM supercomputer (CINECA, Bologna, Italy) or a Cray XE6 system (INCF, Lindgren, Sweden). Under control conditions, the model network was composed by a system of 12,877,500 non-linear differential equations, and a typical 10 sec simulation required about 2 hours using 400 processors using a fixed time step of 25 µs. To test the robustness of the results, we ran additional test simulations of odor k3-3 using a smaller time step (10 µs), cells modeled with a larger number of compartments (5 µm membrane segments), or a different random number sequence. In all cases (see Fig. S2), the final inhibitory weights distribution was statistically indistinguishable from control (Mann-Whitney rank sum test *p*>0.913). The model and simulation files are available for public download under the ModelDB section of the Senselab database suite (http://senselab.med.yale.edu, acc.n. 144570). Movies from each simulation were created offline, with a custom developed command-line environment that generates a movie from the spike times. We used the open-source tool Octave (www.gnu.org/software/octave) to create all frames, updating each cell's visual aspect in each frame, and pipeline them to concurrent instances of POVRay (www.povray.org), a multi platform, free tool for creating high quality three dimensional photo-realistic scenes. All frames were finally joined together into a compressed MPEG-4 movie using FFmpeg (www.ffmpeg.org). A typical hi-resolution (1280*720 pixels) 10 sec simulation movie is composed by 600 frames. On 16 quadcore CPUs interconnected via InfiniBand, the rendering of all frames takes less than 4 minutes.

## Supporting Information

Figure S1 Synaptic weights between mitral and granule cells after 10 sec odor presentations for each of the 72 odors used in this paper. **A–D**) panels presents the results for a single odor. In each panel, the *top* histogram represents the input strength on each mitral cell, and the *middle* and *bottom* plots represent the normalized excitatory and inhibitory peak synaptic conductance after 10 sec of odor presentation, respectively; (dark purple: 0, white: 1).
(PDF)

Figure S2 Results from test simulations of odor k3-3 for 10 sec, using the control values used for all simulations, a smaller time step (10 µs instead of 25), modeled with a larger number of compartments (5 µm membrane segments, instead of 10–30 µm), or a different random number sequence.
(PDF)

Video S1 Movie illustrating somatic activity of mitral cells (red cones) and granule cells (green spheres) during a 10 sec simulation. Synaptic weights start from 0, and the network self-organizes during presentation of odor k3-3, an aliphatic ketone. To illustrate better the network activity, spikes from 100 granule cells below the most active mitral cells have been associated with sound clicks. In order to meet the journal's limit on files size, frames have been highly compressed. A full HD resolution version (about 200 Mb) is available for public download on the ModelDB database (http://senselab.med.yale.edu/modeldb/default.asp, acc.n.144570).
(AVI)
