# Supplementary figures and images for: Sparse Distributed Representation of Odors in a Large-scale Olfactory Bulb Circuit
Source: PLoS Comput Biol. 2013 Mar 28;9(3):e1003014. doi: 10.1371/journal.pcbi.1003014 (PMC3610624; doi:10.1371/journal.pcbi.1003014)

**A**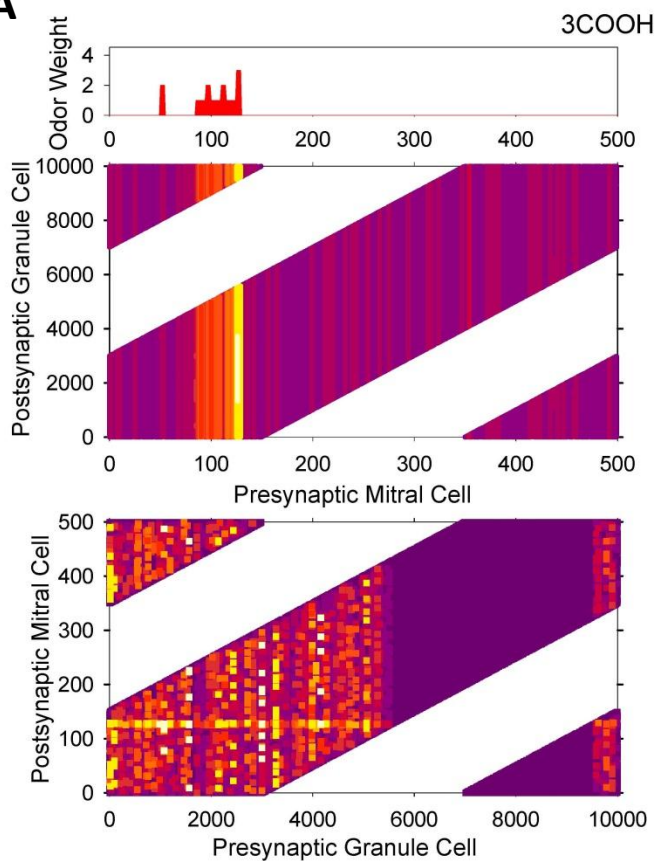**B**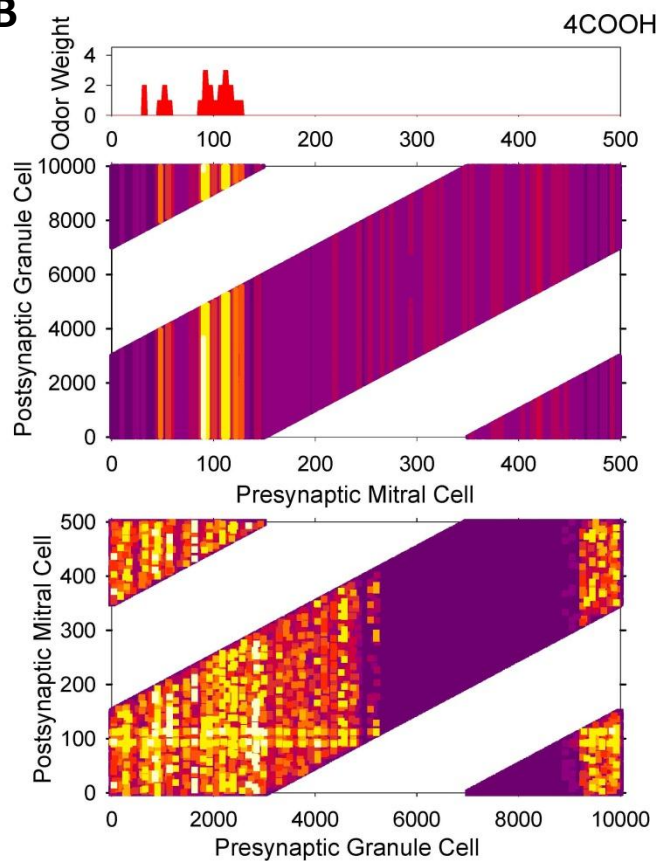**C**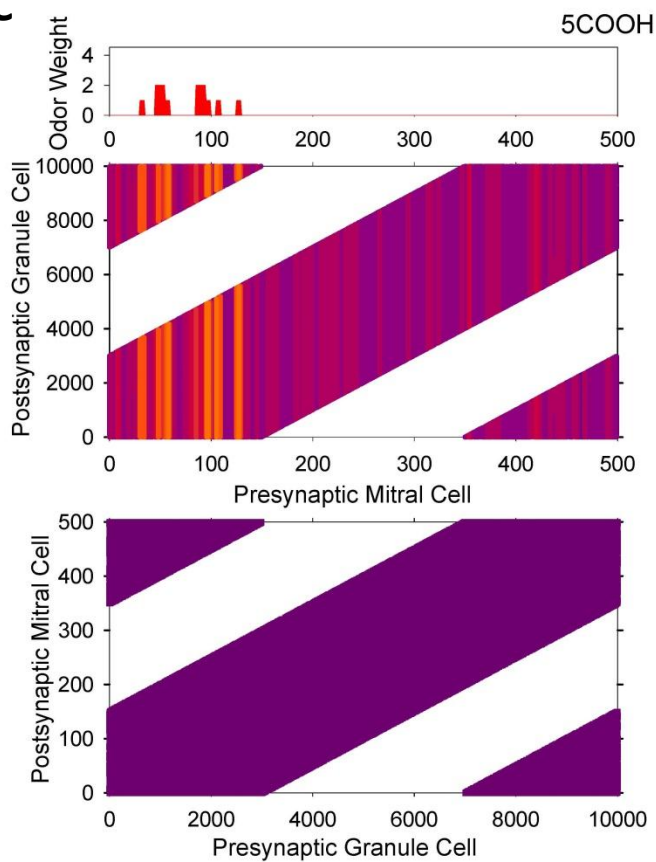**D**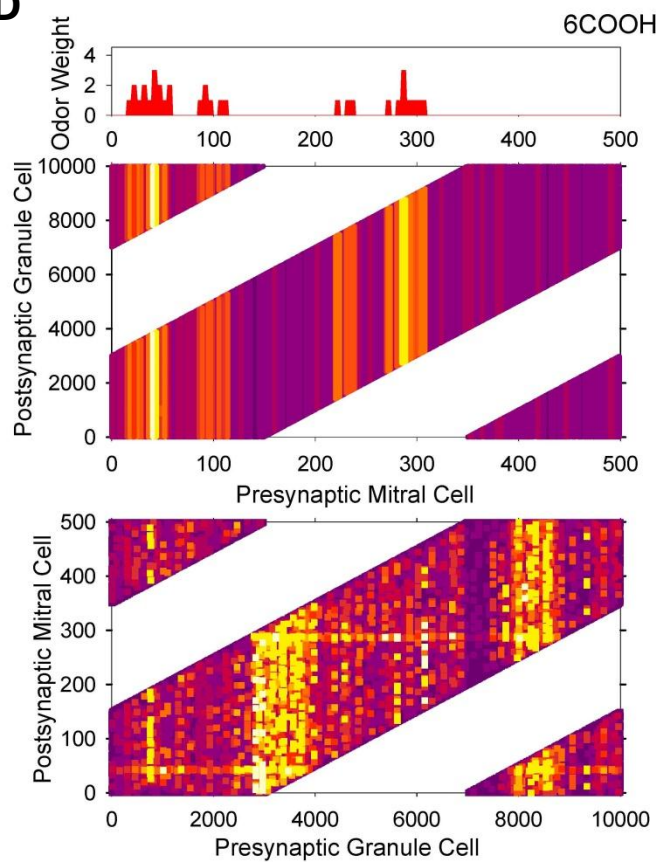

**A**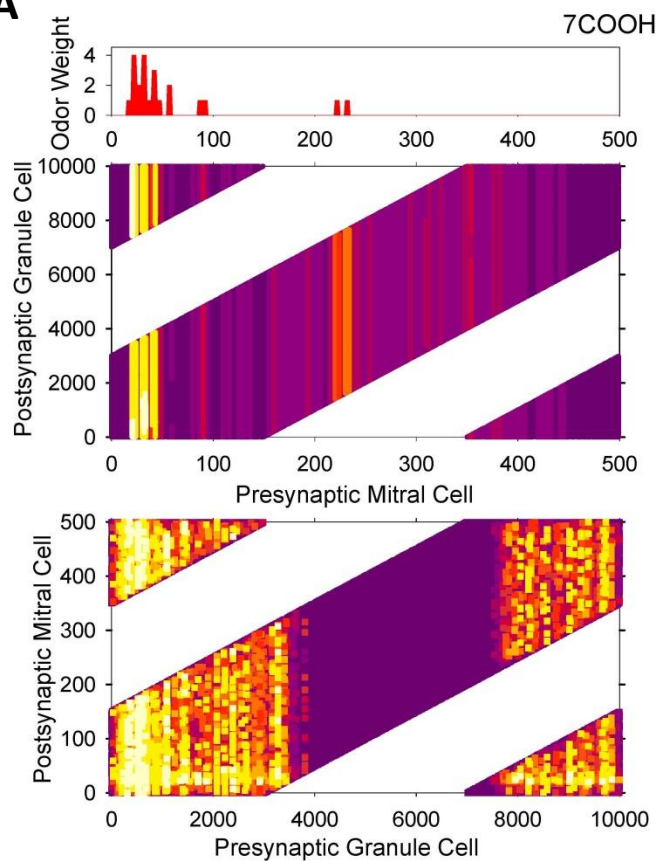**B**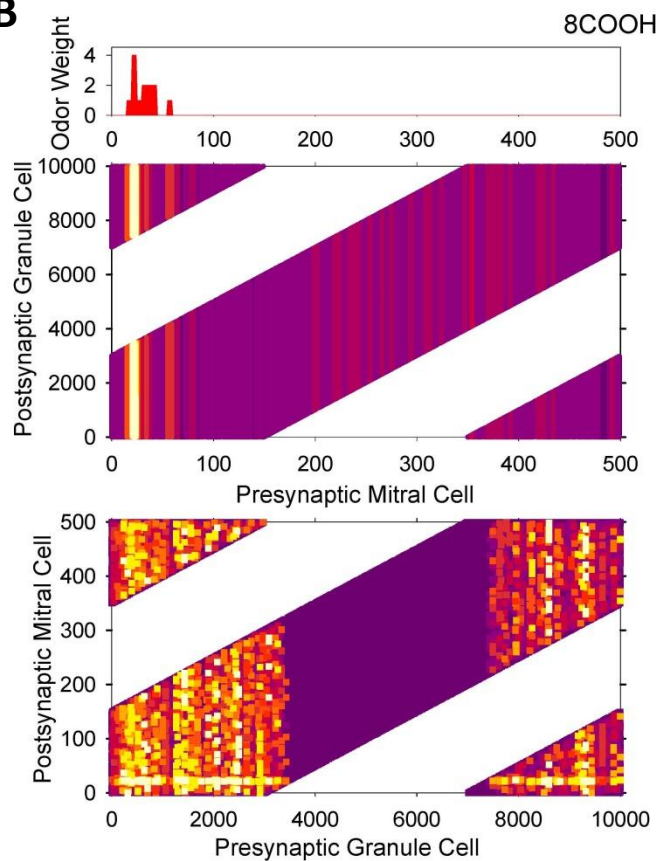**C**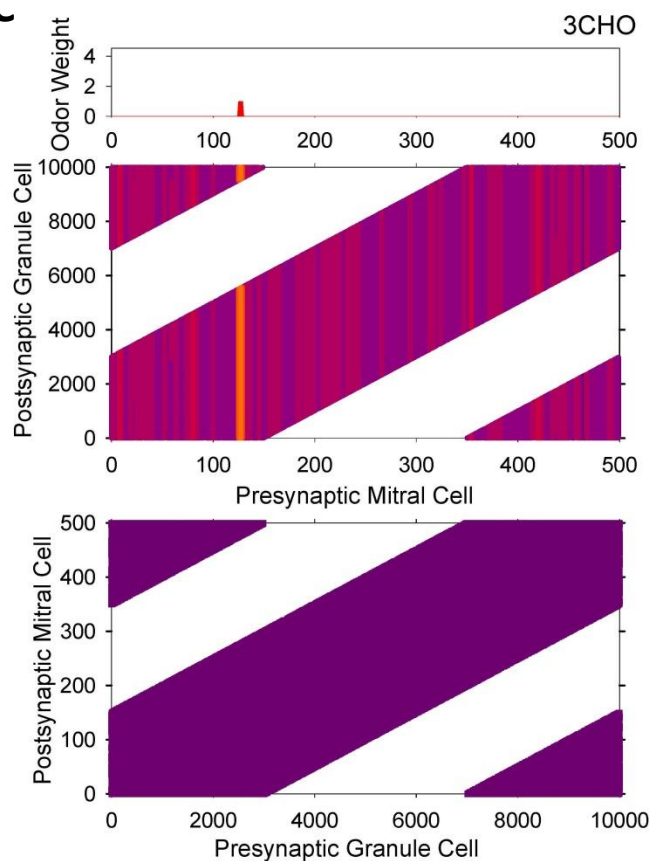**D**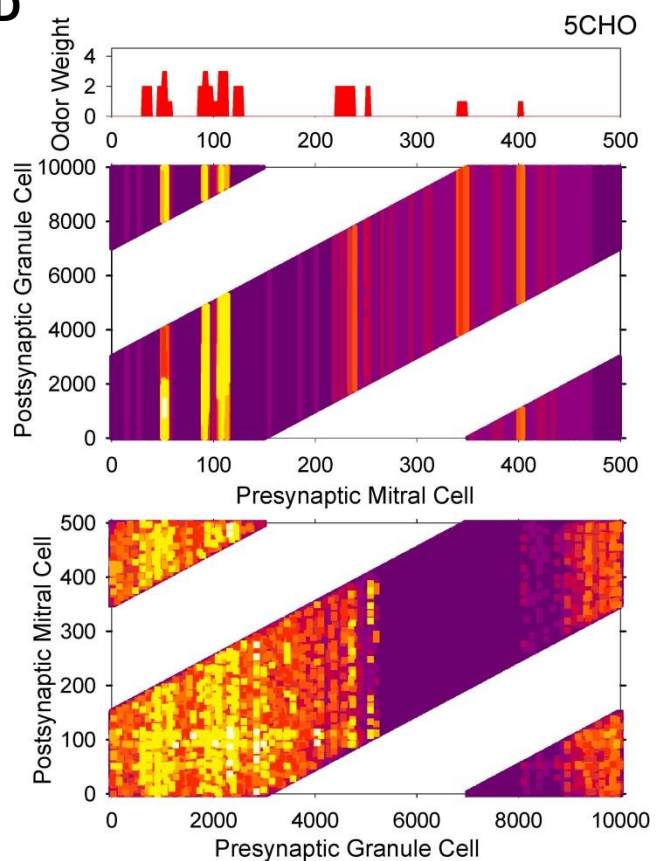

**A**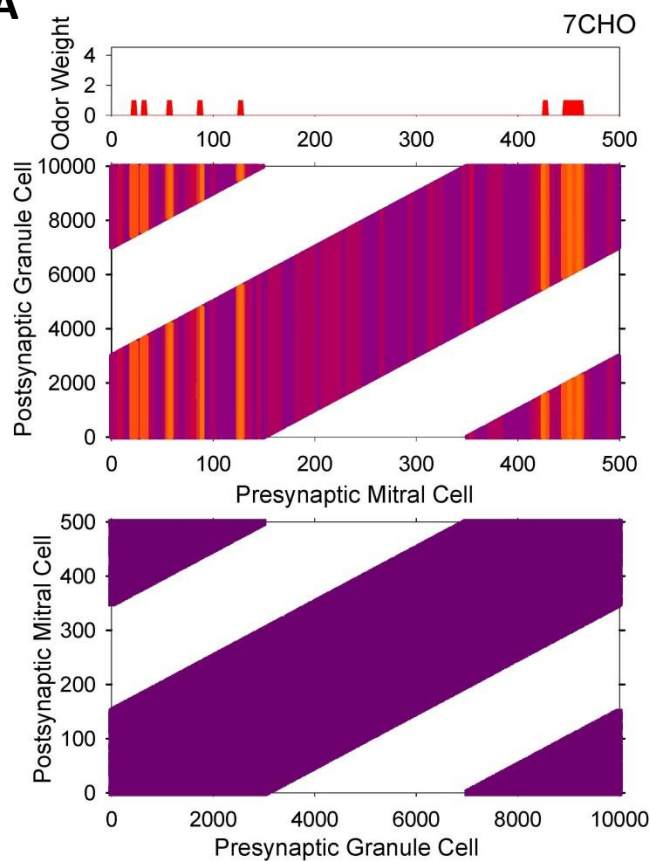**B**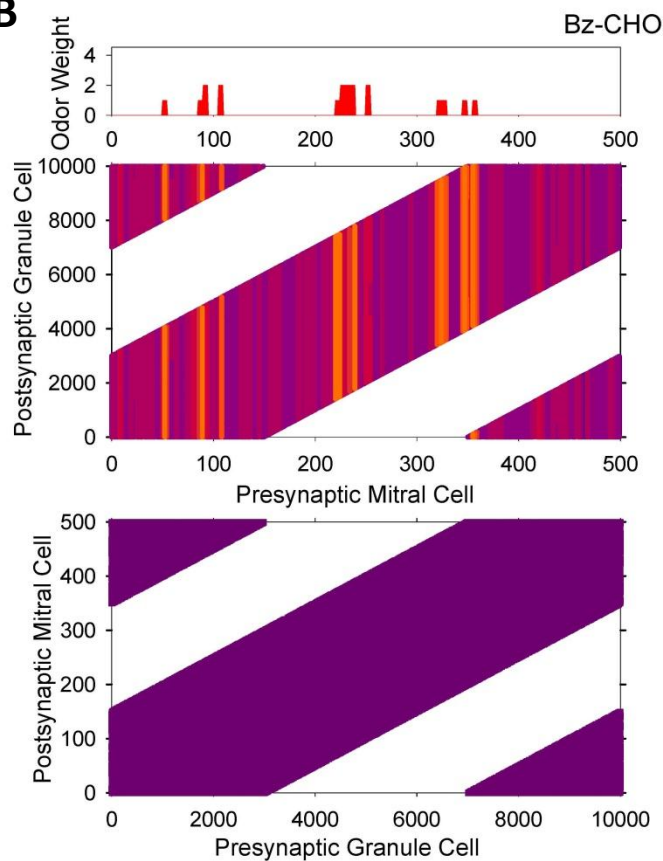**C**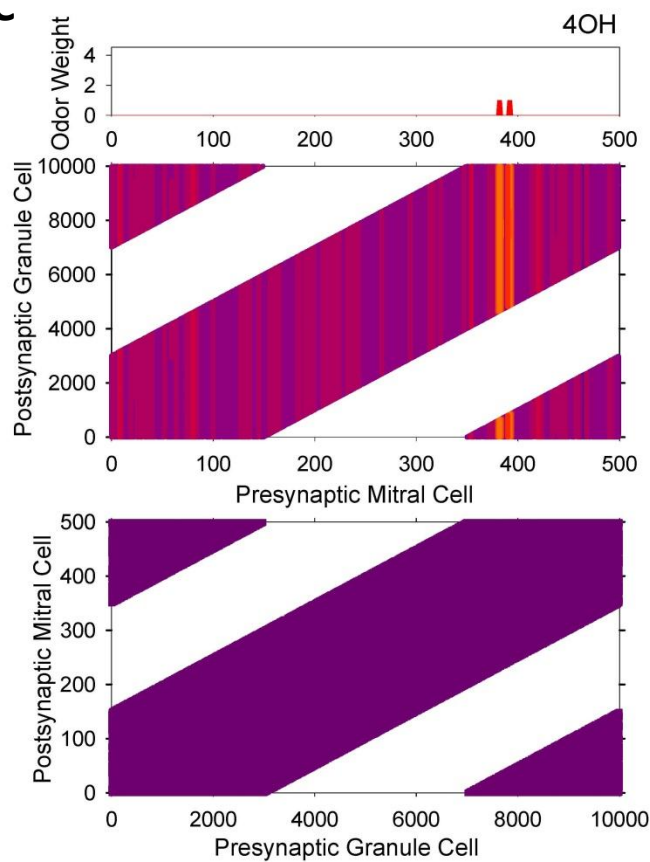**D**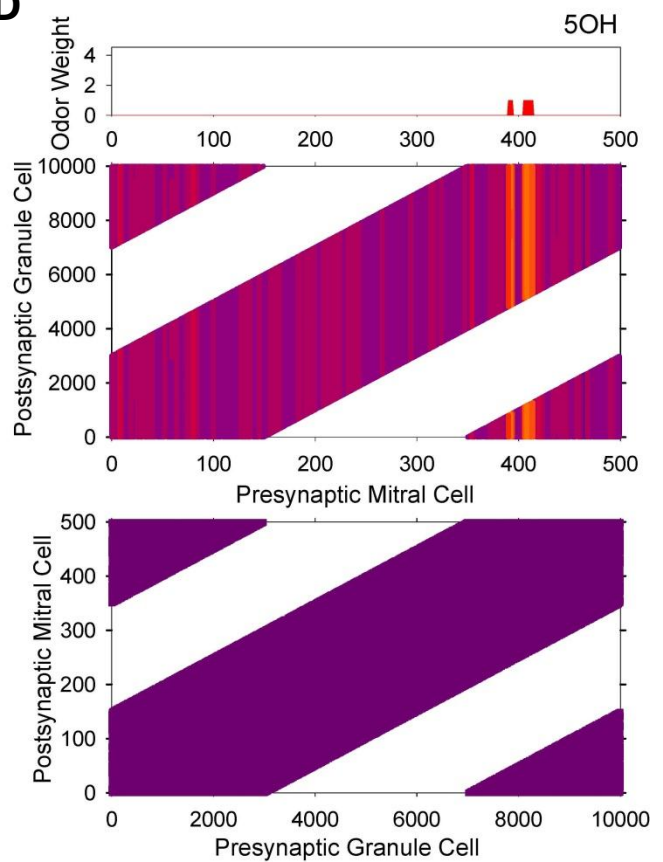

**A**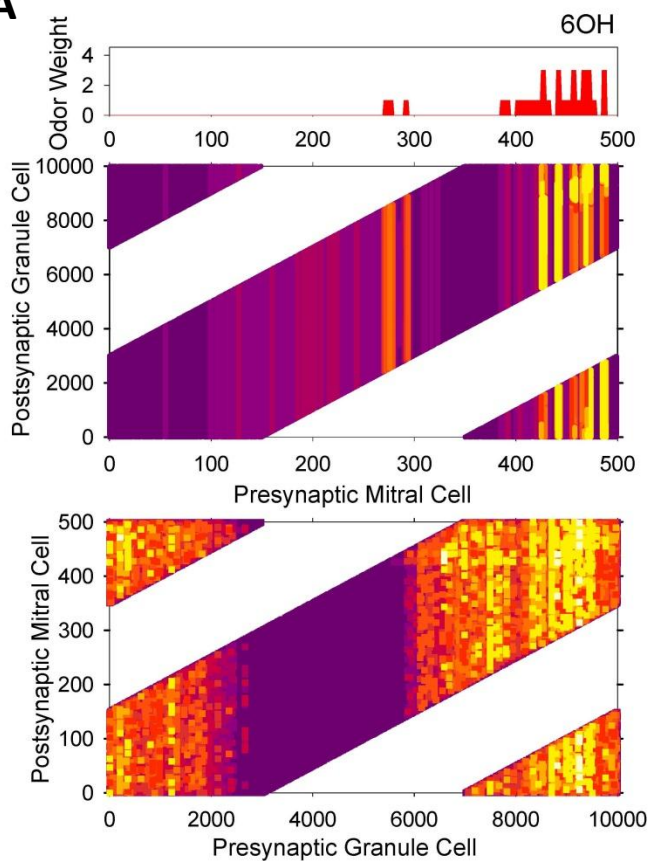**B**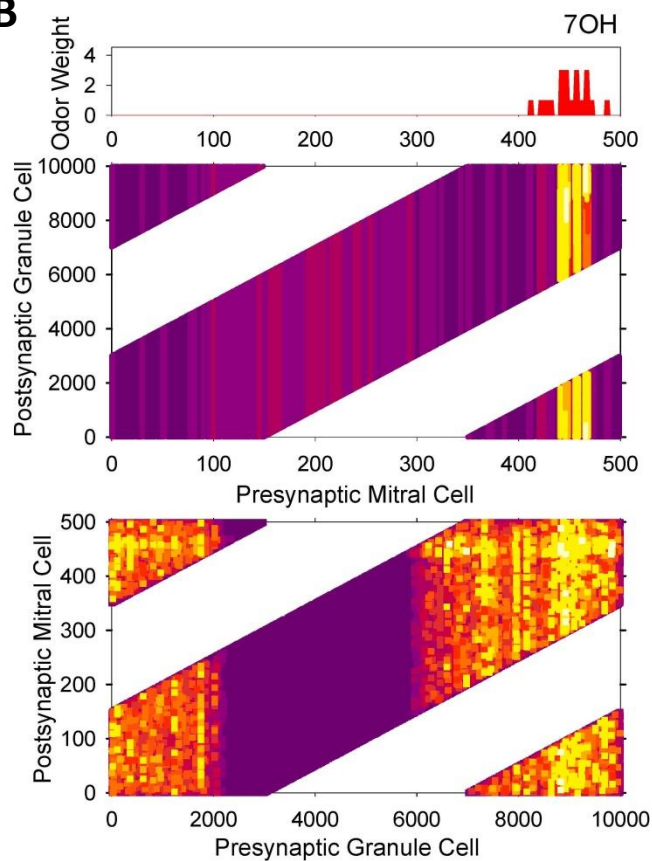**C**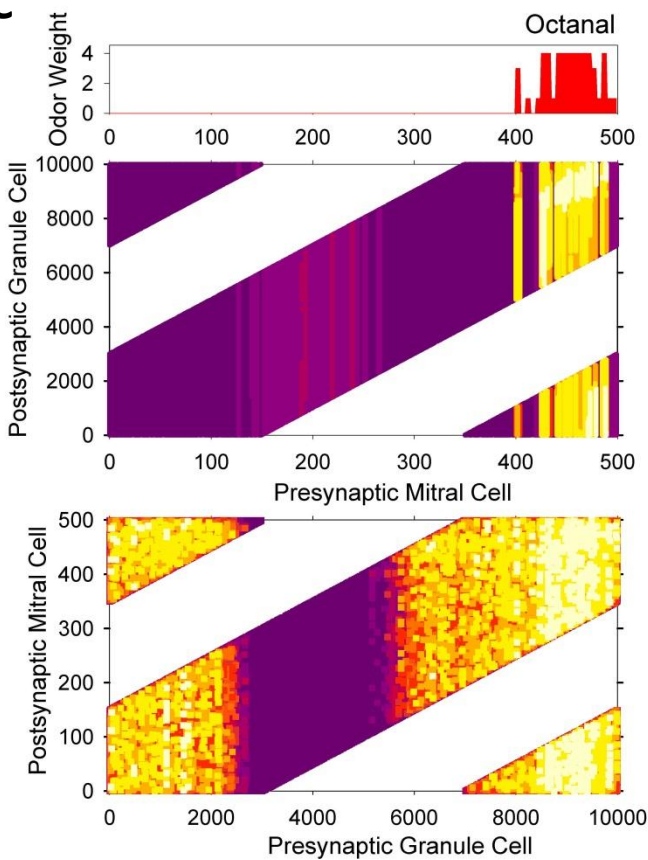**D**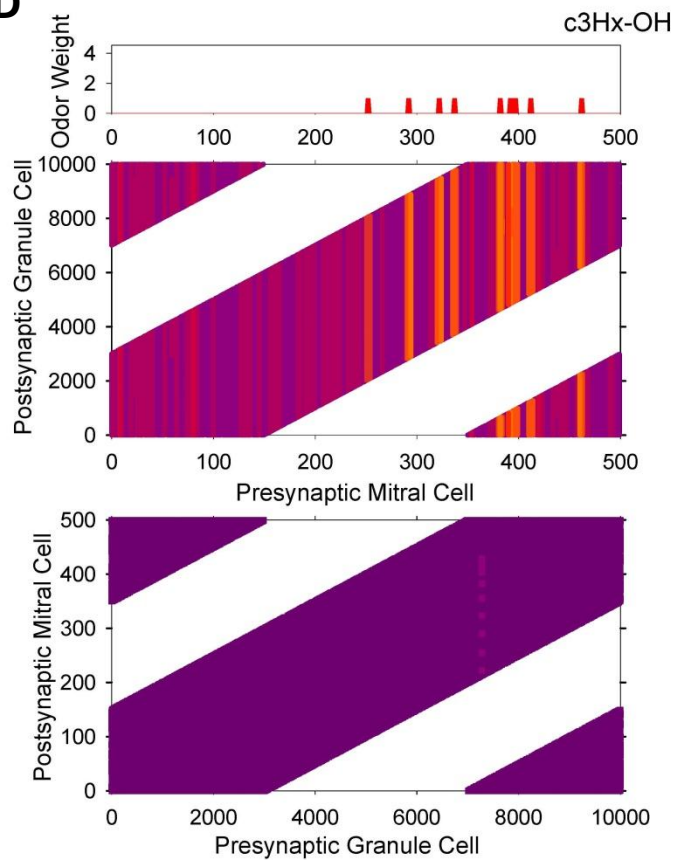

**A**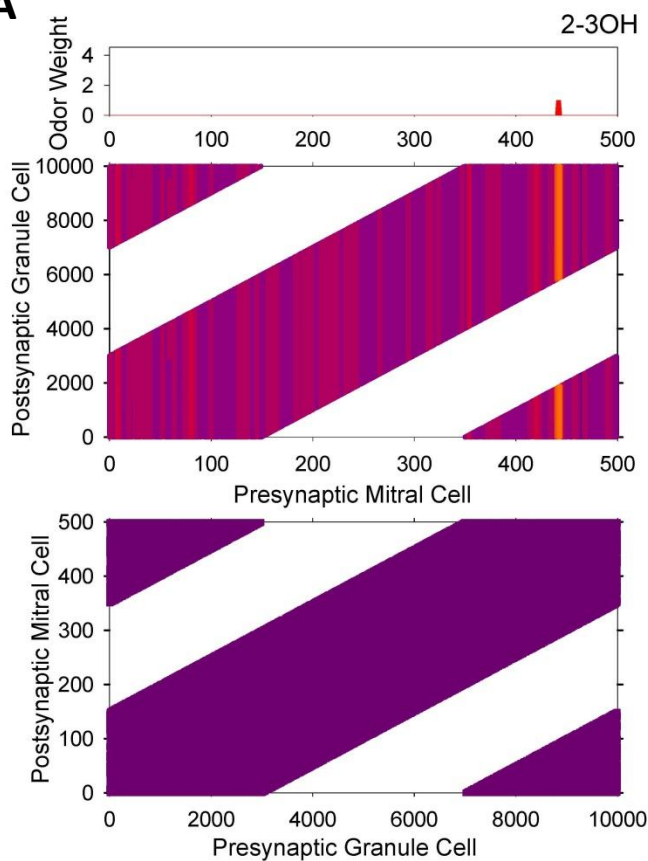**B**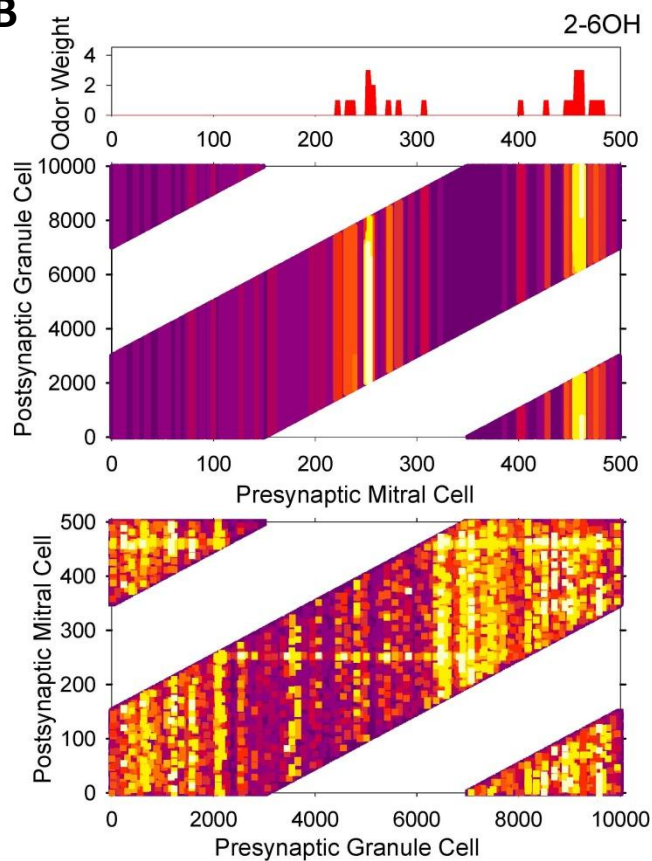**C**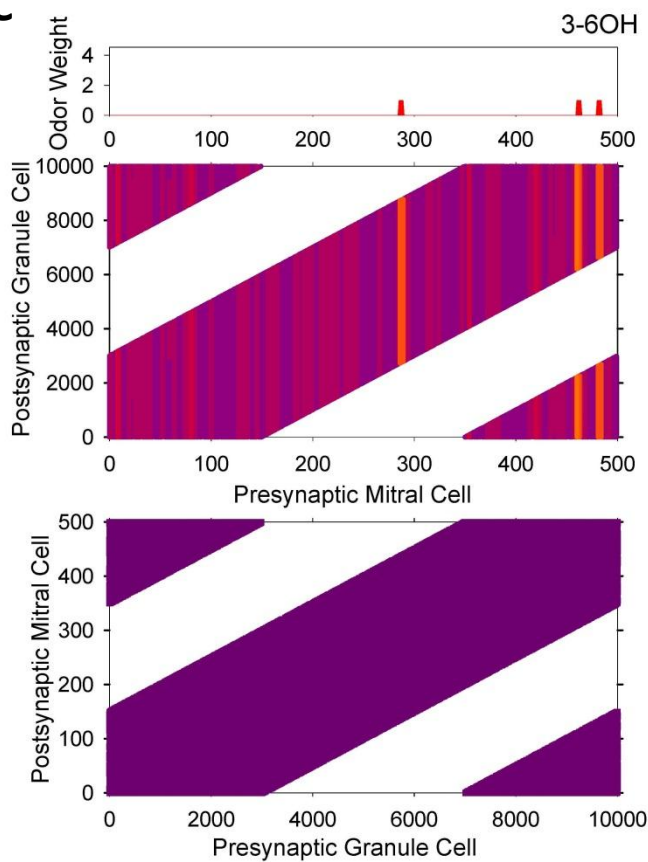**D**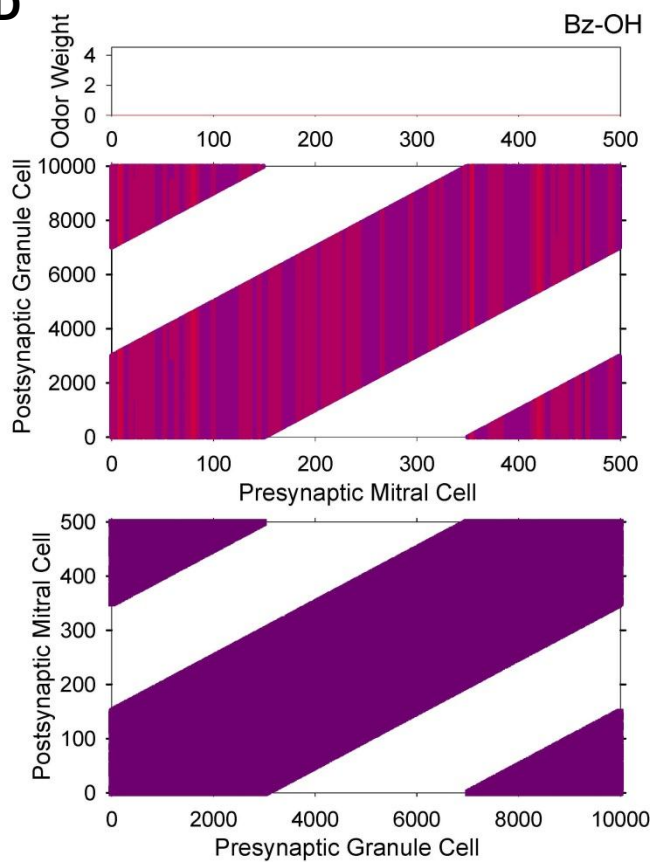

**A**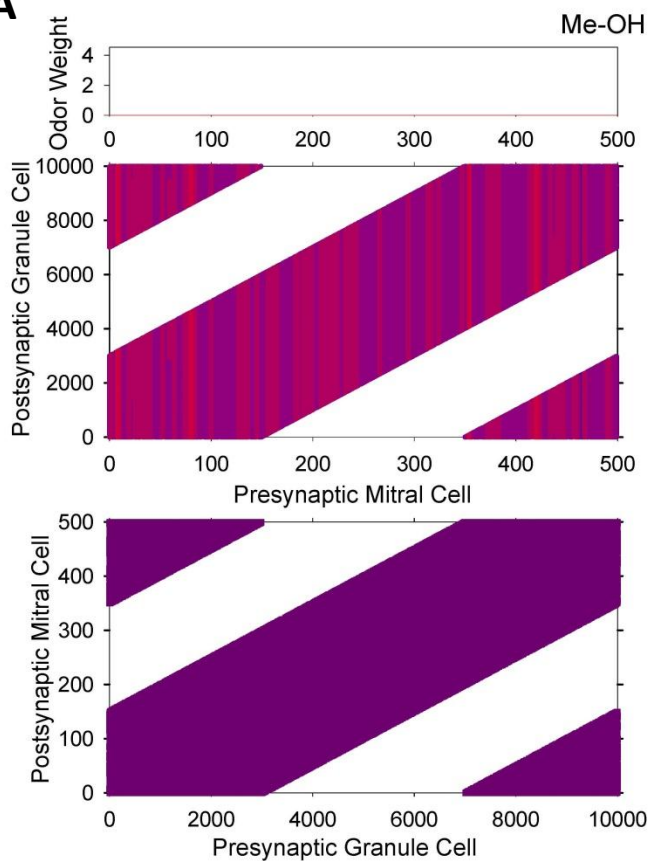**B**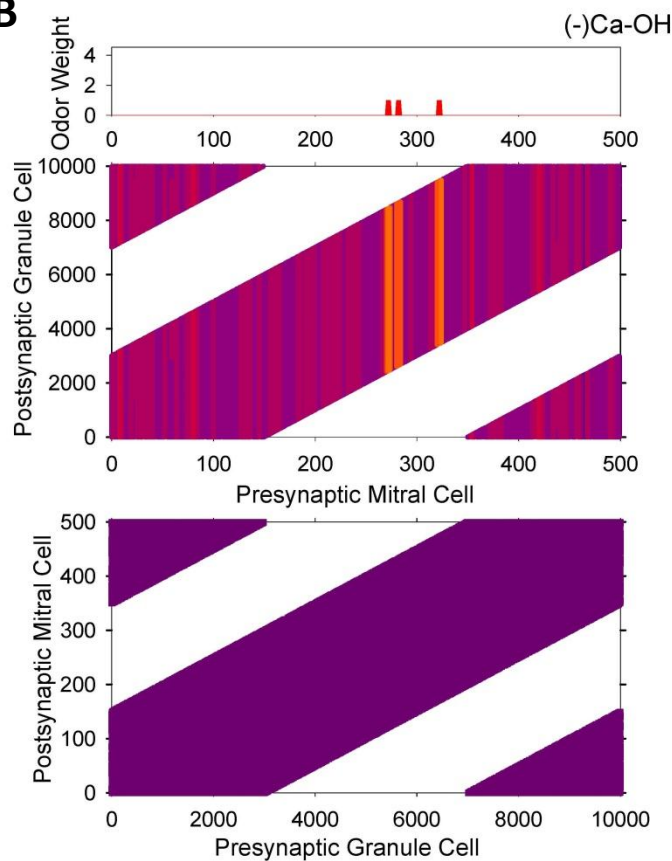**C**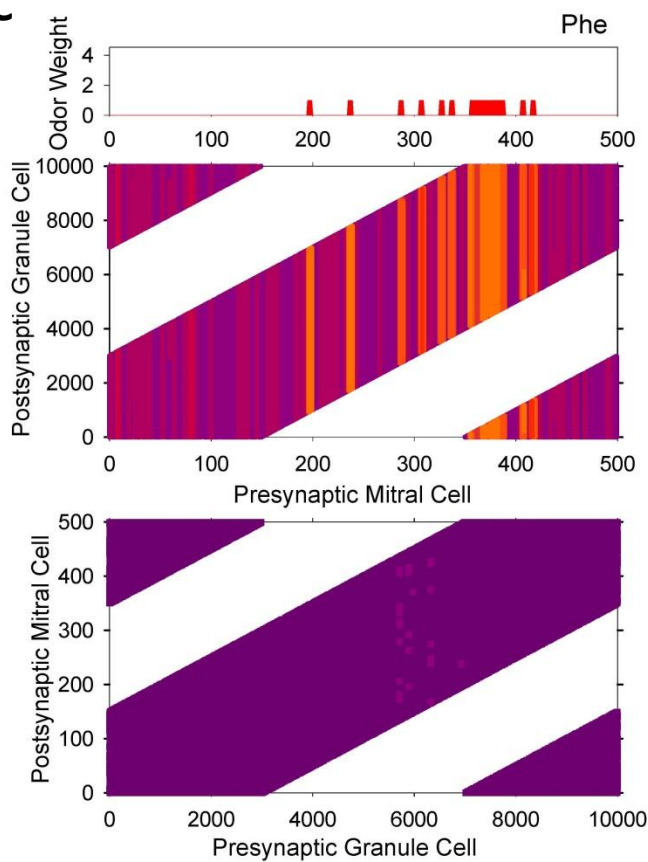**D**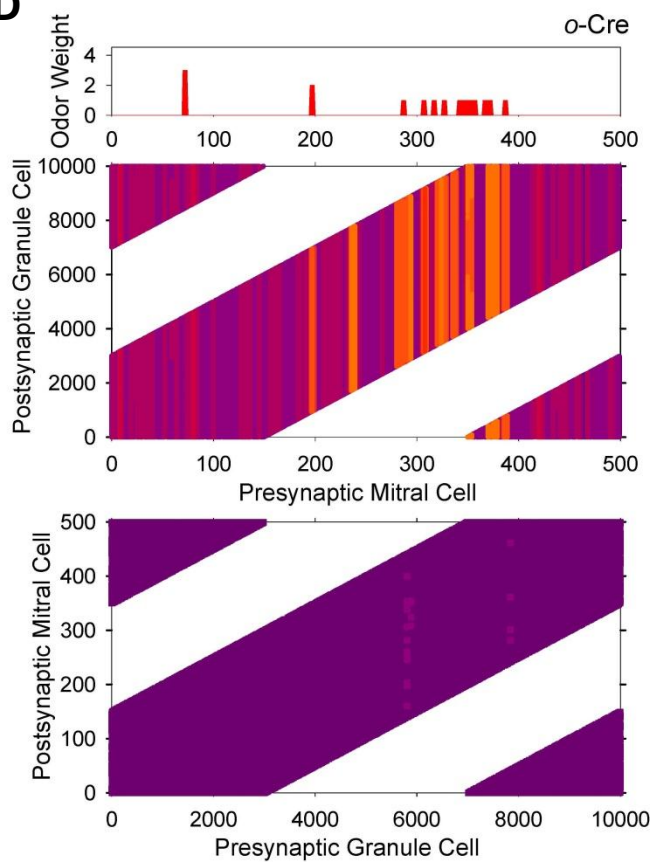

**A**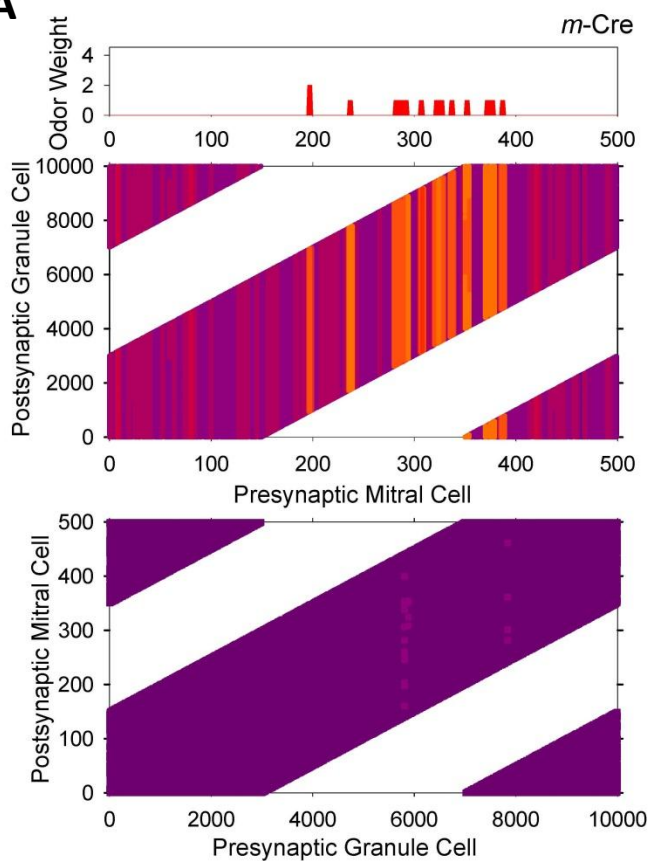**B**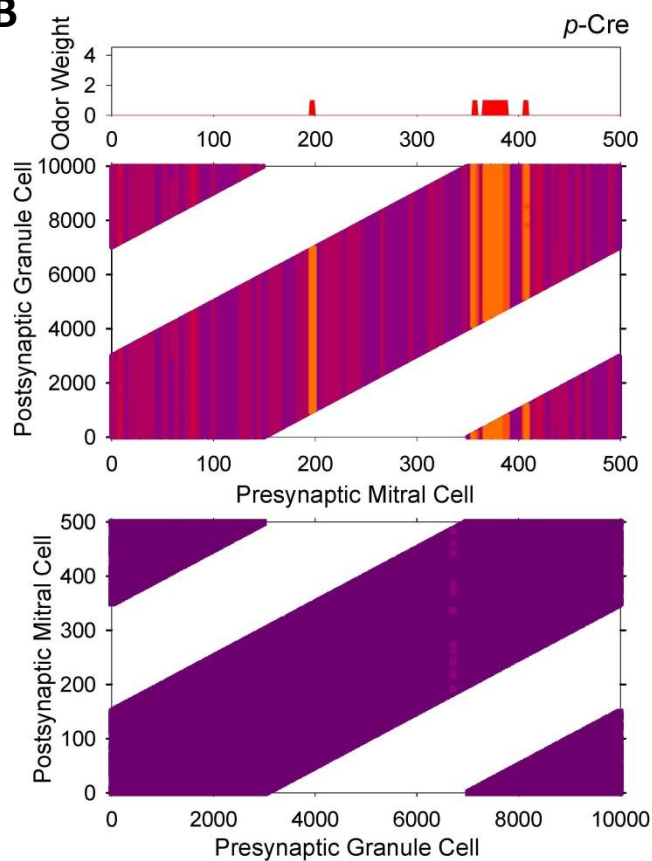**C**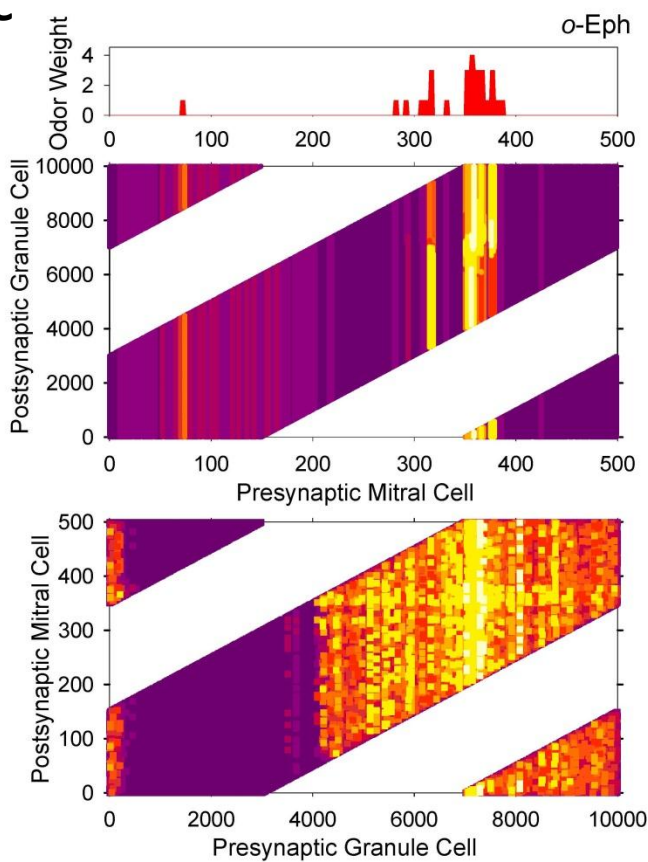**D**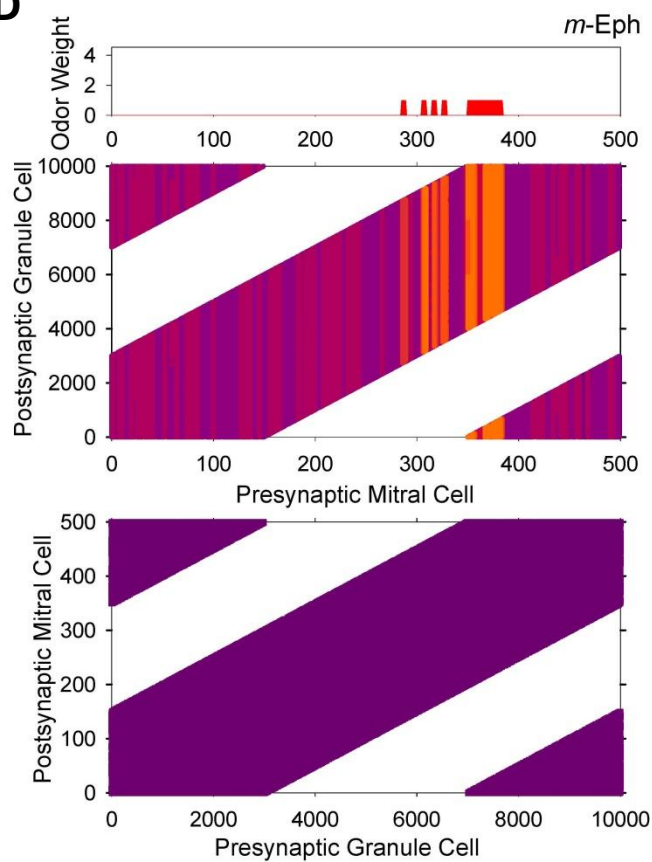

**A**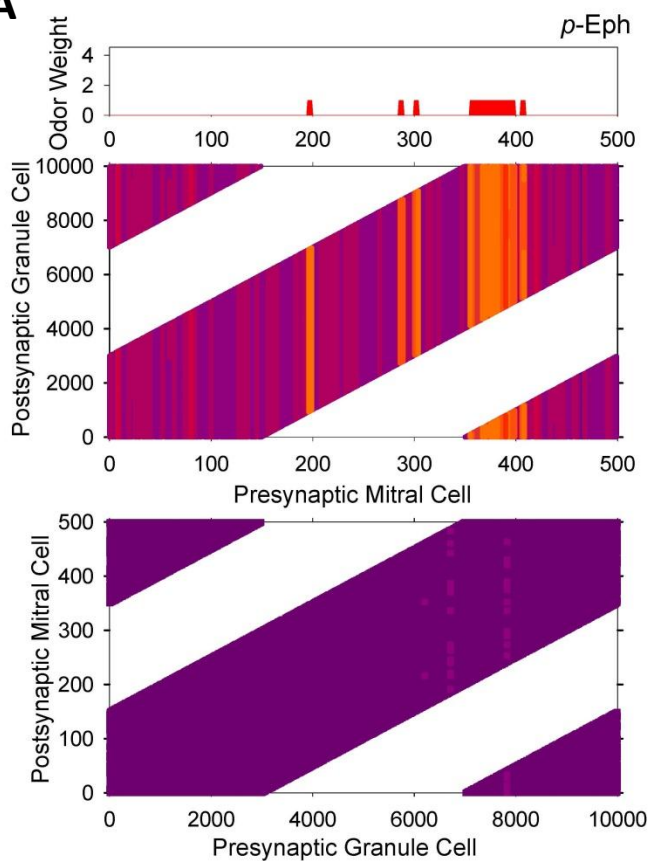**B**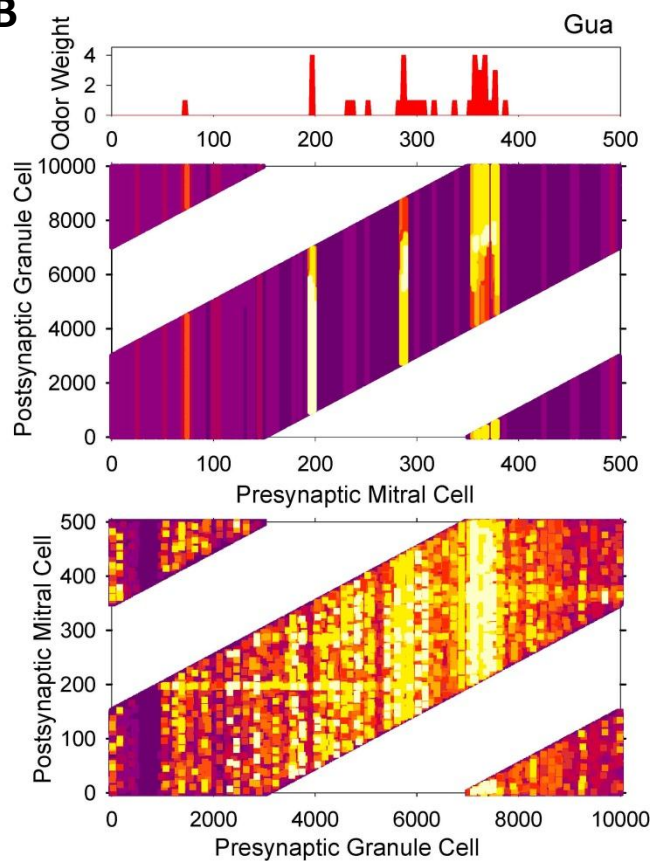**C**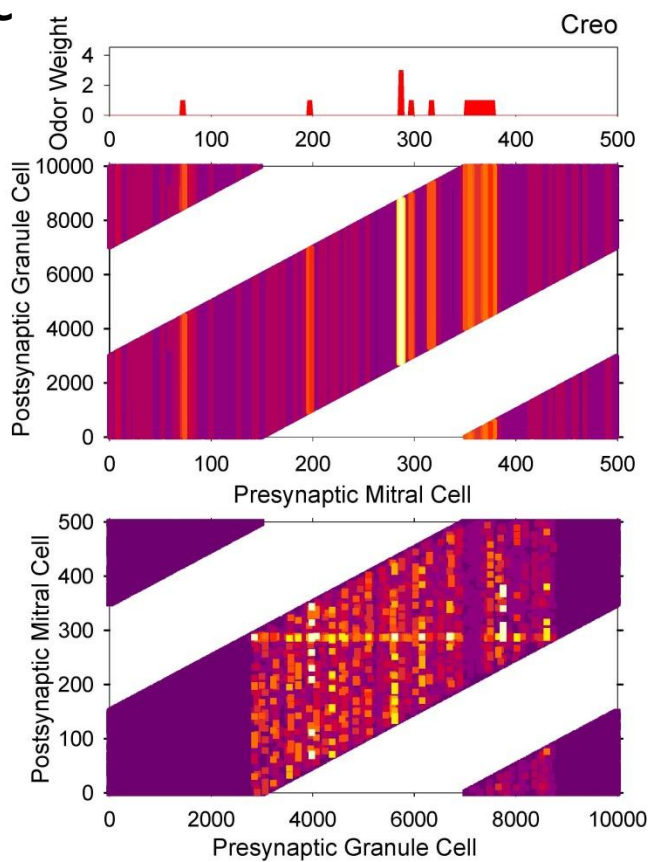**D**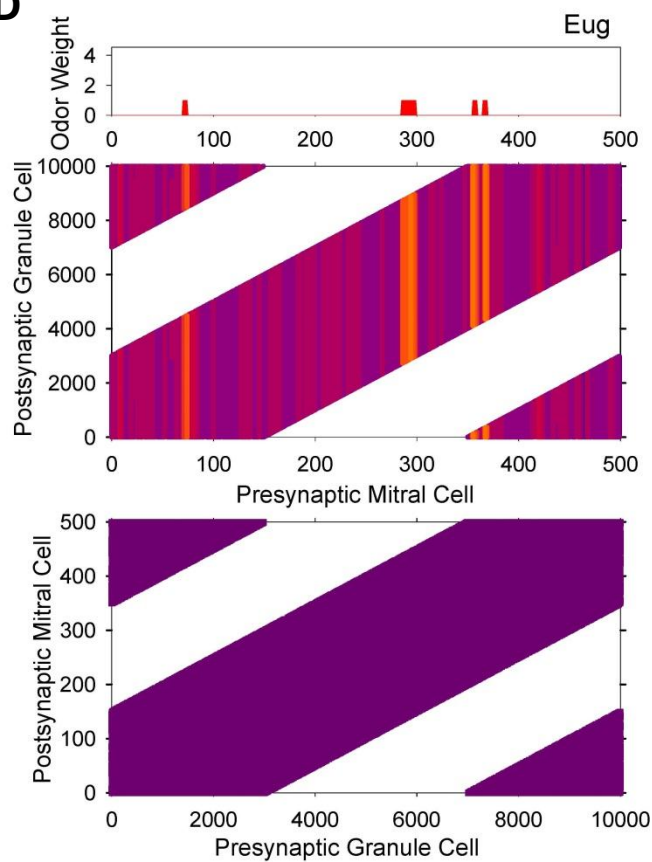

**A**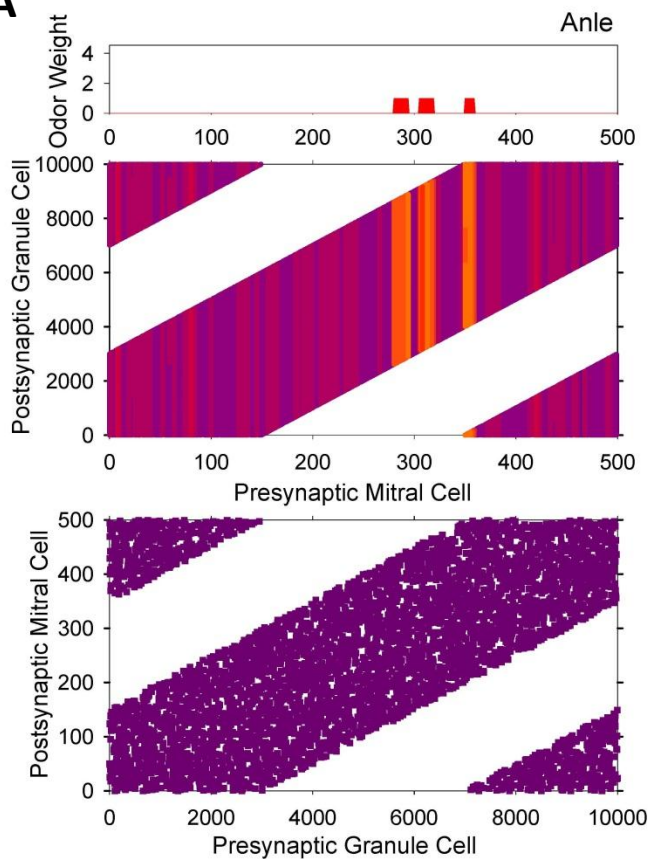**B**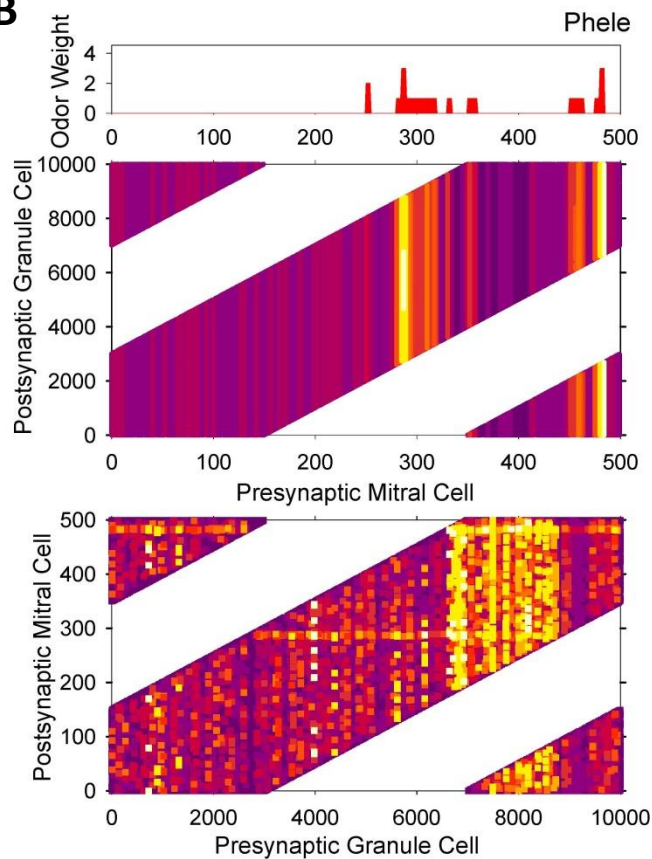**C**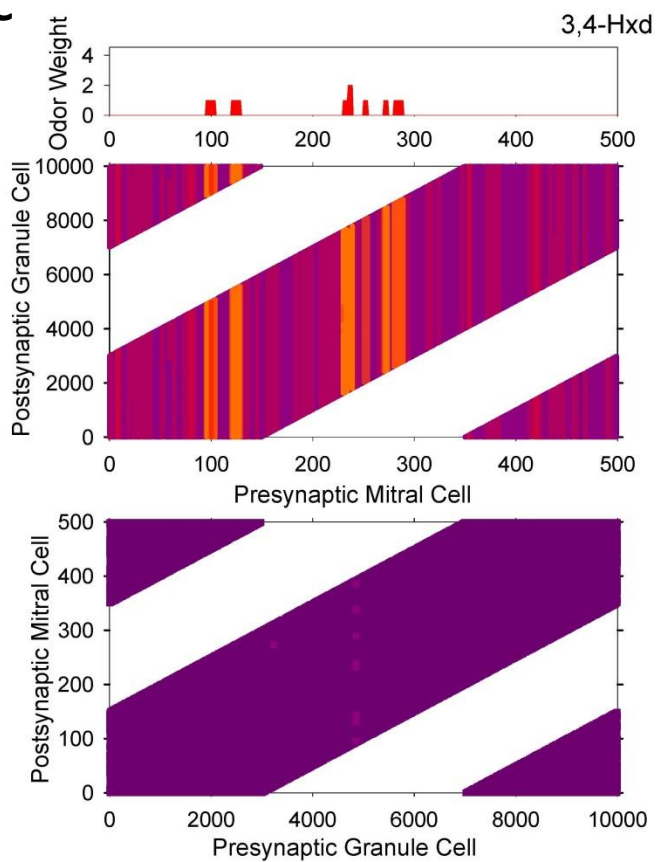**D**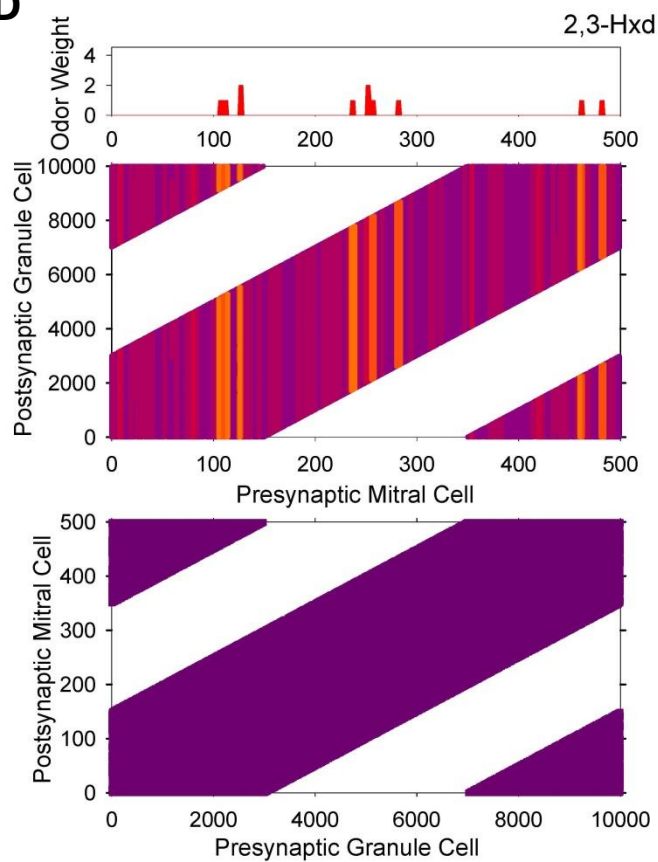

**A**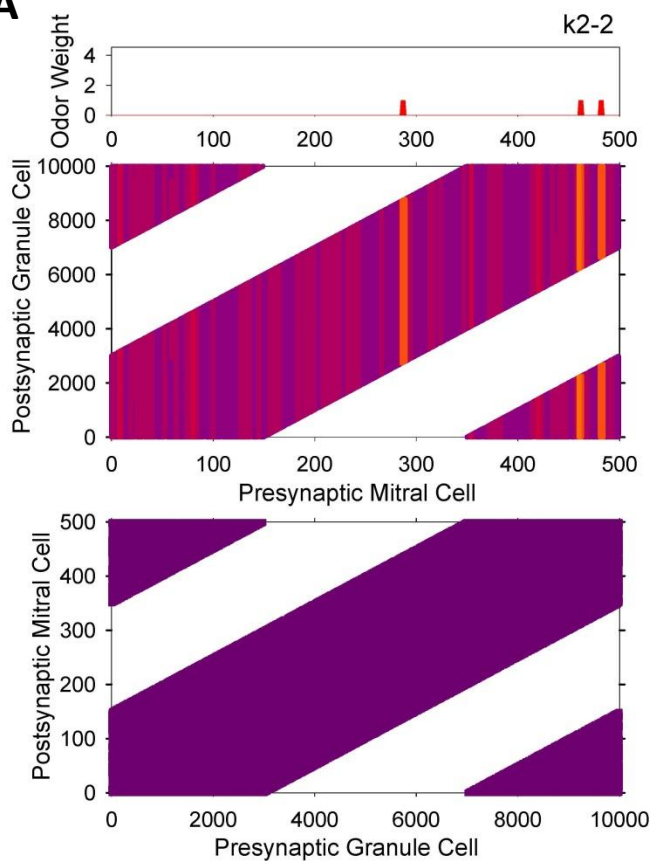**B**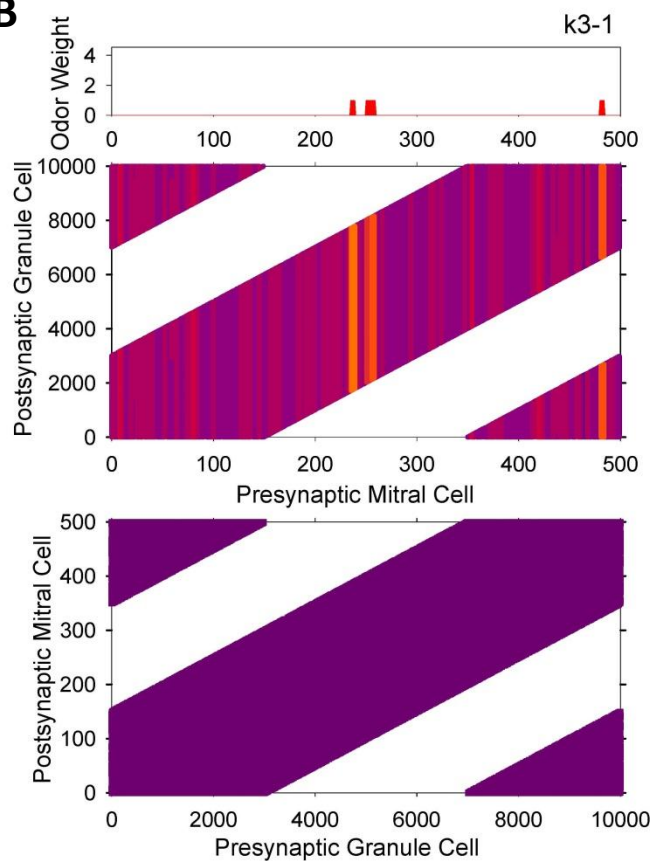**C**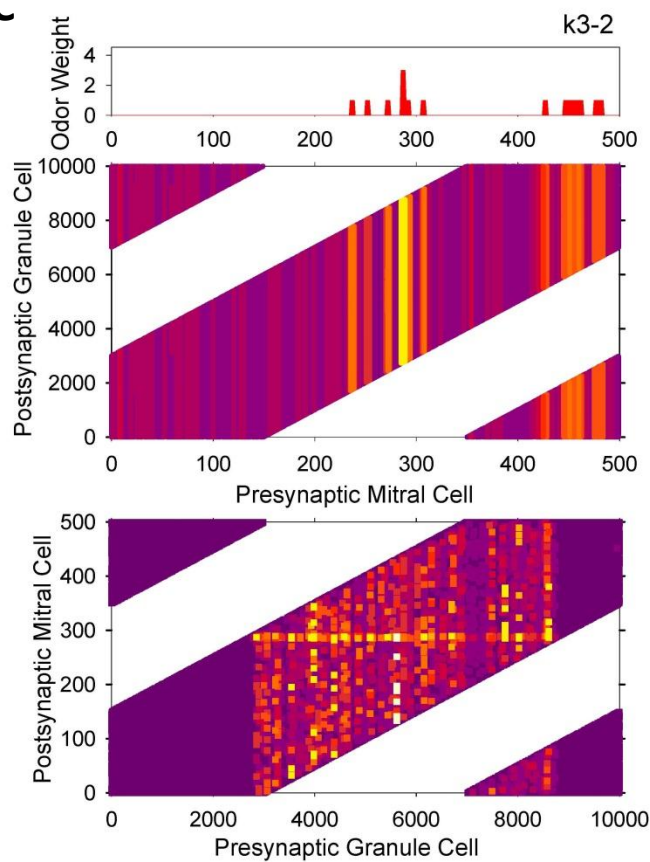**D**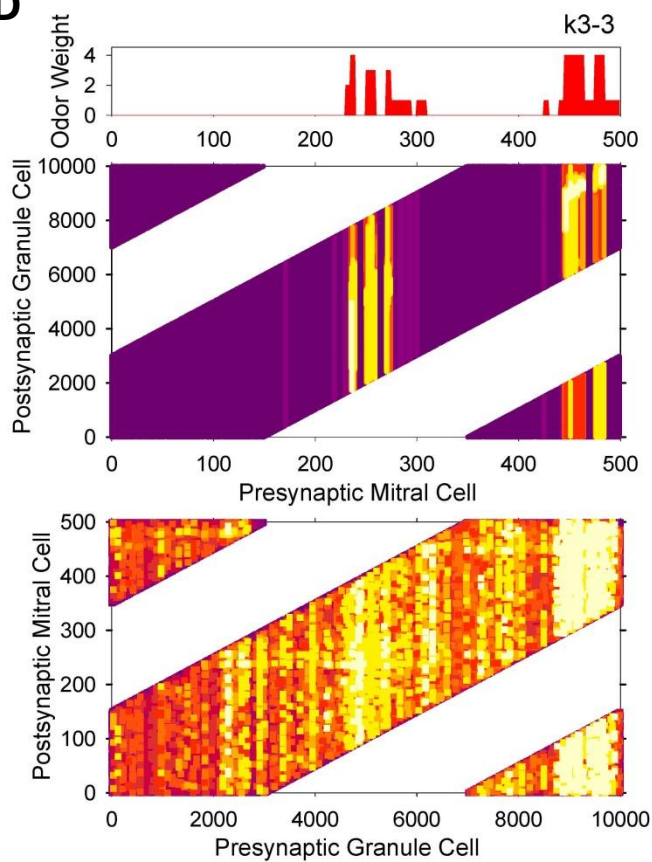

**A**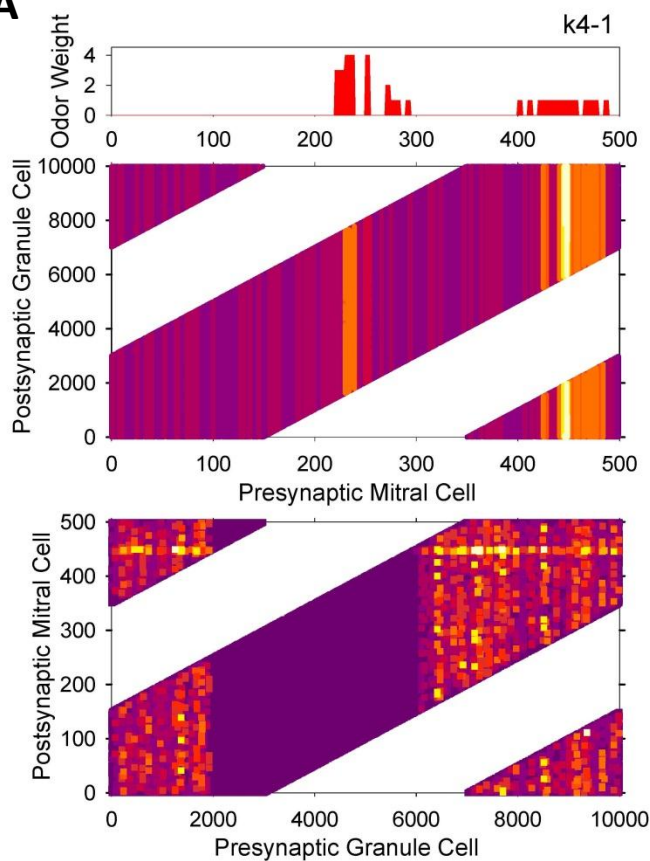**B**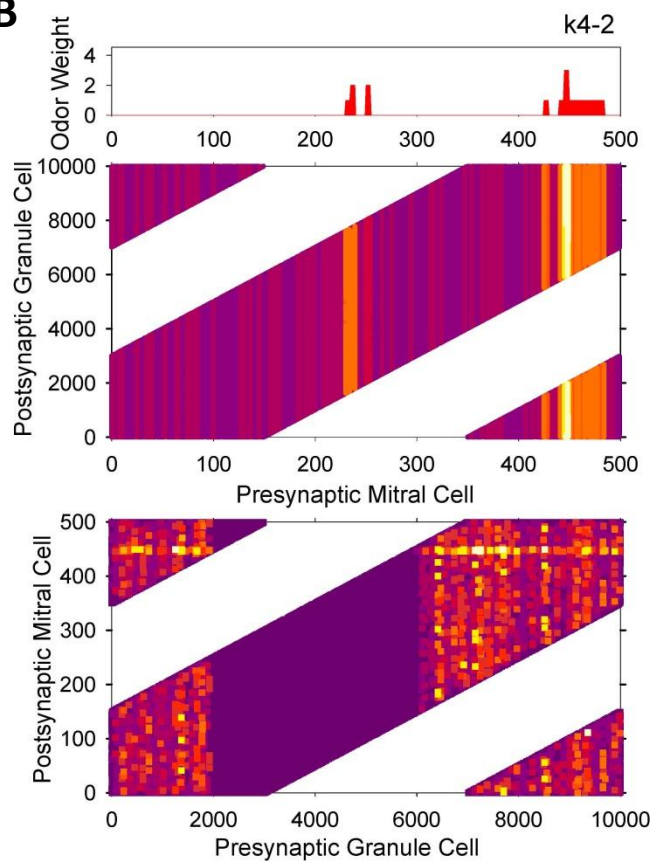**C**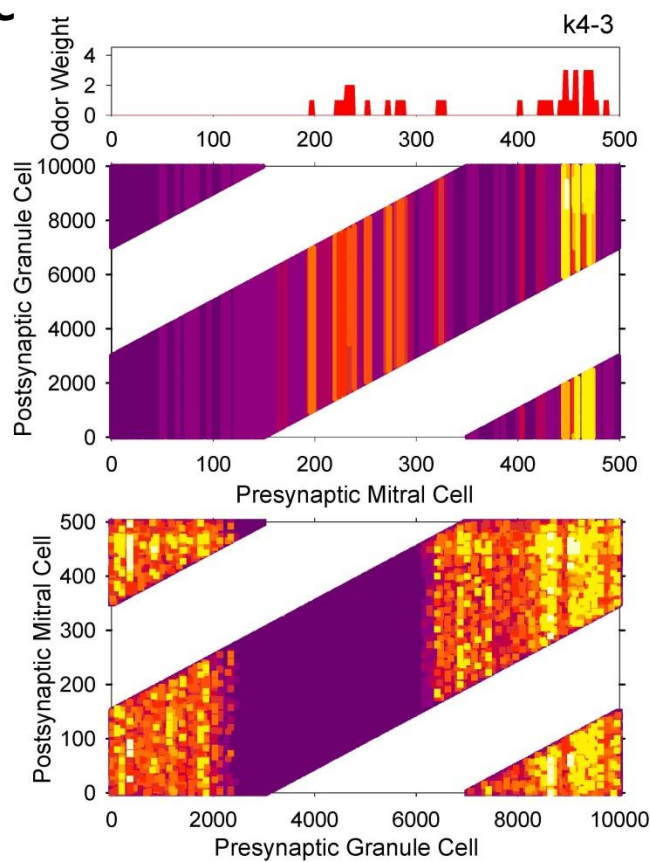**D**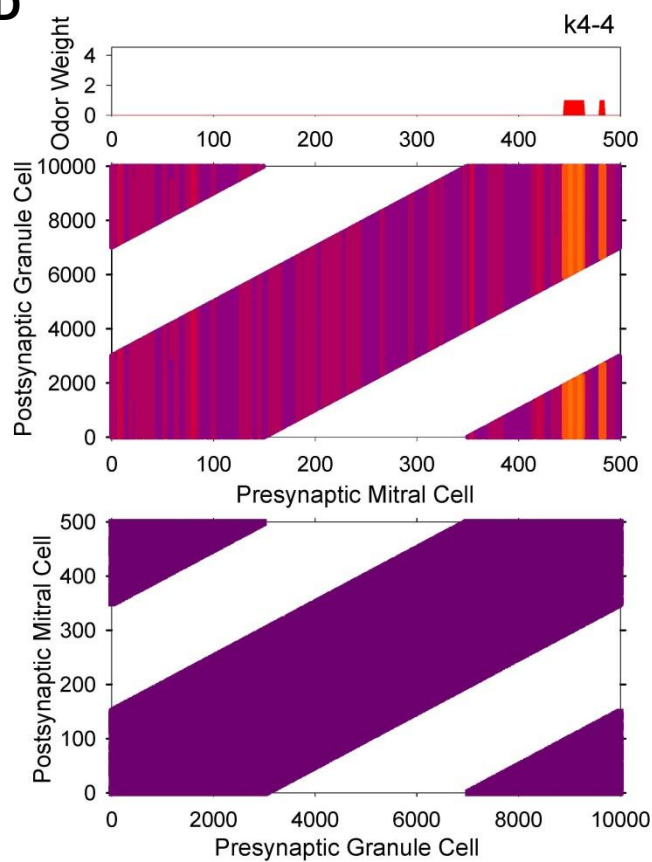

**A**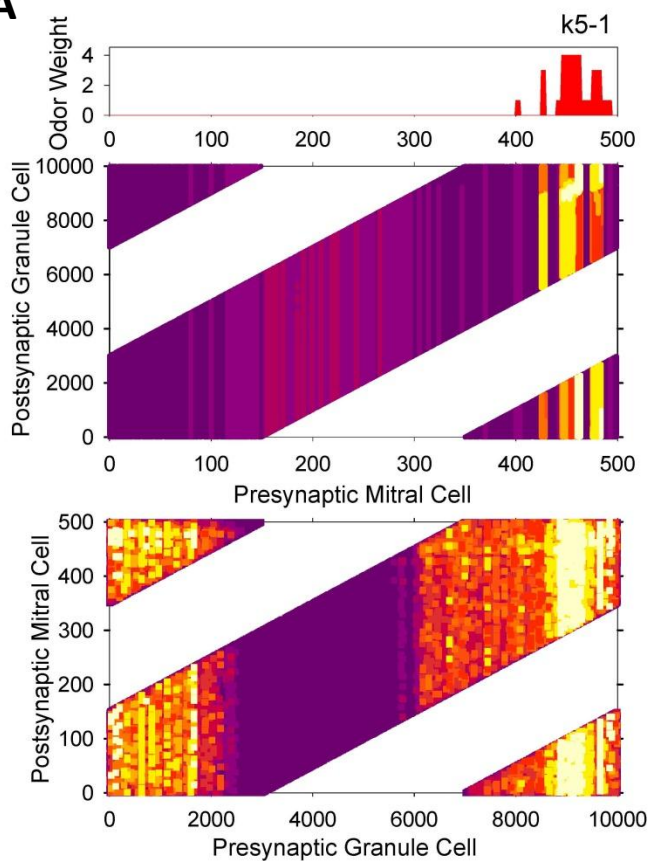**B**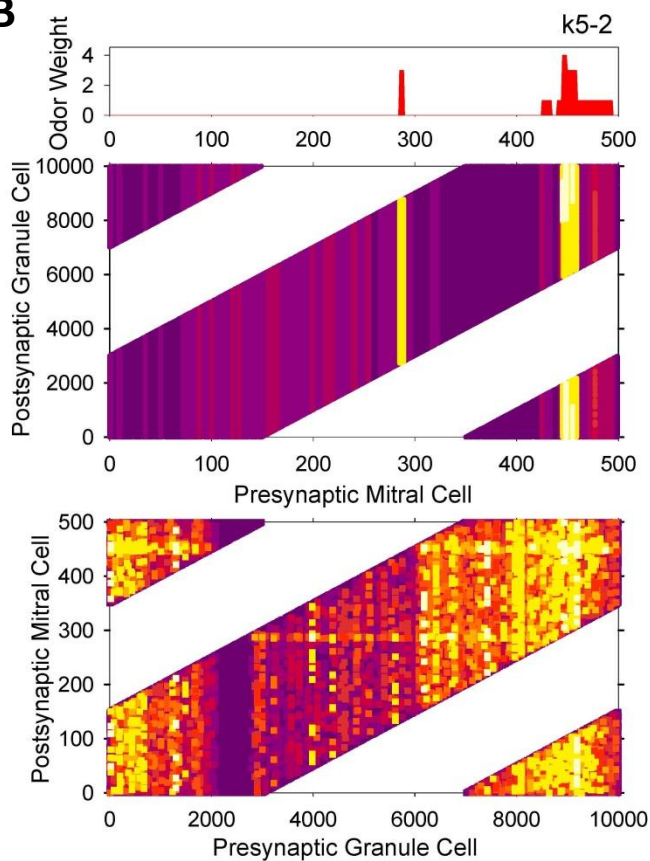**C**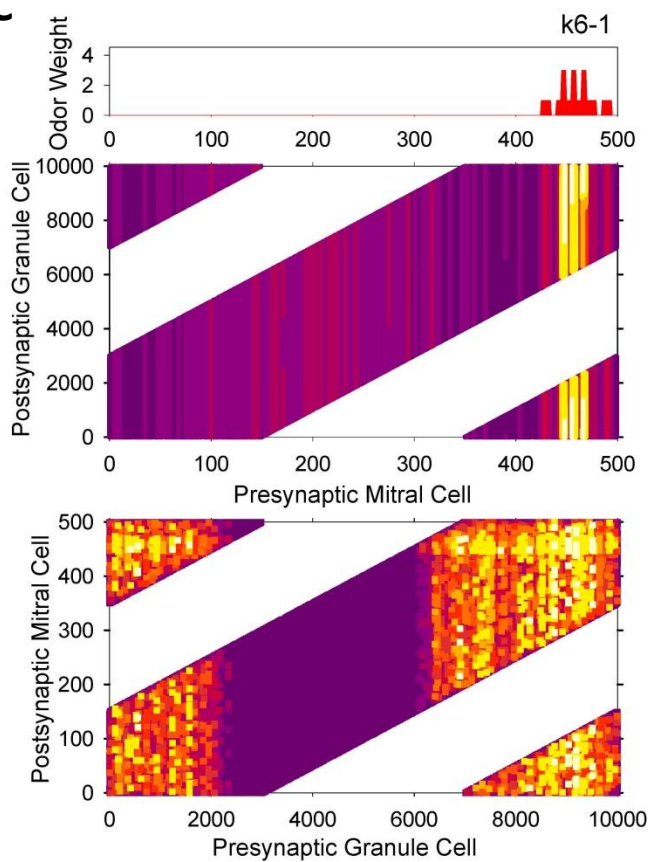**D**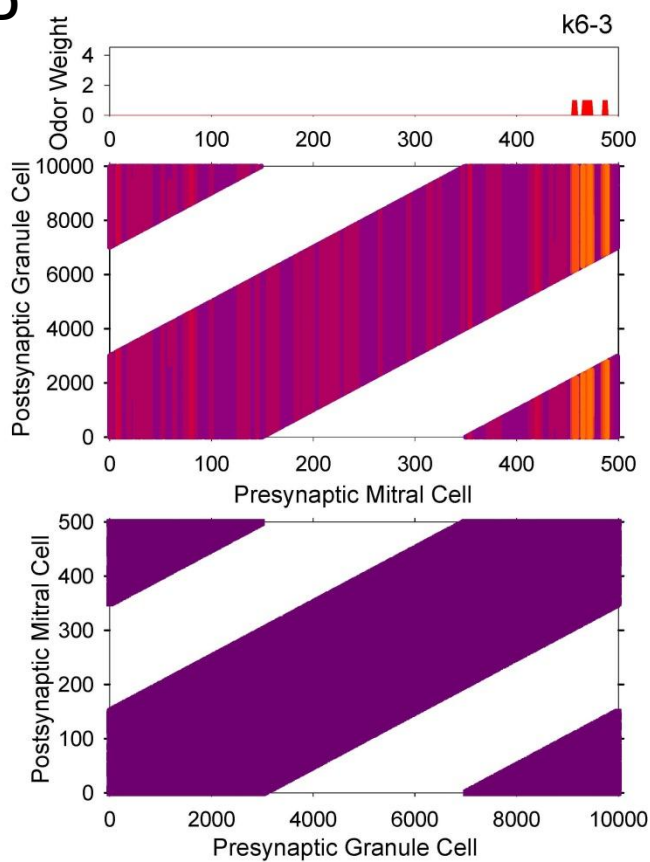

**A**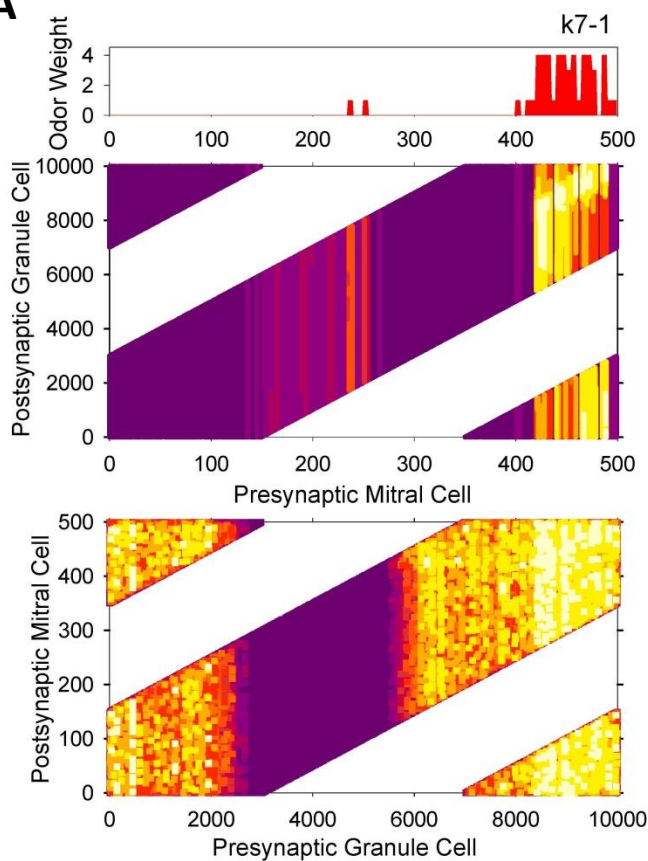**B**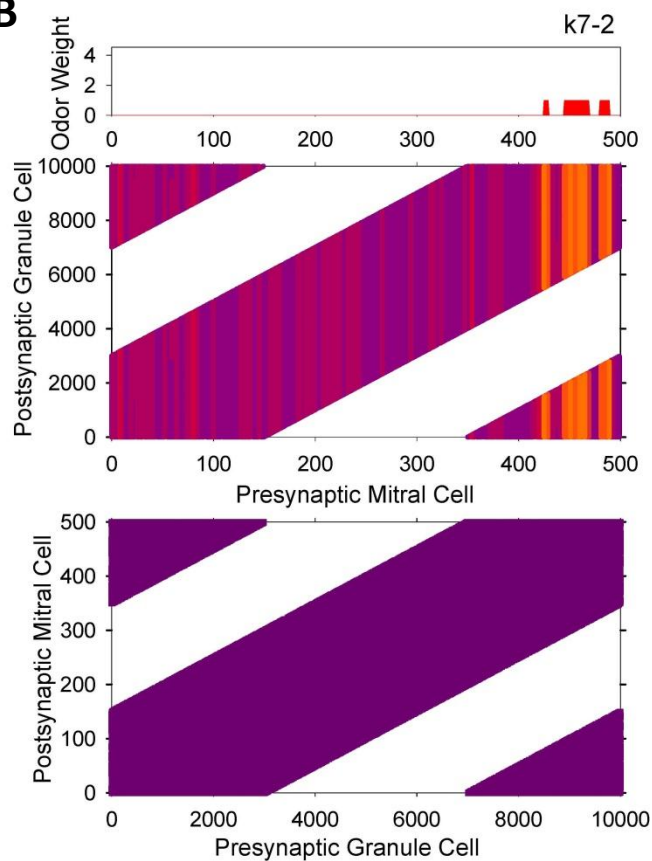**C**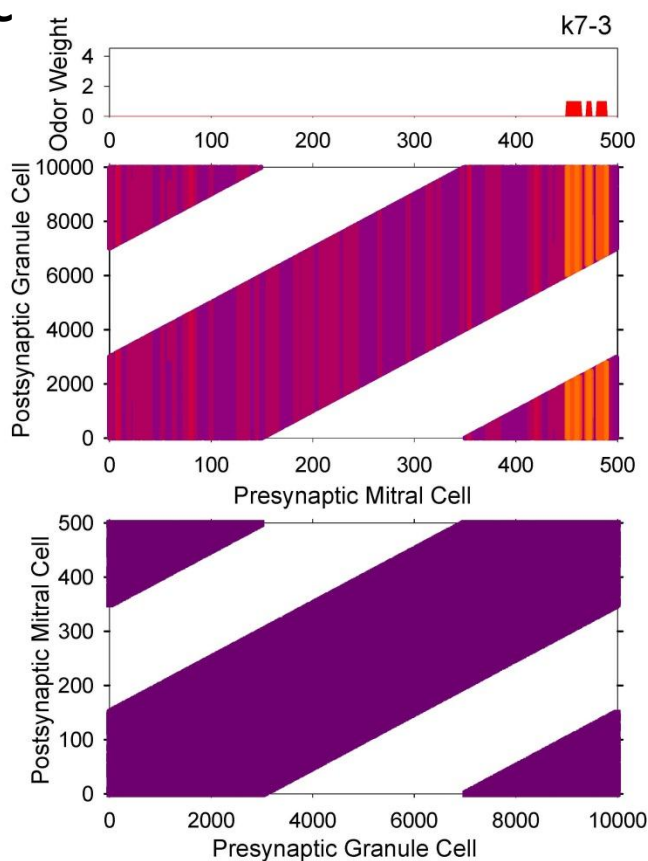**D**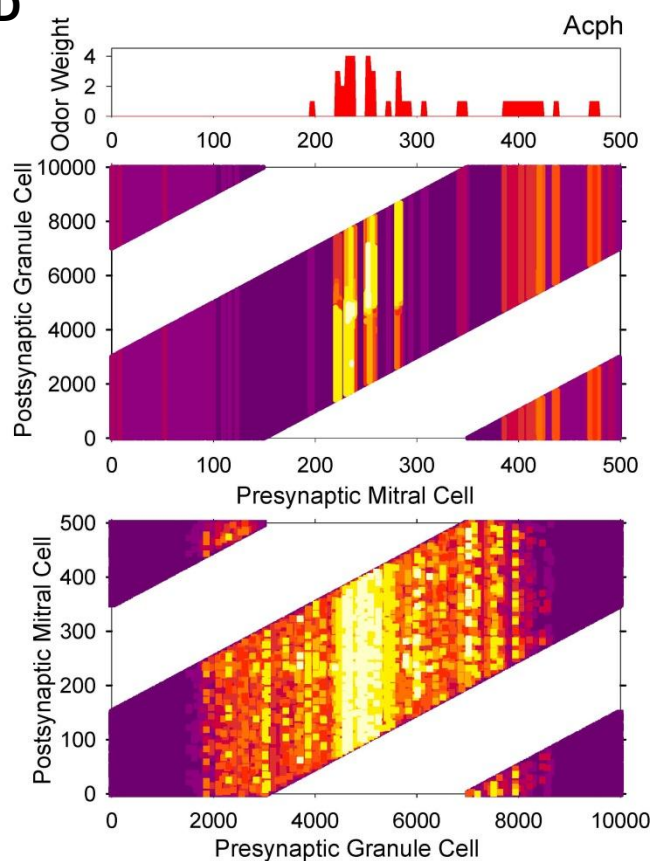

**A**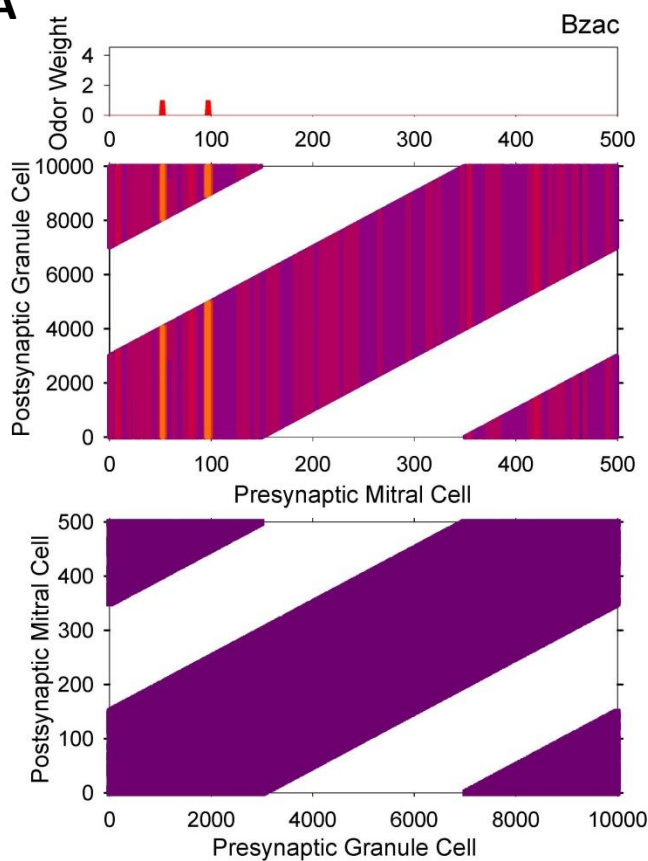**B**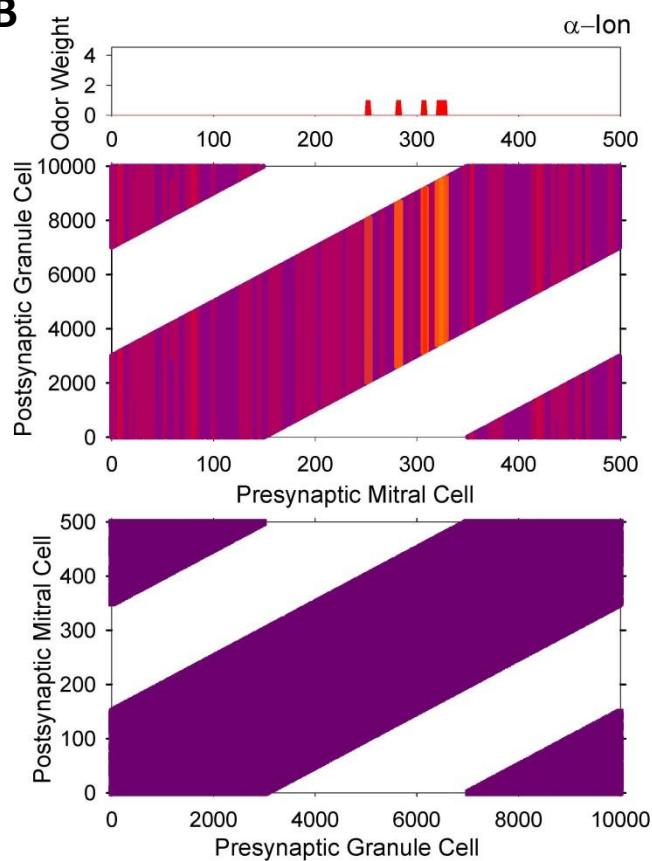**C**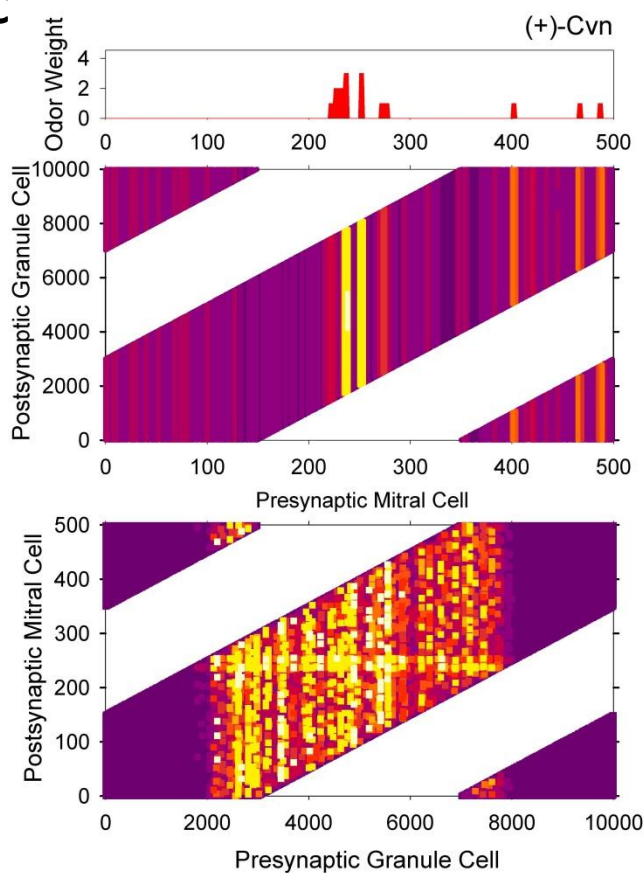**D**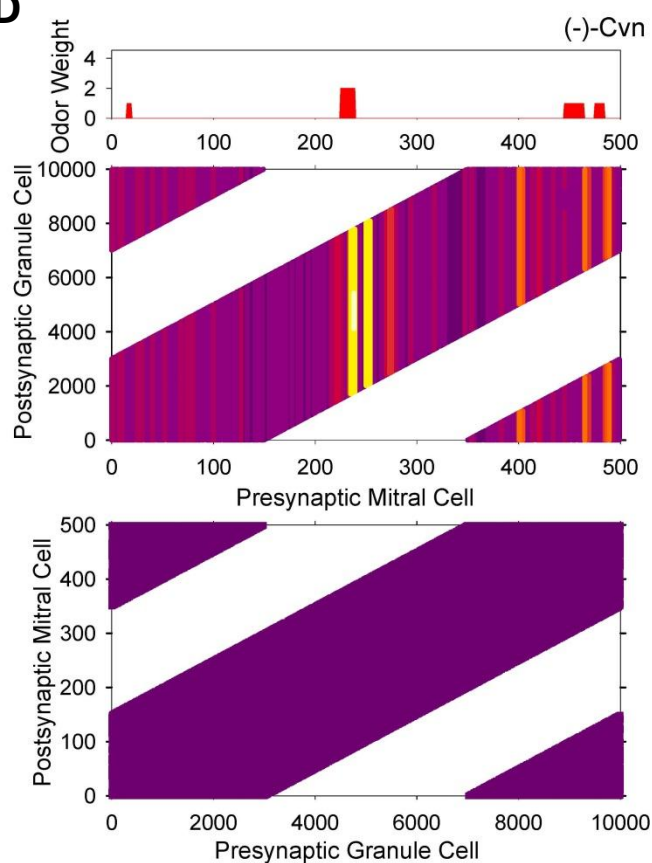

**A**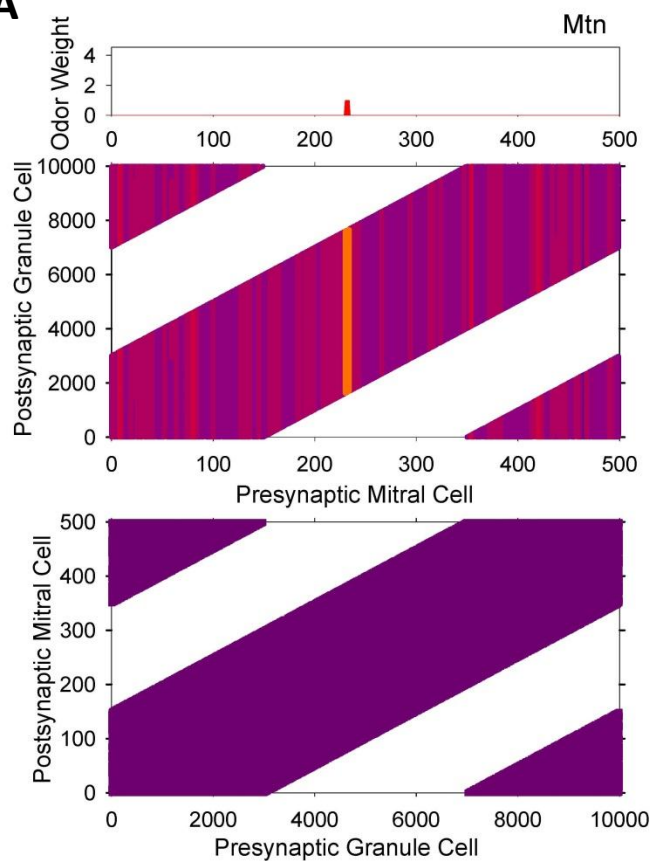**B**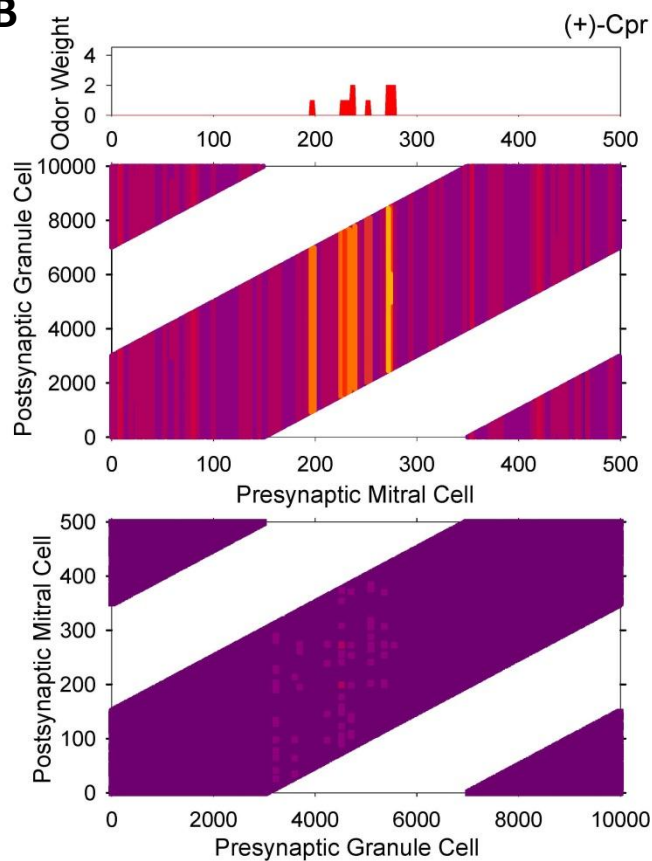**C**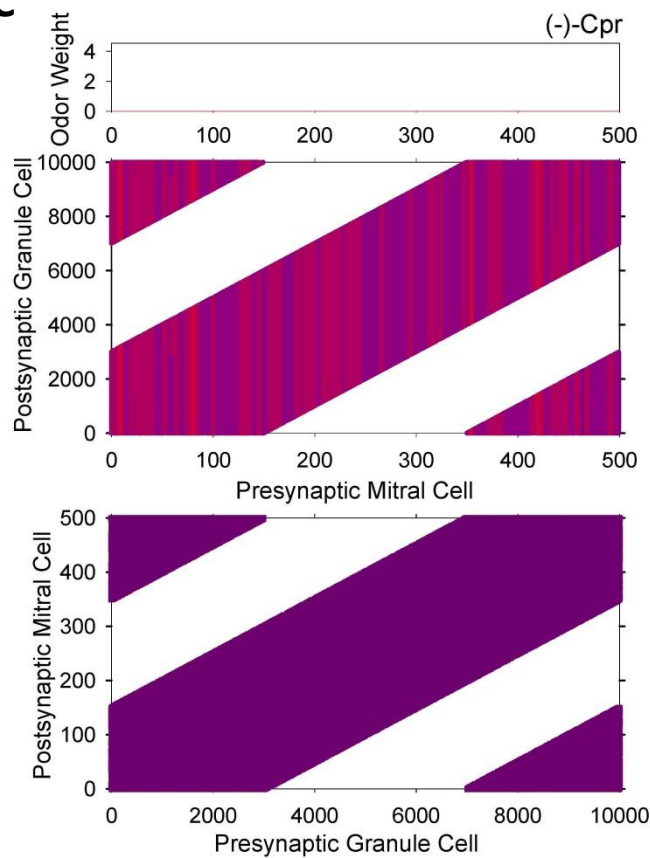**D**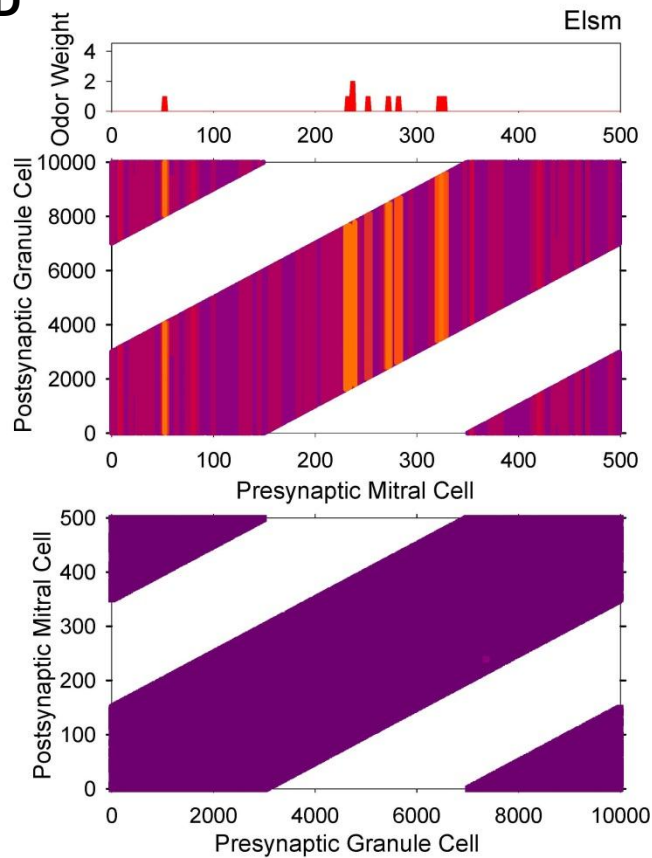

**A**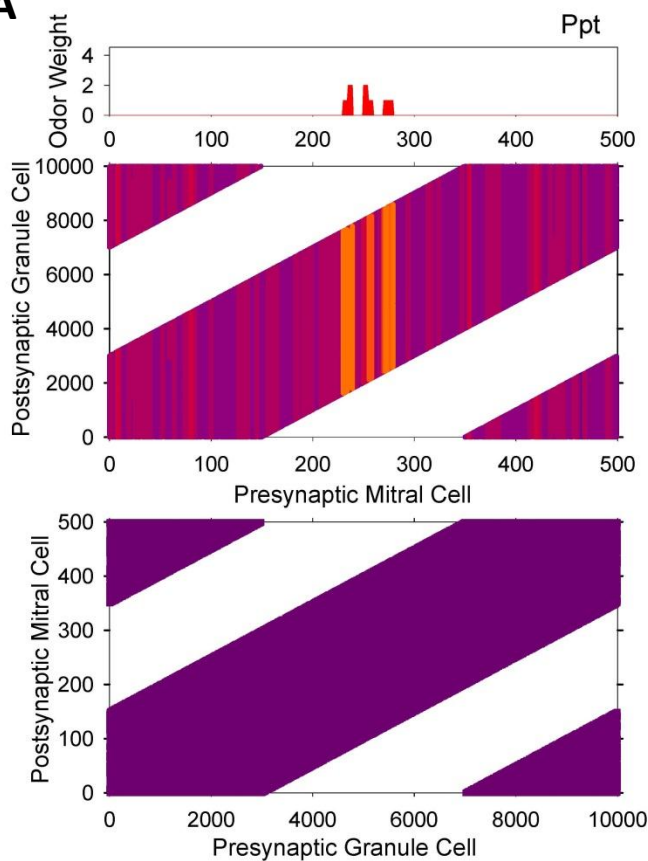**B**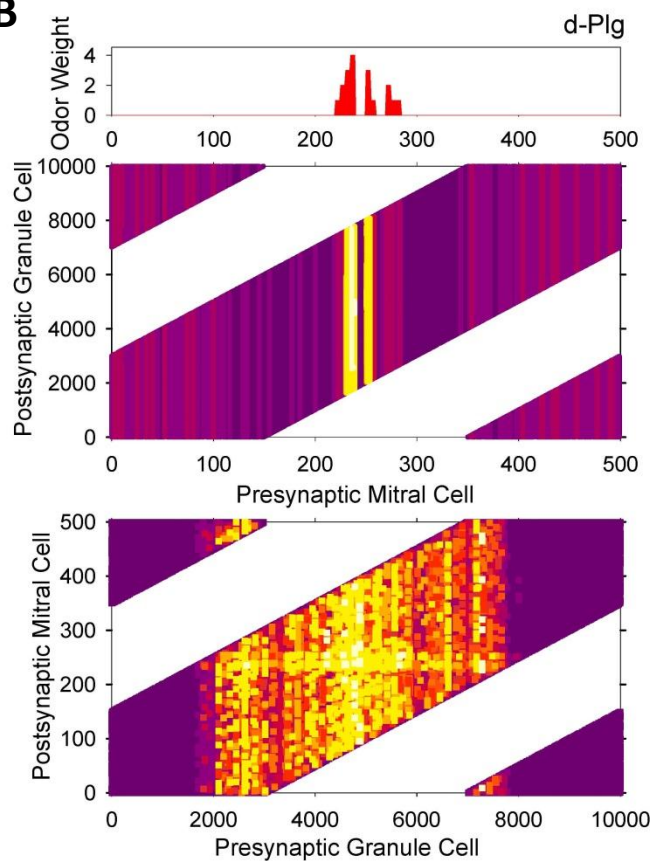**C**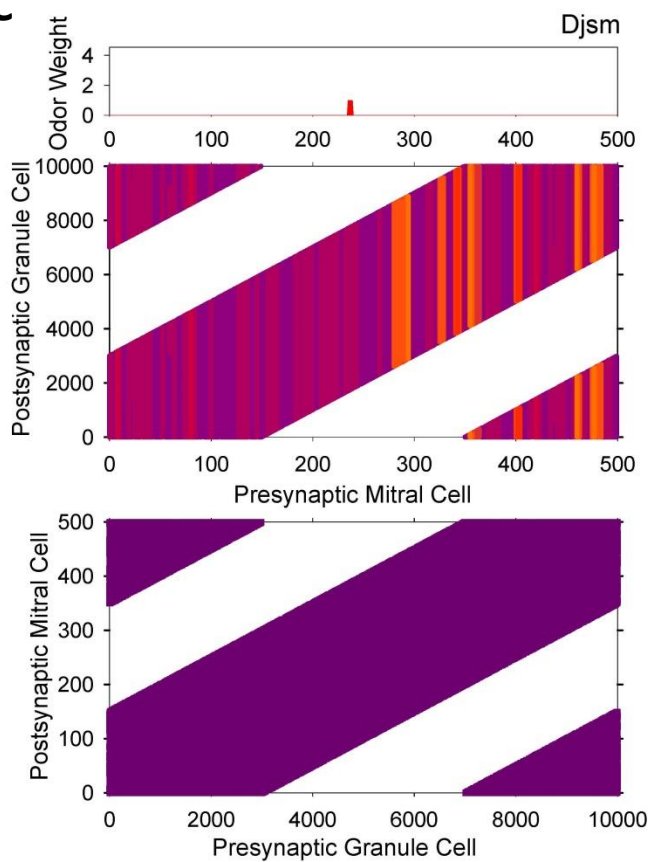**D**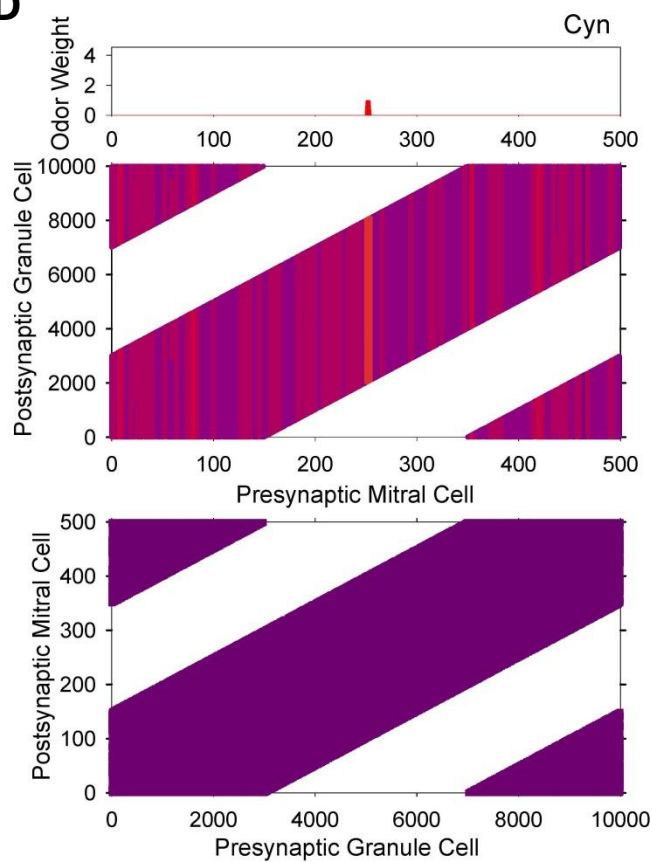

**A**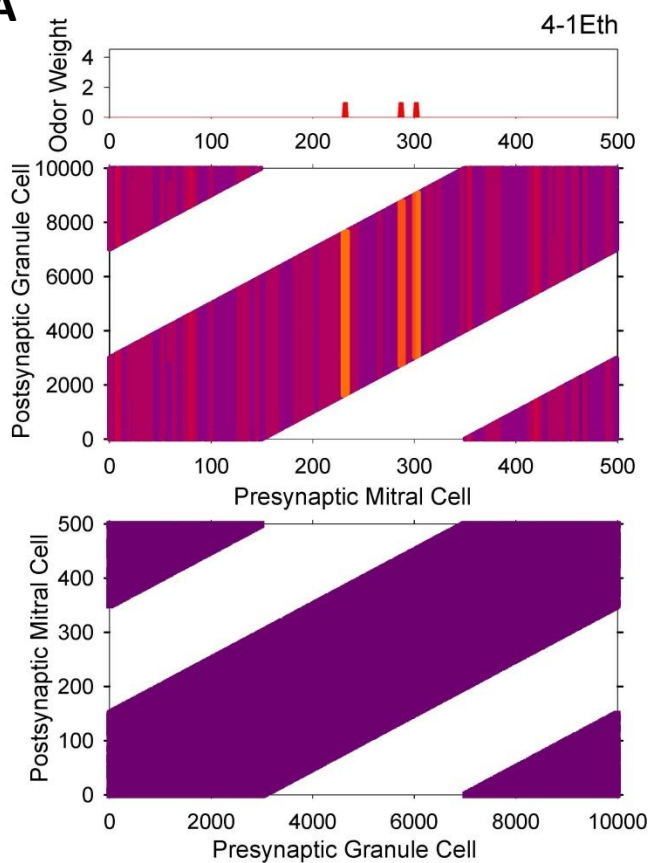**B**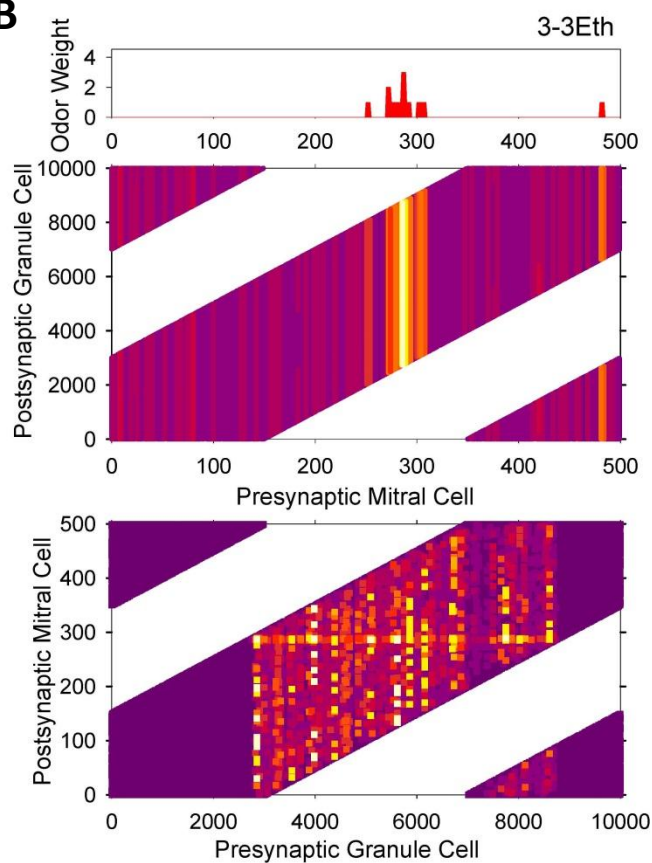**C**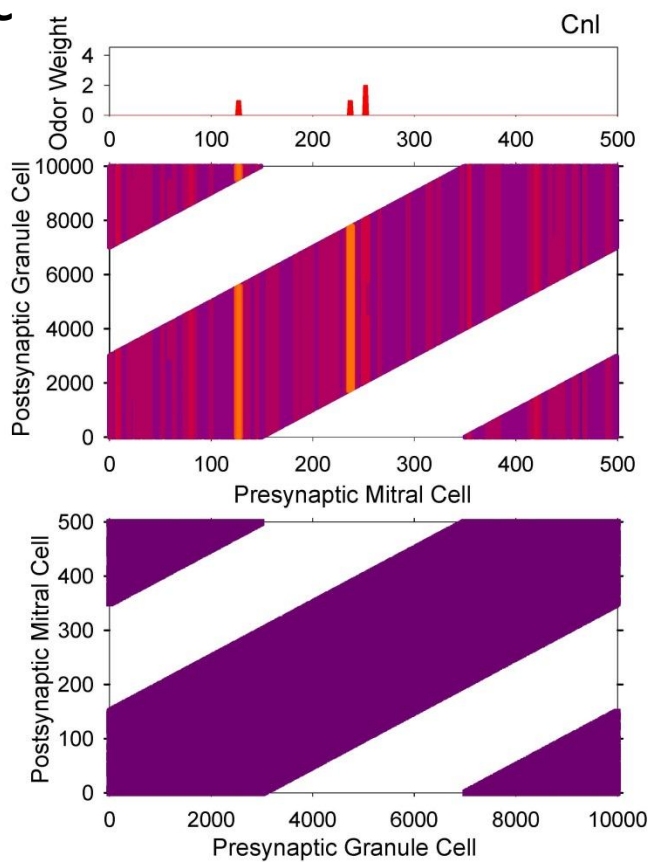**D**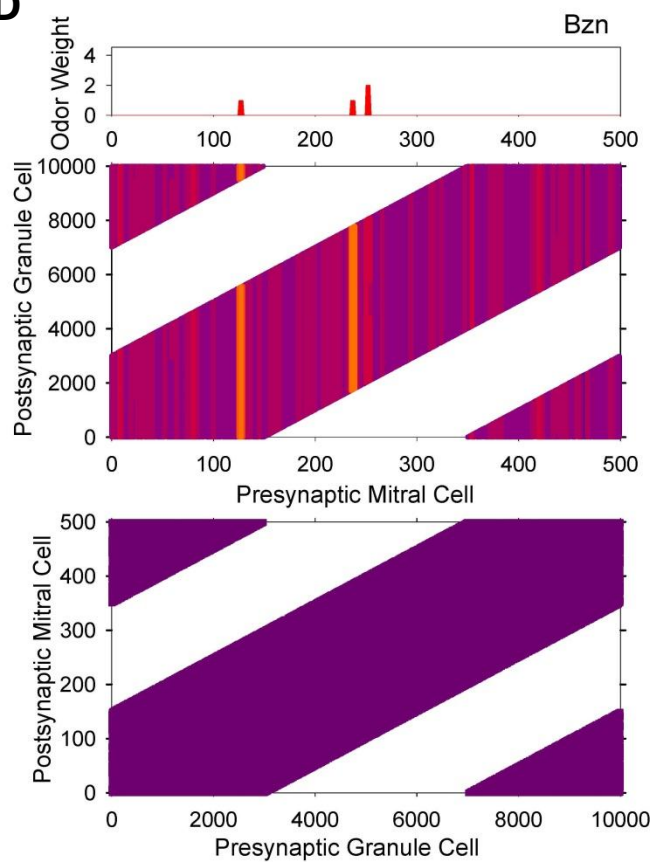

**A**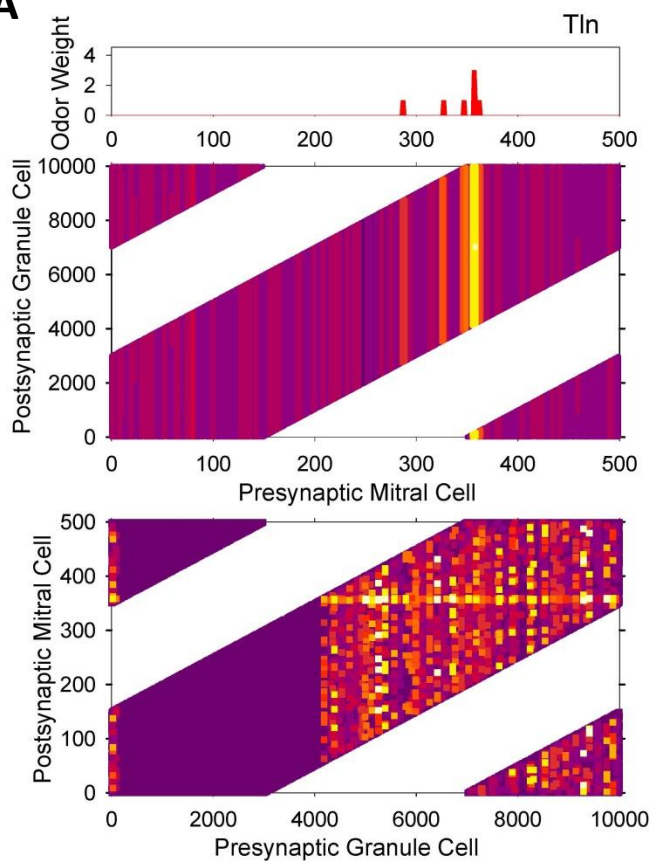**B**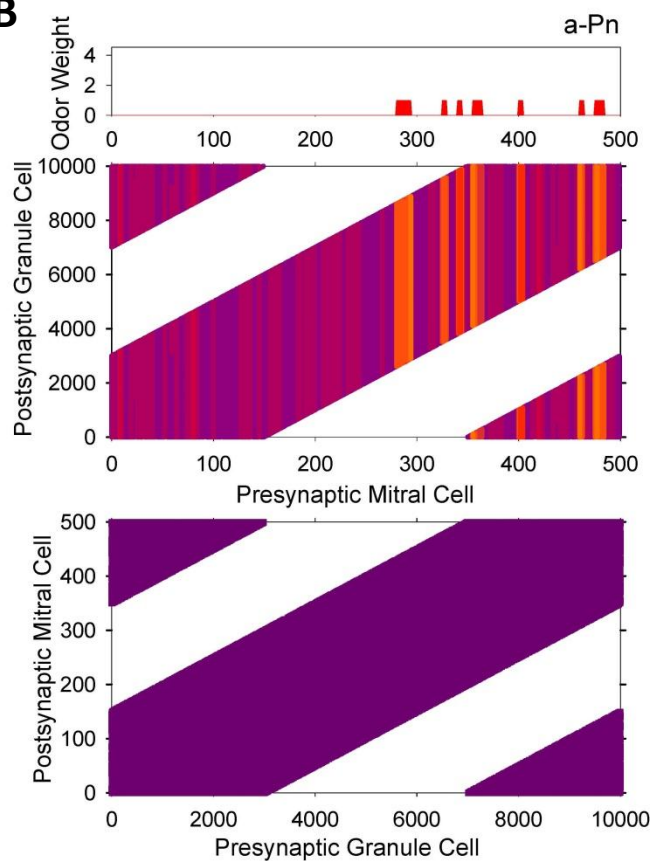**C**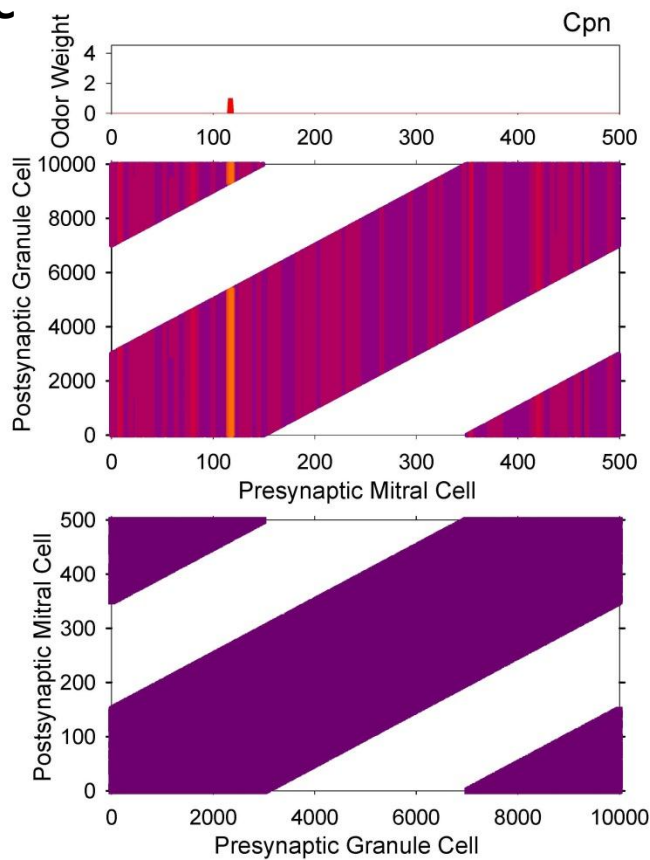**D**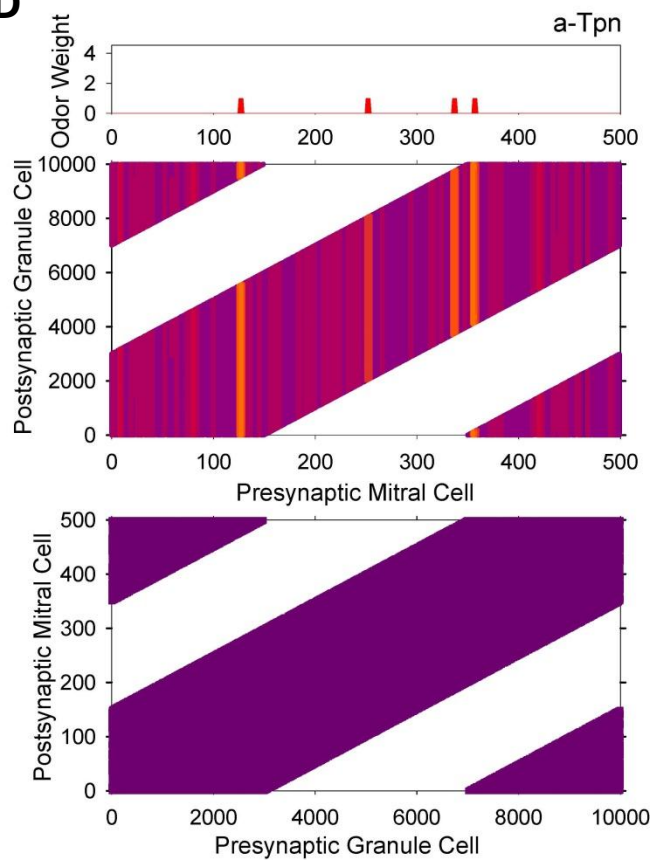

Supplement: Figure S1 — Synaptic weights between mitral and granule cells after 10 sec odor presentations for each of the 72 odors used in this paper. A–D) panels presents the results for a single odor. In each panel, the top histogram represents the input strength on each mitral cell, and the middle and bottom plots represent the normalized excitatory and inhibitory peak synaptic conductance after 10 sec of odor presentation, respectively; (dark purple: 0, white: 1). (PDF) [file pcbi.1003014.s001.pdf]

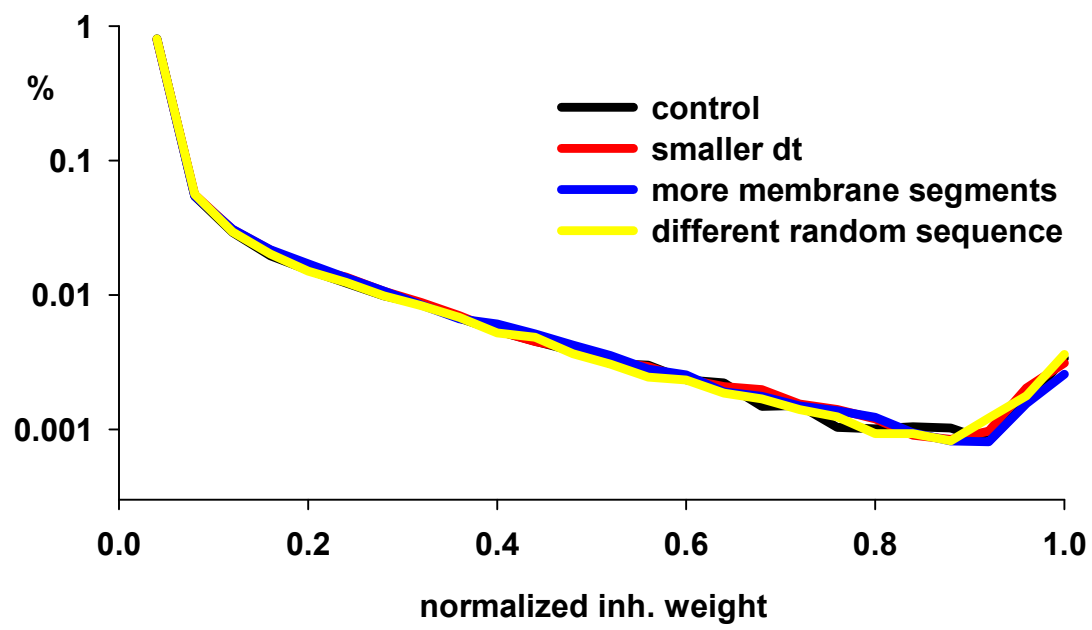

Figure S2

Supplement: Figure S2 — Results from test simulations of odor k3-3 for 10 sec, using the control values used for all simulations, a smaller time step (10 µs instead of 25), modeled with a larger number of compartments (5 µm membrane segments, instead of 10–30 µm), or a different random number sequence. (PDF) [file pcbi.1003014.s002.pdf]
